# Supplementary material for: Population heterogeneity in associations between hormonal contraception and antidepressant use in Sweden: a prospective cohort study applying intersectional multilevel analysis of individual heterogeneity and discriminatory accuracy (MAIHDA)
Source: BMJ Open. 2021 Oct 1;11(10):e049553. doi: 10.1136/bmjopen-2021-049553 (PMC8488727; doi:10.1136/bmjopen-2021-049553)
Supplement: Supplementary data [file bmjopen-2021-049553supp001.pdf]

```
* Hormonal Contraception and Antidepressant Use in Sweden: An
Intersectional Multilevel Analysis of Individual Heterogeneity and
Discriminatory Accuracy
* (MAIHDA)
***************************************************************************
clear *
global MLwiN_path "C:\Program Files\MLwiN v3.05\mlwin.exe"
set cformat %9.2f



***************************************************************************
* TABLE 1
***************************************************************************

* Load the data
use "final_mlMENTAL.dta", clear
keep age_cat1 age_cat2 age_cat3 inc1 inc2 inc3 imm pp proportion denom
order age_cat1 age_cat2 age_cat3 inc1 inc2 inc3 imm pp proportion denom

generate percentage = 100*proportion
drop proportion
format %9.2f percentage

generate age_cat = .
replace age_cat = 1 if age_cat1==1
replace age_cat = 2 if age_cat2==1
replace age_cat = 3 if age_cat3==1

generate inc_cat = .
replace inc_cat = 1 if inc1==1
replace inc_cat = 2 if inc2==1
replace inc_cat = 3 if inc3==1

* Results for the table
tabulate pp [fweight = denom]
table pp [fweight = denom], contents(mean percentage )
tabulate age_cat pp [fweight = denom], column nofreq
tabulate inc_cat pp [fweight = denom], column nofreq
tabulate imm pp [fweight = denom], column nofreq



***************************************************************************
* TABLE 2: MODEL 1
***************************************************************************

* Load the data
use "final_mlMENTAL.dta", clear

* IGLS estimation, for MCMC initial values
runmlwin prop cons, ///
  level2(inter: cons) ///
  level1(inter:) ///
  discrete(distribution(binomial) link(logit) denom(denom) mql1) ///
  nopause

* MCMC
runmlwin prop cons, ///
  level2(inter: cons, residuals(u, savechains("m1u.dta",replace))) ///
  level1(inter:) ///
```

```
  discrete(distribution(binomial) link(logit) denom(denom)) ///
  mcmc(burnin(10000) chain(50000) thin(10) savechains("m1b.dta", replace))
///
  initsprevious ///
  nopause

* Level-2 variance
scalar m1sigma2u = [RP2]var(cons)
scalar list m1sigma2u

* Level-1 variance
scalar m1sigma2e = _pi^2/3
scalar list m1sigma2e

* VPC
display "VPC_u = " %9.4f m1sigma2u/(m1sigma2u + m1sigma2e)

* Compress and save the data
compress
save "m1.dta", replace



*------------------------------------------------------------------------*
* PREPARE FIXED-PART PAREMETER CHAINS
*------------------------------------------------------------------------*

use "m1b.dta", clear
drop deviance RP2_var_cons_ OD_bcons_1
rename FP1_* b_*
format %9.2f b_*
compress
save "m1b_prepped.dta", replace
isid iteration
codebook iteration, compact



*-------------------------------------------------------------------------*
* PREPARE RANDOM EFFECTS CHAINS
*-------------------------------------------------------------------------*
use "m1u.dta", clear
drop residual idnum
rename value u
format %9.2f u
sort inter iteration
order inter iteration
compress
save "m1u_prepped.dta", replace
isid inter iteration
codebook iteration, compact



*-------------------------------------------------------------------------*
* MERGE DATA, FIXED-PART PARAMETER AND RANDOM EFFECT CHAINS TOGETHER
*-------------------------------------------------------------------------*
use "final_mlMENTAL", clear
count
cross using "m1b_prepped.dta"
count
```

```
merge m:1 inter iteration using "m1u_prepped.dta", nogenerate assert(match)
count
compress
save "m1data_prepped.dta", replace



*---------------------------------------------------------------------------*
* ROC
*---------------------------------------------------------------------------*
use "m1data_prepped.dta", clear
count
generate p = invlogit(b_cons + u)
gcollapse (mean) p, by(inter num denom)
count
expand denom
sort inter
bysort inter: generate y = (_n<=numerator)
generate prop = denom/_N
generate weight = int(1/prop)
roctab y p [fw=weight]



*---------------------------------------------------------------------------*
* TABLE 3
*---------------------------------------------------------------------------*
use "m1data_prepped.dta", clear
keep iteration inter age_cat1 age_cat2 age_cat3 inc1 inc2 inc3 imm pp denom
b_cons u
count
generate p = 100*invlogit(b_cons + u)
drop b_cons u
format %9.1f p
drop inter
reshape wide denom p, i(iteration age_cat1 age_cat2 age_cat3 inc1 inc2 inc3
imm) j(pp)
generate denom = denom0 + denom1
drop denom0 denom1
generate pdiff = p1 - p0
gcollapse (mean) p0 p1 pdiff (p2.5) pdifflo=pdiff (p97.5) pdiffhi=pdiff,
by(age_cat1 age_cat2 age_cat3 inc1 inc2 inc3 imm denom)
format %9.1f pdiff pdifflo pdiffhi
order p1 p0 pdiff pdifflo pdiffhi, last
gsort -age_cat1 -age_cat2 -age_cat3 -inc1 -inc2 -inc3 imm



****************************************************************************
* TABLE 2: MODEL 2:
****************************************************************************

* Load the data
use "final_mlMENTAL.dta", clear

* IGLS estimation, for MCMC initial values
runmlwin prop cons age_cat2 age_cat3 inc1 inc2 imm pp, ///
  level2(inter: cons) ///
  level1(inter:) ///
  discrete(distribution(binomial) link(logit) denom(denom) mql1) ///
  nopause
```

```
* MCMC
runmlwin prop cons age_cat2 age_cat3 inc1 inc2 imm  pp, ///
  level2(inter: cons, residuals(u,savechains("m2u.dta",replace))) ///
  level1(inter:) ///
  discrete(distribution(binomial) link(logit) denom(denom)) ///
  mcmc(burnin(10000) chain(50000) thin(10) savechains("m2b.dta", replace))
///
  initsprevious ///
  nopause

* Odds ratios
runmlwin, or

* Level-2 variance
scalar m2sigma2u = [RP2]var(cons)
scalar list m2sigma2u

* Level-1 variance
scalar m2sigma2e = _pi^2/3
scalar list m2sigma2e

* VPC
display "VPC_u = " %9.4f m2sigma2u/(m2sigma2u + m2sigma2e)

* Compress and save the data
compress
save "m2.dta", replace

* PCV
display "PCV = " %9.4f (m2sigma2u - m1sigma2u)/m1sigma2u



*--------------------------------------------------------------------------*
* PREPARE FIXED-PART PAREMETER CHAINS
*--------------------------------------------------------------------------*
use "m2b.dta", clear
drop deviance RP2_var_cons_ OD_bcons_1
rename FP1_* b_*
format %9.2f b_*
compress
save "m2b_prepped.dta", replace
isid iteration
codebook iteration, compact



*--------------------------------------------------------------------------*
* PREPARE inter RANDOM EFFECTS CHAINS
*--------------------------------------------------------------------------*
use "m2u.dta", clear
drop residual idnum
rename value u
format %9.2f u
sort inter iteration
order inter iteration
compress
save "m2u_prepped.dta", replace
isid inter iteration
codebook iteration, compact
```

```
*-------------------------------------------------------------------------*
* MERGE DATA, FIXED-PART PARAMETER AND RANDOM EFFECT CHAINS TOGETHER
*-------------------------------------------------------------------------*
use "final_mlMENTAL", clear
count
cross using "m2b_prepped.dta"
count
merge m:1 inter iteration using "m2u_prepped.dta"
count
save "m2data_prepped.dta", replace



*-------------------------------------------------------------------------*
* ROC
*-------------------------------------------------------------------------*
use "m2data_prepped.dta", clear
count
generate p = invlogit(b_cons + b_age_cat2*age_cat2 + b_age_cat3*age_cat3 +
b_inc1*inc1 + b_inc2*inc2 + b_imm*imm + b_pp*pp)
gcollapse (mean) p, by(inter num denom)
count
expand denom
sort inter
bysort inter: generate y = (_n<=numerator)
generate prop = denom/_N
generate weight = int(1/prop)
roctab y p [fw=weight]



*-------------------------------------------------------------------------*
* TABLE 3
*-------------------------------------------------------------------------*
use "m1data_prepped.dta", clear
keep iteration inter age_cat1 age_cat2 age_cat3 inc1 inc2 inc3 imm pp denom
b_cons u
count
generate p = 100*invlogit(b_cons + u)
drop b_cons u
format %9.1f p
drop inter
reshape wide denom p, i(iteration age_cat1 age_cat2 age_cat3 inc1 inc2 inc3
imm) j(pp)
generate denom = denom0 + denom1
drop denom0 denom1
generate pdiff = p1 - p0
gcollapse (mean) p0 p1 pdiff (p2.5) pdifflo=pdiff (p97.5) pdiffhi=pdiff,
by(age_cat1 age_cat2 age_cat3 inc1 inc2 inc3 imm denom)
format %9.1f pdiff pdifflo pdiffhi
order p1 p0 pdiff pdifflo pdiffhi, last
gsort -age_cat1 -age_cat2 -age_cat3 -inc1 -inc2 -inc3 imm



***************************************************************************
exit
```
