# Supplementary material for: Population heterogeneity in associations between hormonal contraception and antidepressant use in Sweden: a prospective cohort study applying intersectional multilevel analysis of individual heterogeneity and discriminatory accuracy (MAIHDA)
Source: BMJ Open. 2021 Oct 1;11(10):e049553. doi: 10.1136/bmjopen-2021-049553 (PMC8488727; doi:10.1136/bmjopen-2021-049553)
Supplement: Supplementary data [file bmjopen-2021-049553supp003.pdf]

```
pp,imm,inter,age_cat1,age_cat2,age_cat3,inc1,inc2,inc3,proportion,numerator,denom,cons
0,0,12-17 Low income 0 0,1,0,0,1,0,0,.013224002,279,21098,1
1,0,12-17 Low income 0 1,1,0,0,1,0,0,.037408244,265,7084,1
0,1,12-17 Low income 1 0,1,0,0,1,0,0,.0040497542,28,6914,1
1,1,12-17 Low income 1 1,1,0,0,1,0,0,.0096021947,7,729,1
0,0,12-17 Middle income 0 0,1,0,0,0,1,0,.010099272,587,58123,1
1,0,12-17 Middle income 0 1,1,0,0,0,1,0,.030316716,537,17713,1
0,1,12-17 Middle income 1 0,1,0,0,0,1,0,.0056107035,52,9268,1
1,1,12-17 Middle income 1 1,1,0,0,0,1,0,.017814728,15,842,1
0,0,12-17 High income 0 0,1,0,0,0,0,1,.008893352,859,96589,1
1,0,12-17 High income 0 1,1,0,0,0,0,1,.01951286,572,29314,1
0,1,12-17 High income 1 0,1,0,0,0,0,1,.0076045627,30,3945,1
1,1,12-17 High income 1 1,1,0,0,0,0,1,.025718609,17,661,1
0,0,18-23 Low income 0 0,0,1,0,1,0,0,.029676914,530,17859,1
1,0,18-23 Low income 0 1,0,1,0,1,0,0,.034916617,938,26864,1
0,1,18-23 Low income 1 0,0,1,0,1,0,0,.011607248,98,8443,1
1,1,18-23 Low income 1 1,0,1,0,1,0,0,.022702307,62,2731,1
0,0,18-23 Middle income 0 0,0,1,0,0,1,0,.027664155,771,27870,1
1,0,18-23 Middle income 0 1,0,1,0,0,1,0,.0282459,1247,44148,1
0,1,18-23 Middle income 1 0,0,1,0,0,1,0,.011609907,75,6460,1
1,1,18-23 Middle income 1 1,0,1,0,0,1,0,.023316063,54,2316,1
0,0,18-23 High income 0 0,0,1,0,0,0,1,.023347162,1058,45316,1
1,0,18-23 High income 0 1,0,1,0,0,0,1,.022887168,2082,90968,1
0,1,18-23 High income 1 0,0,1,0,0,0,1,.017995911,44,2445,1
1,1,18-23 High income 1 1,0,1,0,0,0,1,.019577537,38,1941,1
0,0,24-30 Low income 0 0,0,0,1,1,0,0,.032189574,2168,67351,1
1,0,24-30 Low income 0 1,0,0,1,1,0,0,.031126546,1954,62776,1
0,1,24-30 Low income 1 0,0,0,1,1,0,0,.013751426,446,32433,1
1,1,24-30 Low income 1 1,0,0,1,1,0,0,.026964672,187,6935,1
0,0,24-30 Middle income 0 0,0,0,1,0,1,0,.030455342,818,26859,1
1,0,24-30 Middle income 0 1,0,0,1,0,1,0,.03591495,652,18154,1
0,1,24-30 Middle income 1 0,0,0,1,0,1,0,.023714487,202,8518,1
1,1,24-30 Middle income 1 1,0,0,1,0,1,0,.027789129,68,2447,1
0,0,24-30 High income 0 0,0,0,1,0,0,1,.025993951,593,22813,1
1,0,24-30 High income 0 1,0,0,1,0,0,1,.024208747,501,20695,1
0,1,24-30 High income 1 0,0,0,1,0,0,1,.023088569,61,2642,1
1,1,24-30 High income 1 1,0,0,1,0,0,1,.019407559,19,979,1
```
