# Supplementary material for: Population heterogeneity in associations between hormonal contraception and antidepressant use in Sweden: a prospective cohort study applying intersectional multilevel analysis of individual heterogeneity and discriminatory accuracy (MAIHDA)
Source: BMJ Open. 2021 Oct 1;11(10):e049553. doi: 10.1136/bmjopen-2021-049553 (PMC8488727; doi:10.1136/bmjopen-2021-049553)
Supplement: Supplementary data [file bmjopen-2021-049553supp004.pdf]

```
pp,imm,inter,age_cat1,age_cat2,age_cat3,inc1,inc2,inc3,proportion,numerator,denom,cons
0,0,12-17 Low income 0 0,1,0,0,1,0,0,.22574355,463,2051,1
1,0,12-17 Low income 0 1,1,0,0,1,0,0,.3049593,412,1351,1
0,1,12-17 Low income 1 0,1,0,0,1,0,0,.12383901,40,323,1
1,1,12-17 Low income 1 1,1,0,0,1,0,0,.1891892,21,111,1
0,0,12-17 Middle income 0 0,1,0,0,0,1,0,.23362993,1024,4383,1
1,0,12-17 Middle income 0 1,1,0,0,0,1,0,.31201944,771,2471,1
0,1,12-17 Middle income 1 0,1,0,0,0,1,0,.13422818,60,447,1
1,1,12-17 Middle income 1 1,1,0,0,0,1,0,.18032786,22,122,1
0,0,12-17 High income 0 0,1,0,0,0,0,1,.28093326,1469,5229,1
1,0,12-17 High income 0 1,1,0,0,0,0,1,.34217408,916,2677,1
0,1,12-17 High income 1 0,1,0,0,0,0,1,.18867925,50,265,1
1,1,12-17 High income 1 1,1,0,0,0,0,1,.3018868,32,106,1
0,0,18-23 Low income 0 0,0,1,0,1,0,0,.37809917,2013,5324,1
1,0,18-23 Low income 0 1,0,1,0,1,0,0,.39212543,2201,5613,1
0,1,18-23 Low income 1 0,0,1,0,1,0,0,.19350649,149,770,1
1,1,18-23 Low income 1 1,0,1,0,1,0,0,.28291318,101,357,1
0,0,18-23 Middle income 0 0,0,1,0,0,1,0,.36302635,2164,5961,1
1,0,18-23 Middle income 0 1,0,1,0,0,1,0,.37776819,2627,6954,1
0,1,18-23 Middle income 1 0,0,1,0,0,1,0,.19285715,108,560,1
1,1,18-23 Middle income 1 1,0,1,0,0,1,0,.27112675,77,284,1
0,0,18-23 High income 0 0,0,1,0,0,0,1,.39782199,2959,7438,1
1,0,18-23 High income 0 1,0,1,0,0,0,1,.38269973,3765,9838,1
0,1,18-23 High income 1 0,0,1,0,0,0,1,.25,82,328,1
1,1,18-23 High income 1 1,0,1,0,0,0,1,.27906978,84,301,1
0,0,24-30 Low income 0 0,0,0,1,1,0,0,.49862742,9082,18214,1
1,0,24-30 Low income 0 1,0,0,1,1,0,0,.50085437,5569,11119,1
0,1,24-30 Low income 1 0,0,0,1,1,0,0,.32457545,1013,3121,1
1,1,24-30 Low income 1 1,0,0,1,1,0,0,.37422037,360,962,1
0,0,24-30 Middle income 0 0,0,0,1,0,1,0,.50859779,2869,5641,1
1,0,24-30 Middle income 0 1,0,0,1,0,1,0,.49799198,1488,2988,1
0,1,24-30 Middle income 1 0,0,0,1,0,1,0,.37214136,358,962,1
1,1,24-30 Middle income 1 1,0,0,1,0,1,0,.33590734,87,259,1
0,0,24-30 High income 0 0,0,0,1,0,0,1,.48993289,1971,4023,1
1,0,24-30 High income 0 1,0,0,1,0,0,1,.48666918,1296,2663,1
0,1,24-30 High income 1 0,0,0,1,0,0,1,.37669376,139,369,1
1,1,24-30 High income 1 1,0,0,1,0,0,1,.45238096,57,126,1
```
