# Supplementary material for: Population heterogeneity in associations between hormonal contraception and antidepressant use in Sweden: a prospective cohort study applying intersectional multilevel analysis of individual heterogeneity and discriminatory accuracy (MAIHDA)
Source: BMJ Open. 2021 Oct 1;11(10):e049553. doi: 10.1136/bmjopen-2021-049553 (PMC8488727; doi:10.1136/bmjopen-2021-049553)
Supplement: Supplementary data [file bmjopen-2021-049553supp005.pdf]

Supplementary material 5

Supplementary table, summary statistics. Numbers are percentages (numbers within brackets).

		Hormonal contraception	Mental health issues
Age	12-17	23.2 (63 181)	7.2 (19 536)
	18-23	59.9 (192 315)	13.6 (43 729)
	24-30	40.3 (130 103)	15.6 (50 447)
Income	Low	40.8 (126 632)	15.9 (49 316)
	Middle	38.9 (98 698)	12.2 (31 032)
	High	45.6 (160 269)	9.5 (33 363)
Immigrant background	No	45.1 (363 390)	12.9 (103 938)
	Yes	20.1 (22 209)	8.9 (9 773)

Supplementary table. Percentage of women within each intersectional dimension using hormonal contraceptives and with previous mental health issues.

Zettermark S, *et al. BMJ Open* 2021; 11:e049553. doi: 10.1136/bmjopen-2021-049553
