# Supplementary material for: Population heterogeneity in associations between hormonal contraception and antidepressant use in Sweden: a prospective cohort study applying intersectional multilevel analysis of individual heterogeneity and discriminatory accuracy (MAIHDA)
Source: BMJ Open. 2021 Oct 1;11(10):e049553. doi: 10.1136/bmjopen-2021-049553 (PMC8488727; doi:10.1136/bmjopen-2021-049553)
Supplement: Supplementary data [file bmjopen-2021-049553supp006.pdf]

BMJ Publishing Group Limited (BMJ) disclaims all liability and responsibility arising from any reliance placed on this supplemental material which has been supplied by the author(s)

Supplementary material 6

ATC	Freq.	Percent
G02BA03	12 535	3.25
G02BB	96	0.02
G0BB01	26 022	6.75
G02BB01	48	0.01
G03AA03	4 786	1.24
G03AA07	126 061	32.69
G03AA09	3 227	0.84
G03AA11	15 463	4.01
G03AA12	4 596	13.69
G03AA13	12 329	1.19
G03AA14	5 958	3.20
G03AB	5 958	1.55
G03AB03	8 014	2.08
G03AB04	5 341	1.39
G03AC01	4 249	1.10
G03AC02	2 483	0.64
G03AC06	2 710	0.70
G03AC08	21 284	5.52
G03AC09	77 595	20.12

Supplementary table, frequency table of hormonal contraceptives. Frequency of all included hormonal contraceptives in the final cohort of 915 954 women.
