# Supplementary material for: Population heterogeneity in associations between hormonal contraception and antidepressant use in Sweden: a prospective cohort study applying intersectional multilevel analysis of individual heterogeneity and discriminatory accuracy (MAIHDA)
Source: BMJ Open. 2021 Oct 1;11(10):e049553. doi: 10.1136/bmjopen-2021-049553 (PMC8488727; doi:10.1136/bmjopen-2021-049553)
Supplement: Supplementary data [file bmjopen-2021-049553supp007.pdf]

Supplementary material 7

Sensitivity analysis only including only new users and never-users of HC. Women with any dispensed prescription of HC during five years prior to baseline were excluded and only women with a HC prescription fill exclusively during follow-up are included as users. Non-users of HC are defined as not filing any prescription of HC during five years prior to baseline or during follow-up.

Table 1. Characteristics of the 532 543 women aged 12 - 30 years by previous mental health issues and use of hormonal contraceptives. Values are percentages (number of women in parenthesis).

	Previous mental health issues			
	Yes 11.12 (59 238)		No 88.88 (473 305)	
	Use of HC		Use of HC	
	Yes 38.87 (23 034)	No 61.13 (36 214)	Yes 1.83 (8 678)	No 98.17 (464 627)
Antidepressant during follow-up	60.11 (35 610)	39.89 (23 629)		
Age				
12-17 years	32.53 (4 065)	25.56 (11 946)	43.24 (38 426)	50.37 (193 658)
18-23 years	37.18 (4 646)	27.22 (12 722)	35.44 (31 489)	20.09 (77 244)
24-30 years	30.28 (3 784)	47.23 (22 075)	21.32 (18 948)	29.53 (113 540)
Income level				
Low inc.	35.93 (4 489)	43.77 (20 458)	24.00 (21 331)	30.34 (116 635)
Medium inc.	30.21 (3 775)	28.13 (13 147)	29.43 (26 151)	30.46 (117 109)
High inc.	33.86 (4 231)	28.11 (13 138)	46.57 (41 381)	39.20 (150 698)
Immigrant background				
None	92.84 (11 600)	88.12 (41 188)	91.78 (81 560)	81.40 (312 926)
Yes	7.16 (895)	11.88 (5 555)	8.22 (7 303)	18.60 (71 516)

Table 2. Results from the Multilevel Analysis of Individual Heterogeneity and Discriminatory Accuracy (MAIHDA) distinguishing between measures of association (Odds Ratios) and measures of variance and discriminatory accuracy. The analyses are stratified by the existence of previous mental issues. Values are point estimations (with 95% credible intervals) or percentages where indicated.

	Without metal health issues		With mental health issues	
	Model 1	Model 2	Model 1	Model 2
**Measures of association**				
Age				
12-17 years		Reference		Reference
18-23 years		1.76 (1.35-2.21)		1.64 (1.42-1.89)
24-30 years		2.34 (1.79-2.90)		2.69 (2.32-3.09)
Income				
High inc.		Reference		Reference
Medium inc.		1.06 (0.81-1.34)		0.84 (0.72-0.98)
Low inc.		1.08 (0.86-1.35)		0.87 (0.75-1.00)
Immigrant background				
None		Reference		Reference
Yes		0.63 (0.51-0.76)		0.52 (0.46-0.59)
Hormonal contraception				
No		Reference		Reference
Yes		1.86 (1.51-2.28)		1.18 (1.05-1.34)
**Measures of variance and discriminatory accuracy***				
Variance	0.36 (0.22-0.60)	0.08 (0.04-0.15)	0.31 (0.19-0.51)	0.02 (0.01-0.14)
VPC	9.88%	2.34%	8.67%	0.63%
PCV		76.32%		92.73%
AUC	0.63 (0.63-0.63)	0.62 (0.62-0.62)	0.65 (0.64-0.65)	0.64 (0.64-0.64)

*Between-strata variance, variance partition coefficient (VPC), proportional change of the variance (PCV), Area under the curve (AUC)

Table 3. Distribution of antidepressant use between different intersectional strata, and difference in usage between user and non-users of hormonal contraceptives but otherwise sharing the same intersectional stratum. The values are calculated from the multilevel analysis of individual heterogeneity and discriminatory accuracy (MAIHDA). Numbers are percentages.

Previous mental health issues	Age (years)	Income level	Immigrant background	Number of women	Use of hormonal contraceptives (%)		
					Yes	No	Yes-No difference
No	12 – 17	Low	No	25342	3.7	1.2	2.4 (1.9-3)
			Yes	7416	1.2	0.4	0.7 (0.1-1.6)
		Middle	No	69096	2.9	1	1.9 (1.6-2.2)
			Yes	9839	2	0.6	1.4 (0.5-2.5)
		High	No	115995	1.9	0.9	1 (0.8-1.2)
			Yes	4396	2.5	0.8	1.7 (0.6-3.2)
	18 – 23	Low	No	15523	3.9	2.7	1.2 (0.5-1.8)
			Yes	8238	2.2	1.1	1 (0.2-2)
		Middle	No	27757	2.9	2.2	0.7 (0.3-1.1)
			Yes	6642	2.2	1.1	1.1 (0.2-2.1)
		High	No	47988	2.3	2	0.3 (0-0.6)
			Yes	2585	2.5	1.8	0.7 (-0.5-2.1)
	24 – 30	Low	No	51819	4.2	3.3	0.9 (0.4-1.3)
			Yes	29628	2.7	1.3	1.4 (0.8-2.1)
		Middle	No	22251	4.7	2.8	1.9 (1.2-2.6)
			Yes	7675	2.8	2.1	0.6 (-0.4-1.8)
		High	No	18715	3.1	2.6	0.6 (0-1.2)
			Yes	2400	2.9	2.3	0.6 (-1-2.7)
Yes	12 – 17	Low	No	2671	30.5	21.8	8.6 (4.9-12.4)
			Yes	372	19.7	12.6	7.1 (-1.3-16.6)
		Middle	No	5554	31.6	22.9	8.7 (6-11.5)
			Yes	507	17.3	14.4	2.9 (-4.8-11.9)
		High	No	6585	35.2	27.9	7.3 (4.6-10)
			Yes	322	29.5	19.6	9.9 (-0.5-21.1)
	18 – 23	Low	No	4197	38.9	39	-0.1 (-3.5-3.4)
			Yes	666	29.2	20.1	9.1 (0.7-17.9)
		Middle	No	5049	38.2	36.4	1.8 (-1.2-4.8)
			Yes	549	31.1	18.6	12.5 (3.2-22.2)

	High	No	6601	39.5	40.6	-1.1 (-3.6-1.6)
		Yes	306	32.7	23.6	9.1 (-1.5-20.1)
24 – 30	Low	No	14408	48.5	50.5	-2 (-4.4-0.3)
		Yes	2633	32.7	32.1	0.6 (-4.9-6.3)
	Middle	No	4486	49.5	50.8	-1.3 (-5.3-2.7)
		Yes	777	34.4	36.5	-2.1 (-11.7-8)
	High	No	3237	46.3	49.3	-3 (-7.6-1.7)
		Yes	318	41.4	36.6	4.8 (-9-19.7)
