# Supplementary material for: Population heterogeneity in associations between hormonal contraception and antidepressant use in Sweden: a prospective cohort study applying intersectional multilevel analysis of individual heterogeneity and discriminatory accuracy (MAIHDA)
Source: BMJ Open. 2021 Oct 1;11(10):e049553. doi: 10.1136/bmjopen-2021-049553 (PMC8488727; doi:10.1136/bmjopen-2021-049553)
Supplement: Supplementary data [file bmjopen-2021-049553supp008.pdf]

Supplementary material 8

Sensitivity analysis only including women with a recent health care contact (defined as any dispensed prescription or appointment at a hospital in the last 3 years) = 60.46% of the original population

Table 1. Characteristics of the 553 789 women aged 12 - 30 years and residing in Sweden by 1st January 2013 by previous mental health issues and use of hormonal contraceptives. Values are percentages (number of women) if not otherwise indicated.

	Previous mental health issues			
	Yes 19.01 (n = 105 283)		No 80.99 (n = 448 506)	
	Use of Hormonal contraceptives		Use of Hormonal contraceptives	
	Yes 42.42 (n = 44657)	No 57.58 (n = 60 626)	Yes 44.41 (n= 199 170)	No 55.59 (n = 249 336)
Antidepressant drugs	41.17 (19 886)	39.77 (26 013)	2.73 (9 215)	1.87 (8 699)
Age				
12-17 years	15.11 (6 747)	20.75 (12 581)	15.63 (31 133)	37.24 (92 846)
18-23 years	48.78 (21 784)	31.29 (18 968)	48.30 (96 200)	22.75 (56 735)
24-30 years	36.11 (16 126)	47.96 (29 077)	36.07 (71 837)	40.01 (99 755)
Income level				
Low	39.70 (17 731)	45.01 (27 286)	33.06 (65 847)	35.08 (87 456)
Middle	27.55 (12 302)	27.85 (16 887)	26.00 (51 776)	29.91 (74 575)
High	32.75 (14 624)	27.14 (16 453)	40.94 (81 547)	35.01 (87 305)
Immigrant background				
No	94.50 (42 200)	88.94 (53 919)	93.76 (186 745)	83.61 (208 465)
Yes	5.50 (2 457)	11.06 (6 707)	6.24 (12 425)	16.39 (40 871)

Table 2. Results from the Multilevel Analysis of Individual Heterogeneity and Discriminatory Accuracy (MAIHDA) distinguishing between measures of association and measures of variance and discriminatory accuracy. The analyses are stratified by the existence of previous mental issues. Values are point estimations with (95% Confidence Intervals)

	Without metal health issues		With mental health issues	
	Model 1	Model 2	Model 1	Model 2
**Measures of association,** Odds Ratios				
Age				
12-17		Reference		Reference
18-23		1.73 (1.33-2.22)		1.52 (1.33-1.71)
24-30		1.90 (1.48-2.40)		2.58 (2.29-2.91)
Income				
High		Reference		Reference
Middle		1.16 (0.91-1.48)		0.89 (0.79-1.01)
Low		1.17 (0.92-1.55)		0.89 (0.78-1.01)
Immigrant background				
No		Reference		Reference
Yes		0.65 (0.53-0.81)		0.55 (0.49-0.61)
Hormonal contraceptives				
No		Reference		Reference
Yes		1.40 (1.12-1.71)		1.18 (1.06-1.34)
**Measures of variance**				
Variance*	0.224 (0.130-0.372)	0.077 (0.038-0.141)	0.287 (0.174-0.468)	0.017 (0.008-0.033)
VPC	6.38%	2.29%	8.02%	0.51%
PCV		65.67%		94.09%
AUC	0.61 (0.61-0.61)	0.61 (0.61-0.61)	0.64 (0.64-0.64)	0.64 (0.64-0.64)

*Between-strata variance, variance partition coefficient (VPC), proportional change of the variance (PCV), Area under the curve (AUC)

Zettermark S, *et al. BMJ Open* 2021; 11:e049553. doi: 10.1136/bmjopen-2021-049553

Table 3. Absolute risk (AR) of antidepressant use, and AR difference (ARD) between user and non-users of hormonal contraceptives but otherwise sharing the same intersectional stratum. The values are calculated from the multilevel analysis of individual heterogeneity and discriminatory accuracy (MAIHDA)

Previous mental health issues	Age (years)	Income level	Immigrant background	Number of women	Use of hormonal contraceptive		
					Yes AR	No AR	Yes-No difference ARD
No	12 – 17	Low	No	14060	4.4	1.8	2.7 (2 - 3.4)
			Yes	3123	1.5	0.8	0.8 (-0.1 - 2)
		Middle	No	37376	3.6	1.3	2.2 (1.8 - 2.6)
			Yes	4543	2.4	1.0	1.4 (0.3 - 2.8)
		High	No	62712	2.4	1.1	1.3 (1 - 1.5)
			Yes	2165	2.8	1.2	1.7 (0.4 - 3.3)
	18 – 23	Low	No	25939	4.2	3.4	0.8 (0.3 - 1.2)
			Yes	5720	2.5	2.0	0.5 (-0.3 - 1.4)
		Middle	No	40241	3.3	3.6	-0.3 (-0.6 - 0.1)
			Yes	4547	2.7	1.8	0.9 (0 - 1.9)
		High	No	74281	2.7	2.9	-0.2 (-0.4 - 0.1)
			Yes	2207	2.0	2.7	-0.6 (-1.9 - 0.5)
	24 – 30	Low	No	83448	3.6	3.7	0 (-0.3 - 0.2)
			Yes	21013	2.9	2.1	0.8 (0.3 - 1.4)
		Middle	No	31818	4.0	3.5	0.5 (0.1 - 0.9)
			Yes	7826	3.0	2.7	0.3 (-0.5 - 1.2)
		High	No	25335	2.9	3.0	-0.1 (-0.5 - 0.3)
			Yes	2152	2.5	2.7	-0.2 (-1.5 - 1.2)
Yes	12 – 17	Low	No	3371	30.1	22.3	7.8 (4.7 - 10.9)
			Yes	429	20.4	13.4	7 (-0.4 - 14.8)
		Middle	No	6787	31.0	23.1	7.9 (5.7 - 10.1)
			Yes	565	19.5	14.3	5.2 (-1.5 - 12.7)
		High	No	7807	33.8	27.7	6.1 (3.9 - 8.2)
			Yes	369	29.7	19.4	10.3 (1.2 - 19.6)
	18 – 23	Low	No	10205	38.8	36.9	1.9 (0.1 - 3.8)
			Yes	1068	28.1	19.0	9.1 (3.5 - 14.6)
		Middle	No	12082	36.6	35.0	1.6 (-0.1 - 3.3)
			Yes	805	27.2	19.1	8.1 (2 - 14.3)
		High	No	15994	37.2	38.5	-1.3 (-2.8 - 0.3)
			Yes	598	26.3	25.4	0.8 (-6.1 - 7.7)
	24 – 30	Low	No	26185	49.2	48.9	0.3 (-0.9 - 1.6)
			Yes	3759	36.0	32.6	3.4 (-0.3 - 7)
		Middle	No	7820	49.2	50.4	-1.3 (-3.6 - 1)
			Yes	1130	31.4	37.2	-5.8 (-12.4 - 0.9)

High	No	5868	47.8	47.8	0 (-2.6 - 2.6)
	Yes	441	41.3	35.2	6.1 (-3.6 - 16)
