# Supplementary material for: Population heterogeneity in associations between hormonal contraception and antidepressant use in Sweden: a prospective cohort study applying intersectional multilevel analysis of individual heterogeneity and discriminatory accuracy (MAIHDA)
Source: BMJ Open. 2021 Oct 1;11(10):e049553. doi: 10.1136/bmjopen-2021-049553 (PMC8488727; doi:10.1136/bmjopen-2021-049553)
Supplement: Author's manuscript [file bmjopen-2021-049553.draft_revisions.pdf]

BMJ Open is committed to open peer review. As part of this commitment we make the peer review history of every article we publish publicly available.

When an article is published we post the peer reviewers' comments and the authors' responses online. We also post the versions of the paper that were used during peer review. These are the versions that the peer review comments apply to.

The versions of the paper that follow are the versions that were submitted during the peer review process. They are not the versions of record or the final published versions. They should not be cited or distributed as the published version of this manuscript.

BMJ Open is an open access journal and the full, final, typeset and author-corrected version of record of the manuscript is available on our site with no access controls, subscription charges or pay-per-view fees (http://bmjopen.bmj.com).

If you have any questions on BMJ Open's open peer review process please email info.bmjopen@bmj.com

BMJ Open

Hormonal Contraception and Anti-depressant Use in Sweden: An Intersectional Multilevel Analysis of Individual Heterogeneity and Discriminatory Accuracy (MAIHDA)

Journal:	*BMJ Open*
Manuscript ID	bmjopen-2021-049553
Article Type:	Original research
Date Submitted by the Author:	03-Feb-2021

Complete List of Authors:	Zettermark, Sofia; Lund University, Unit for Social Epidemiology, Department of Clinical Sciences, Malmö<br>Khalaf, Kani; Lund University, Unit for Social Epidemiology, Department of Clinical Sciences, Malmö<br>Perez-Vicente, Raquel; Lund University, Unit for Social Epidemiology, Department of Clinical Sciences, Malmö<br>Leckie, George; University of Bristol, Center for Multilevel Modelling, School of Education; Lund University, Unit for Social Epidemiology, Department of Clinical Sciences<br>Mulinari, Diana; Lund University, Department of Gender Studies, Faculty of Social Sciences<br>Merlo, Juan; Lund University, Unit for Social Epidemiology, Department of Clinical Sciences; Region Skane Health Care, Center for Primary Health Care Research, Region Skåne

Keywords:	MENTAL HEALTH, Sex steroids & HRT < DIABETES & ENDOCRINOLOGY, SOCIAL MEDICINE, EPIDEMIOLOGY, Depression & mood disorders < PSYCHIATRY

SCHOLARONE™
Manuscripts

I, the Submitting Author has the right to grant and does grant on behalf of all authors of the Work (as defined in the below author licence), an exclusive licence and/or a non-exclusive licence for contributions from authors who are: i) UK Crown employees; ii) where BMJ has agreed a CC-BY licence shall apply, and/or iii) in accordance with the terms applicable for US Federal Government officers or employees acting as part of their official duties; on a worldwide, perpetual, irrevocable, royalty-free basis to BMJ Publishing Group Ltd ("BMJ") its licensees and where the relevant Journal is co-owned by BMJ to the co-owners of the Journal, to publish the Work in this journal and any other BMJ products and to exploit all rights, as set out in our licence.

The Submitting Author accepts and understands that any supply made under these terms is made by BMJ to the Submitting Author unless you are acting as an employee on behalf of your employer or a postgraduate student of an affiliated institution which is paying any applicable article publishing charge ("APC") for Open Access articles. Where the Submitting Author wishes to make the Work available on an Open Access basis (and intends to pay the relevant APC), the terms of reuse of such Open Access shall be governed by a Creative Commons licence – details of these licences and which Creative Commons licence will apply to this Work are set out in our licence referred to above.

Other than as permitted in any relevant BMJ Author's Self Archiving Policies, I confirm this Work has not been accepted for publication elsewhere, is not being considered for publication elsewhere and does not duplicate material already published. I confirm all authors consent to publication of this Work and authorise the granting of this licence.

For peer review only

Hormonal Contraception and Anti-depressant Use in Sweden: An Intersectional Multilevel Analysis of Individual Heterogeneity and Discriminatory Accuracy (MAIHDA)

Sofia Zettermark[1*], Kani Khalaf[1], Raquel Perez-Vicente[1], George Leckie[1,3], Diana Mulinari[2], Juan Merlo[1,4]

[1] Unit for Social Epidemiology, Department of Clinical Sciences, Faculty of Medicine, Lund University, Malmö, Sweden

[2] Department of Gender Studies, Faculty of Social Sciences, Lund University, Lund, Sweden

[3] Center for Multilevel Modelling, School of Education, University of Bristol, Bristol, United Kingdom

[4] Center for Primary Health Care Research, Region Skåne, Malmö, Sweden

*Corresponding author:
Sofia Zettermark
sofia.zettermark@med.lu.se
Unit for Social Epidemiology, Department of Clinical Sciences
Faculty of Medicine, Lund University
Jan Waldenströms gata 35, CRC
214 28, Malmö, Sweden

Word count: 3965

Keywords: Epidemiology, Contraception, Hormonal contraception, Mental health, Multilevel analysis

For peer review only - http://bmjopen.bmj.com/site/about/guidelines.xhtml

ABSTRACT

Objectives From a reproductive justice framework, we aimed to investigate how a possible association between hormonal contraceptive (HC) and anti-depressants use (as a proxy for depression) is distributed across intersectional strata in the population. We aimed to visualize how intersecting power dynamics may operate in combination with HC use to predispose for depression. Our main hypothesis was that the previously observed association between HC and anti-depressants use would vary between strata, being more pronounced in more oppressed intersectional contexts. For this purpose, we applied an intersectional Multilevel Analysis of Individual Heterogeneity and Discriminatory Accuracy (MAIHDA) approach.

Design Observational prospective cohort study using record linkage of national Swedish registers.

Setting The population of Sweden.

Participants All 978 761 women aged 12-30 residing in Sweden 2010, without a recent pregnancy and alive during one-year follow-up.

Primary and secondary outcome measures Use of any anti-depressant, meaning being dispensed at least one anti-depressant (ATC N06A) during follow-up.

Results Previously mentally healthy hormonal contraceptive users had an odds ratio of 1.79 for use anti-depressants compared to non-users, whereas this number was 1.28 for women with previous mental health issues. The highest absolute risks for anti-depressant use were uniformly found in strata with previous mental health issues, with highest risk in women aged 24-30 with no immigrant background, low income, and HC use (51.4%). The largest difference in anti-depressant use between HC users and non-users was found in teenagers, and in adult women of immigrant background with low income. Of the total individual variance in the latent propensity of using antidepressant 9.01% (healthy) and 8.16% (with previous mental health issues) was found at the intersectional stratum level.

Conclusions Our study suggests teenagers and women with immigrant background and low income could be more sensitive to mood effects of HC, a heterogeneity important to consider moving forward.

For peer review only - http://bmjopen.bmj.com/site/about/guidelines.xhtml

STRENGTHS AND LIMITATIONS OF THIS STUDY

- Entire Swedish population of women aged 12-30 included
- Pharmacy dispensing automatically linked to individual personal identification number in Sweden through the Swedish Prescribed Drug Register and thus very reliable
- Intersectional MAHIDA is a fruitful way of epidemiologically investigating heterogeneity within a population while considering individual conditions determined by societal power dimensions such as class, gender and race
- Anti-depressant dispensing is not a perfect proxy for depression
- Registers cannot not measure actual use of any medication

For peer review only

INTRODUCTION

In recent years, attention in the medical community has increasingly been drawn towards depression and other adverse effects on mood related to use of hormonal contraception (HC).(1, 2) Discontinuation rates are high, with mood disturbances or depression being one of the most common complaints.(3-5) Two large epidemiological studies, one in Denmark and the other performed in Sweden, have recently shown a higher risk of anti-depressants and psychotropic drugs use in women on HC, particularly in teenagers.(6, 7) Randomized controlled trials are rare, but suggest a negative influence of HC on well-being and sexual function,(8, 9) as well as evidence of HC modulating brain activity with subsequent mood alterations in some women.(10, 11) Even though oestrogen and progesterone are known to affect mood,(12) the growing body of evidence in this field is contradictory, with recent reviews concluding that both protective and negative effects of HC on mood exist and more research is needed.(13-16) Despite this uncertainty, many scholars agree that certain subgroups of women seem more vulnerable to psychological side effects of HC than others, particularly teenagers and women with previous mental health issues.(10, 13, 17-20) A call for further investigation into these vulnerable subgroups has been made.(14)

A fruitful way of epidemiologically investigating heterogeneity within a population while considering individual conditions determined by societal power dimensions such as class, gender and race has been developed through intersectional theory in recent years.(21-26) Intersectionality theory was first articulated by Black feminist scholars as a way of understanding how an individual inhabits and is formed by more than one social relation such as gender "race" or class, and how these classification systems interconnect to create specific contexts of oppression or privilege.(27, 28) These categorizations should not be seen as individual "risky" identities, but as the social, political and economic contextual conditions that outline our lives through structural inequalities.(29) Reproductive justice is a theoretical framework that builds upon intersectionality and centres diverse groups of unprivileged women's reproductive experiences to recognize that societal context and differing resources available shape reproductive health.(30) Applying a reproductive justice framework, it becomes clear that we need to take notice of disparate sociocultural contexts and interlocking power dimensions to understand different patterns of usage as well as possible diverse responses to HC.(31, 32)

To operationalize an intersectional mapping of heterogeneity in use of anti-depressants in relation to HC on a population level, we used a multilevel analysis of individual heterogeneity and discriminatory accuracy (MAIHDA).(21-23, 33, 34) We created

For peer review only - http://bmjopen.bmj.com/site/about/guidelines.xhtml

For peer review only

intersectional strata based on previous literature showing that age, socioeconomic position, and previous mental illness are relevant intersecting dimensions in understanding the relation between HC and depression.(17, 20, 35, 36)

We conceptualise the intersectional strata as social contexts rather than static individual traits, thereby visualising how intersecting power dynamics can act in combination with HC to predispose for depressive mood. Our main hypothesis was that the previously observed association between HC and use of anti-depressants would vary between strata and that this association would be more pronounced in more oppressed intersectional contexts. We investigate this hypothesis on the whole population of women susceptible to HC use in Sweden.

METHOD

Databases and study population

After allowance from the Swedish Ethical Authority and the data safety committees from Statistics Sweden and the Swedish National Board of Health and Welfare, we obtained a database created by record linkage of several nationwide registers administered by Statistics Sweden (the Swedish Population Register and the Longitudinal Integration Database for Health Insurance and Labour Market Studies, LISA) and the Swedish National Board of Health and Welfare (National Patient Register, the Swedish Prescribed Drug Register (SPDR) and the Cause of Death Register). The Swedish authorities linked the registries using a unique personal identification number, but the database was anonymized before delivering it to us. We defined an initial cohort containing all 1 064 171 women aged 12 - 30 years residing in Sweden 1st January 2010 and obtained individual level data on medication use from SPDR, which contain all dispensed drug prescriptions at Swedish pharmacies since 2006.

Every woman was assigned an individual baseline date, defined by the first dispensed prescription of an HC drug between 1 January 2010 and 31 December 2014 after 12 years of age. If a woman did not fill an HC prescription during this period, she was assigned a date midmost her own baseline time, i.e. July 2012 for adults, but later for the youngest girls. From the individual baseline date, the women were followed for one year to find out if a prescription of an antidepressant was dispensed. Data was also collected on psychiatric disorders and psychotropic drug use in the past three years (se Assessment of variables). After excluding women with incomplete follow-up time due to death, emigration, missing information on country of birth, and pregnancies one year before and after the

For peer review only - http://bmjopen.bmj.com/site/about/guidelines.xhtml

baseline, the final database consisted of 978 761 women. This database was divided into two cohorts according to the presence or absence of previous mental health issues, see Figure 1.

Assessment of variables

Users of HC were defined as any women who, according the SPDR, filled a prescription of HC (Anatomical Therapeutical Chemical (ATC) classification system codes G02B, G03AA-C) between 1 January 2010 and 31 December 2014, while non-users did not have a prescription filled during the same period. Most prescriptions of HC are acquired via a midwife in Sweden, although physicians also can prescribe, and the prescriptions can be dispensed by pharmacies annually or every three months.

Anti-depressant use, the outcome of our study, was defined, according to the SPDR, as being dispensed at least one prescription of antidepressants (ATC: N06A) during the one-year follow-up.

Previous mental health issues were defined as having any psychiatric disorder (ICD: F00-F99) or psychotropic drug use (ATC: N05A, N05B, N06A) in the past three years.

Pregnancies one year previous to baseline and during follow-up were identified according to the 2019 version of the Nordic Diagnosis-Related Group classification (NordDRG), Major Diagnostic Categories codes M14 for pregnancy, delivery and post-partum care.(37)

We used family level data on income as of 31 December 2010 from Statistics Sweden's LISA. Individualized disposable family income was calculated by dividing the total disposable income of the family by the number of family members, taking into account the different consumption weights of adults and children determined by Statistics Sweden. Thereafter, we created three categories (i.e., low, medium, and high) of income using tertile cut-offs based on the total Swedish population aged 18 - 80 years. We considered the high-income category as the reference in the comparisons.

We defined immigrant status at the family level as no family member >18 years of age born in Sweden, since understanding of and access to institutions such as health care differ depending on social position such as it is constructed by the power dimensions of race/immigration, as well as the experience of xenophobia. This variable should therefore be considered as an effort to capture a social position affecting possibilities and life trajectories rather than an essentialist view of otherness.

For peer review only - http://bmjopen.bmj.com/site/about/guidelines.xhtml

We categorized age at the individual baseline into the following groups: 12 to 17, 18 to 23, and 24 to 30 years to capture age specific conditions of adolescents, young adults, and adult women.

Intersectional Strata

We generated 36 intersectional strata within each cohort stratified by previous mental health issues, by combining three categories of age, three categories of income, two categories of immigrant background, and finally two categories of HC use. Mental health issues can be considered as a valid category of intersectional investigation in a society that consider an able body and mind vital, in other words relating to the power dimension of able-bodiedness,(38, 39) but was also included in the analysis since it is a strong determinant of antidepressant use that needs to be addressed. We could consider that over and above individual characteristics, mental illness-related stigma may condition inequities in health care.(40) As with gender or income, able-bodiedness concerning mental health can therefore be conceptualized as a contextual dimension when defining intersectional strata.

Statistical analysis

We performed an intersectional MAIHDA with individual women at the first level and the 36 intersectional strata at the second level stratified by previous mental health issues, see Supplementary material 1-4. The risk of antidepressant use was thus analysed through two successive multilevel logistic regression models distinguishing between measures of association and measures of variance and discriminatory accuracy.

Model 1

The first model included only an intercept and a random effect for the intersectional strata with no covariates. In this model 1 we first (i) performed a simple analysis of components of variance and calculated the Variance Partition Coefficient (VPC). That is, the share (expressed as a percentage) of the total individual variance in the latent propensity of antidepressant use that is at the intersectional strata level. In this simple model, the VPC correspond with the Intraclass Correlation Coefficient (ICC) which informs on the clustering of antidepressant use within intersectional strata. The VPC values extend from 0 to 100%. Second, (ii) we calculate the stratum-specific absolute risks (AR) and their 95% credible intervals (CI) by transformation of the information from the logistic regression to the probability scale. We used this information to map the AR heterogeneity across the

For peer review only - http://bmjopen.bmj.com/site/about/guidelines.xhtml

intersectional strata. Then, (iii) using these stratum-specific predictions, we calculated the Area Under the receiver operator characteristics Curve (AUC). The AUC informs on the accuracy of the intersectional strata information for discriminating those women who used antidepressants from those who did not. The AUC values extend from 0.5 to 1, where 1 represents total accuracy and 0.5 represent absence of accuracy. Both the VPC and the AUC in model 1 can be interpreted as measures of discriminatory accuracy,(41) and inform on the magnitude of the general intersectional effects. The higher the VPC and AUC values are, the higher the influence of the intersectional context on the individual use of antidepressants. Finally, (iv) we calculated the AR difference (ARD) and 95% CI between similar pairs of strata differing only on the use of HC. This ARD represents the stratum specific association between HC and antidepressant use.

Model 2 or fixed main effects model

This model includes the fixed, main effects of all the intersectional dimensions (i.e., age, income, immigrant background and HC use) used to define the intersectional strata. In model 2 we quantified, (i) the association between the intersectional dimensions and the risk of antidepressant use as expressed by the odds ratio (OR) and 95% CI. We also to calculate (ii) the Proportional Change in the Variance (PCV). The PCV measures the overall proportion of strata variance of model 1 explained by the specific intersectional dimensions. Since model 2 contains all the variables used to construct the intersectional strata as main effects, it should explain all the strata variance (i.e., PCV= 100%). If this is not the case, the remaining between strata variance would be due to the existence of multiplicative interaction of effects between the intersectional dimensions defining the strata.(22, 42)

The AUCs of the models 1 and 2 are expected to be the same because model 2 only decomposes the stratum-specific predicted probabilities obtained in model 1 into fixed and random effect components and their sum equals the prediction obtained only by random effects in model 1.

We ran the models using MLwiN 3.00 by calling it from within Stata 14.1 using the *runmlwin* command.(43) The estimations were performed using Markov chain Monte Carlo (MCMC) methods. All points estimations and their 95% credible intervals were based on the parameter and random effect chains obtained from the MCMC estimation. See elsewhere for further information on the statistical MAIHDA analysis including Stata commands,(33, 42) and discussion on the theory and methodological approach.(22, 44)

For peer review only - http://bmjopen.bmj.com/site/about/guidelines.xhtml

Patient and Public Involvement statement

The research was developed with a grassroot perspective in mind, whereby women's experiences of use of hormonal contraception inspired and informed the choice of research area and research questions. The anonymised data and scope of the study, including around 1 million women, prohibited direct patent involvement.

RESULTS

Characteristics of the population

As show in Figure 1, the study population consisted of 978 761 women aged 12 – 30. Out of those 13.0% (n = 127 323) had previous mental health issues. Mean age was somewhat older for women with previous mental health issues (22.6 years; SD 4.8) than for those without such concerns (20.9 years; SD 5.3). Supplementary table 5 shows pooled statistics for usage of previous mental health issues and HC use. Table 1 displays the baseline characteristics of the population by previous mental health issues and use of hormonal contraceptives.

Table 1. Characteristics of the 978 761 women aged 12 - 30 years and residing in Sweden by 1st January 2013 by previous mental health issues and use of hormonal contraceptives. Values are percentages (number of women) if not otherwise indicated.

	Previous mental health issues			
	No 87.0 (n= 851 438)		Yes 13.0 (n= 127 323)	
	Use of HC		Use of HC	
	Yes 45.4 (n= 386 492)	No 54.6 (n= 464 946)	Yes 48.6 (n= 61 914)	No 51.4 (n= 65 409)
Anti-depressant use	3.0	1.9	42.8	39.8
Age				
12-17 years	16.8	42.1	13.7	19.4
18-23 years	48.4	23.3	45.8	31.2
24-30 years	34.8	34.6	40.3	49.4
Income level				
Low	32.5	33.1	41.7	45.6
Middle	25.3	29.5	26.4	27.5
High	42.4	37.4	31.9	27.0
Immigrant background				
No	93.7	82.6	89.1	93.9
Yes	6.3	17.4	10.9	6.1

For peer review only - http://bmjopen.bmj.com/site/about/guidelines.xhtml

Among healthy women, 45.4% (n = 386 942) were users of HC, while this share was 48.6% (n = 61 914) for women with previous mental health issues. Anti-depressants were dispensed to 3.0% of HC users compared to 1.9% of non-users among healthy women during follow-up. For women with previous mental health issues, 42.8% of HC users and 39.8% of non-users were dispensed an anti-depressant. The income levels were generally higher among women without mental health issues, but the differences between HC users and non-users within each cohort were small. Women with immigrant background were less likely use HC (6.3%) if they were previously healthy than if they had pre-existing mental issues (10.9%), while the opposite was true for women without such background.

Results from the MAIHDA

Table 2 shows the results from the MAIHDA distinguishing between measures of association and measures of variance and discriminatory accuracy.

Table 2. Results from the Multilevel Analysis of Individual Heterogeneity and Discriminatory Accuracy (MAIHDA) distinguishing between measures of association and measures of variance and discriminatory accuracy. The analyses are stratified by the existence of previous mental issues. Values are odds ratios with (95% Confidence Intervals)

	Without metal health issues		With mental health issues	
	Model 1	Model 2	Model 1	Model 2
Age				
12-17		1.00		1.00
18-23		1.71 (1.28-2.19)		1.52 (1.33-1.73)
24-30		2.02 (1.54-2.68)		2.62 (2.28-3.07)
Income				
High		1.00		1.00
Middle		1.12 (0.94-1.40)		0.88 (0.76-0.99)
Low		1.06 (0.84-1.38)		0.88 (0.79-0.99)
Immigrant background				
No		1.00		1.00
Yes		0.64 (0.50-0.81)		0.57 (0.51-0.63)
Hormonal contraceptives				
No		1.00		1.00
Yes		1.79 (1.41-2.21)		1.28 (1.16-1.42)
**Measures of variance**				
Variance*	0.33 (0.19-0.53)	0.11 (0.06-0.18)	0.29 (0.18-0.48)	0.02 (0.01-0.04)

For peer review only - http://bmjopen.bmj.com/site/about/guidelines.xhtml

VPC	9.01%	3.09%	8.16%	0.55%
PCV		67.78%		93.84%
AUC	0.62 (0.62-0.62)	0.62 (0.62-0.62)	0.64 (0.64-0.640)	0.64 (0.64-0.640)

*Between-strata variance, variance partition coefficient (VPC), proportional change of the variance (PCV), Area under the curve (AUC)

Model 1 indicates that 9.01% (without mental health issues) and 8.16% (with previous mental health issues) of the total individual variance in the latent propensity of using antidepressant is at the intersectional strata level. These VPCs correspond with AUC values of 0.62 and 0.64 respectively. Both measures suggest the existence of a moderate intersectional effect, largely driven by the main effects of the covariates. The PCV was considerably higher in the group with previous mental health issues, meaning the intersectional dimensions or main effects explain more of the inter-strata variance. Model 2 shows that HC was associated with increased risk of antidepressant use after adjustment for all other intersectional dimensions. This result was seen within both cohorts, but more strongly so in women without previous mental health issues (OR 1.79 compared to 1.28). Finally, the VPC in model 2 was very small (3.09% and 0.55% respectively) but did not vanish. This finding means that while the intersectional strata effect was mainly due the additive effect of variables defining the strata, a small component due to interaction of effects could also be detected.

Heterogeneity concerning absolute risk of antidepressant use

Women with previous mental health issues presented much higher risk of antidepressant use than women without such issues, but the risk nonetheless varied across the other intersectional dimensions. Table 3 show the stratum-specific ARs or incidence rates for antidepressant use and 95% CI obtained in model 1.

Table 3. Absolute risk (AR) of antidepressant use, and AR difference (ARD) between user and non-users of hormonal contraceptives but otherwise sharing the same intersectional stratum. The values are calculated from the multilevel analysis of individual heterogeneity and discriminatory accuracy (MAIHDA)

Mental health issues	Age (years)	Income level	Immigrant background	Number of women	Use of hormonal contraceptive		
					Yes	No	Yes-No difference
					AR	AR	ARD

No	12— 17	Low	No	29 274	3.8	1.3	2.5 (2.1 — 3.0)
			Yes	7 776	1.2	0.5	0.7 (0.1 — 1.4)
		Middle	No	78 405	3.2	1.0	2.1 (1.9 — 2.4)
			Yes	10 291	2.0	0.6	1.4 (0.7 — 2.3)
		High	No	130 331	2.2	0.9	1.3 (1.1 — 1.5)
			Yes	4 745	2.9	0.8	2.0 (1.0 — 3.3)
	18 – 23	Low	No	47 678	3.7	3.0	0.7 (0.4 — 1.1)
			Yes	11 691	2.5	1.2	1.3 (0.8 — 1.9)
		Middle	No	76 451	3.0	2.8	0.2 (0.0 — 0.5)
			Yes	9 173	2.2	1.2	1.0 (0.5 — 1.7)
		High	No	145 735	2.4	2.3	0.1 (-0.1— 0.3)
			Yes	4 707	2.4	1.8	0.5 (-0.2— 1.3)
	24 – 30	Low	No	141 795	3.4	3.2	0.2 (0.0 — 0.4)
			Yes	41 436	3.1	1.4	1.8 (1.4 — 2.1)
		Middle	No	48 389	3.9	3.0	0.8 (0.5 — 1.2)
			Yes	11 649	2.9	2.4	0.5 (-0.1 — 1.2)
		High	No	47 985	2.7	2.6	0.1 (-0.2 — 0.4)
			Yes	3 927	2.1	2.3	0.2 (-1.1 — 0.8)
Yes	12— 17	Low	No	3 693	31.2	22.7	8.6 (5.7 — 11.4)
			Yes	458	24.8	13.6	11.2 (3.8 — 19.0)
		Middle	No	7 427	32.8	23.4	9.4 (7.3 — 11.4)
			Yes	603	21.8	14.3	7.5 (0.9 — 14.6)
		High	No	8 612	34.5	28.1	6.4 (4.4 — 8.4)
			Yes	406	30.8	19.8	11.0 (2.8 — 19.7)
	18 – 23	Low	No	12 165	40.3	37.8	2.5 (0.8 — 4.3)
			Yes	1 236	30.9	19.6	11.3 (6.3 — 16.2)
		Middle	No	14 301	38.6	36.3	2.3 (0.7 — 3.9)
			Yes	924	27.2	19.7	7.4 (2.0 — 12.9)
		High	No	19 372	39.4	39.8	-0.4 (-1.8 — 1.1)
			Yes	709	29.0	25.4	3.6 (-2.8 — 10.1)
	24— 30	Low	No	33 409	51.7	49.9	1.8 (0.7 — 2.9)
			Yes	4 634	40.8	32.4	8.4 (5.5 — 11.2)
		Middle	No	9 702	51.4	50.8	0.6 (-1.4 — 2.6)
			Yes	1 361	38.1	37.1	1.0 (-4.6 — 6.6)
		High	No	7 737	49.5	48.9	0.6 (-1.7 — 2.7)
			Yes	574	44.9	37.5	7.4 (-0.6 — 15.7)

The highest ARs were observed in non-immigrant women, aged 24-30, with previous mental health issues, using HC and with low (i.e., AR= 51.7%) as well as with middle-income (i.e., AR=51.4%). The lowest ARs were found in teenagers without previous mental health issues and no HC use, especially in the strata of immigrant girls from low (AR= 0.50%) and middle-income (AR= 0.60%) households.

For peer review only

Heterogeneity concerning the association between hormonal contraceptive and antidepressant use

Overall, the ARD between users and non-users of HC was highest in younger women between 12 and 17 years of age both with and without previous mental health issues, but this association was still considerable across nearly all strata. Table 3 gives detailed information on these associations. In the group of women without previous mental health issues the ARs are of low magnitude but the ARD show an increase risk of antidepressant among adolescent HC users (ARDs ranging from 0.7 to 2.5 percentage points) and adult women with immigrant background and low income (ARD 1.8 in the oldest age group). In the group of women with previous mental health issues the ARs were of much higher magnitude than in women without previous mental health issues. The ARDs were also larger, especially in teenage girls (ARD ranging from 6.4 to 11.2 percentage points) and again among women with immigrant background and low income (ARD 11.3 in ages 18 to 23), indicating a strong association between HC and antidepressant use. However, the 95% credible intervals were broad since the number of individuals was relatively small in those strata. The association between HC and antidepressant use was smaller in adult women native to Sweden no matter their income, and completely disappeared in adult women with high income regardless of immigrant background.

DISCUSSION

The main hypothesis of our study was that the previously observed association between HC and antidepressant use,(6, 7) would be modified by the intersectional context of the women, being more pronounced in more oppressed intersectional contexts. Our study replicates previous findings as we found the strongest associations between HC and antidepressant use in teenagers (6, 7). We also confirmed our hypothesis that the ARD was heterogenous across intersectional strata pairs as the ARD varied from 0.7 in low-income teenagers with immigrant background and without previous mental health issues to 11.3 in young women with previous mental health issues and immigrant background. As hypothesized, the ARD was more pronounced in more oppressed intersectional contexts like those composed by immigrant, low-income women with previous mental issues. That is, the AR and some extent ARDs varied mainly depending on previous mental health issues, but the HC-antidepressant association considerably modified across pair of strata with discrepant HC use in both cohorts.

For peer review only - http://bmjopen.bmj.com/site/about/guidelines.xhtml

Independently of previous mental health issues, the propensity for using anti-depressants was consistently higher for HC users than for non-users in teenagers aged 12-17, a result aligned with previous studies.(6, 7, 17, 18) As discussed in a previous paper, this higher risk could be due to a *selective discontinuation bias.*(7) A heterogeneous response to HC has been confirmed,(13, 20, 45, 46) where the women who experience a negative influence of HC on psychological health might discontinue treatment in early ages, while those without symptoms continued on HC into adulthood, creating this age disparity. Aside from adolescent girls, low and middle income adult women with immigrant background had a higher ARD, while adolescent girls with immigrant background had both the lowest ARs and a low grade of modification by HC use.

As expected, among adult women the overall propensity for using anti-depressants was higher, as it is known that anti-depressant use increases by age,(47, 48) and the difference between HC users and non-users was smaller. Women native to Sweden had a higher absolute risk of using anti-depressants, but this was moderated by HC exposure to a lower extent than for immigrant women. In adult women native to Sweden, HC use gave no risk increase of antidepressant use among those with high income. The lower absolute risk does not necessarily mean that immigrant women are healthier, since earlier studies have found immigrants utilize healthcare to a lesser extent, even though the need is pronounced, because of discrimination.(49, 50)

The big difference in anti-depressant consumption depending on HC use for lower income immigrant women could be interpreted as the intersectional contexts embodied by these women are more susceptible to the potential detrimental effect of HC on mood. The interrelating negative consequences of low income as a proxy for class or social position, gender and xenophobia may accumulate over the life course and lead to a higher vulnerability to exposures that predispose for antidepressant use later in life,(51-53) whereas this diverse vulnerability to HC exposure might not be visible in teenagers. Social experiences can vary depending on for example social position, which in turn impact psychological development, mood and cognition, thus influencing health.(54, 55) In understanding how HC can impact women's mental health differently, both possible individual biological predispositions and social settings need to be investigated, since the emotional response to HC is influenced by context.(32) In other words, the interlocking power axes that create oppression could predispose women already under structural burdens for adverse mental health reactions when using HC. The fact that adult women native to Sweden were almost unaffected by HC use,

For peer review only - http://bmjopen.bmj.com/site/about/guidelines.xhtml

could strengthen this suggestion. Without the intersectional strata this disparity would not have been so easily identified and visualized.

Focusing on women whose lives are affected by several interlocking power dimensions such as low social position and xenophobia is fundamental to achieving reproductive justice.(30) Nonetheless, our intersectional strata should not be considered static categories of inherently "risky identities" but must be interpreted as context specific vulnerabilities of women within certain interlocking positions, constituted in relation to power dynamics created by unequal schemes such as the economic system.(25, 29) It is likely that in other contexts, other groups could be more vulnerable. In identifying the underlying power systems creating these intersectional categories and acknowledging their constant movement and changing dynamics on a societal level, it furthermore becomes possible to address these inequalities through social change.

In this study, we have combined a classical epidemiological approach of exposure to HC and an intersectional MAHIDA to create a novel understanding of how intersecting power dynamics could create particular vulnerabilities to this specific exposure. Because of our study design where women are followed for one year after a dispensed prescription of HC, it is more theoretically coherent to view use of HC as an exposure rather than a component of the intersectional strata. However, it is possible to within our approach view HC use as a socio-contextual factor that captures certain living conditions (for example more likely to be sexually active or in a heterosexual relationship), which somewhat changes the interpretation of the results. This epistemological tension is not necessarily a limitation, but could enrich the dialogue in social epidemiology on whether it is possible to separate contextual factors from "pure" exposure.(56-58)

Limitations

The findings from this study must be interpreted in the context of its limitations. The SPDR has highly reliable data on dispensed prescriptions but cannot measure the actual use of dispensed medications. Use of anti-depressants can be considered a proxy for depression, but anti-depressants are also prescribed for other reasons than depression, including generalized anxiety disorder, obsessive-compulsive disorder and panic disorder.(59) Therefore it is not a perfect proxy of depression but may be a more general indication of impaired mental health.(60) However, out of all women with potentially unfavorable mental health effects from HC, only a subset would have symptoms severe enough to get an anti-depressant prescription, leading instead to many missed cases.

For peer review only - http://bmjopen.bmj.com/site/about/guidelines.xhtml

As in any observational study, ours only allows for measurements of associations and cannot determine causation. Furthermore, apparently strong average associations do not necessarily convey a high discriminatory accuracy (see elsewhere for a short review and discussion)(61). Nevertheless, since our analysis yielded a moderate accuracy (i.e., AUC=0.6), the intersectional strata do matter for the propensity to use antidepressants. A consideration in every quantitative intersectional study is the basis for creating intersectional categories, since comprehensive information on background and lived experiences are lacking and the categories are created based on available but crude proxies such as income level. For example, in our study the group of women with immigrant background was very heterogenous, so we cannot exclude that the increased AR of antidepressant use is located on more specific country of birth categories. There is an ongoing debate whether these crude categorizations are feasible, and extra caution should be taken when investigating emerging intersectional categories rather than established ones.(62)

Conclusion

It is important to recognise intersectional perspectives and interacting axes of oppression to tailor better public health interventions, as well as recognising the experiences of oppressed women to reach reproductive and social justice. (29, 63) Our intersectional MAIHDA methodology operationalizes this idea by providing information on the discriminatory accuracy of the contexts that define the intersectional strata. It highlights the need to consider disadvantages consisting of several interlocking structural dimensions such as income/class, age and immigration to better understand how HC might predispose certain women, mainly teenagers and low-income women with immigrant background, for depression. These vulnerabilities are based in inequalities that are not static, but structurally created and therefore possible to redeem.

Figure 1. Selection of the study population.

Acknowledgements

For peer review only - http://bmjopen.bmj.com/site/about/guidelines.xhtml

A previous version of this study was presented as a poster at the Gynecological Endocrinology, the 19th World Congress in December 2020. We thank all colleagues at the Unit for Social epidemiology, Lund university, for valuable discussions.

Ethical statement

The database was approved by the Regional Ethical Review Board in Lund, Sweden, the Data Safety Board at Statistics Sweden and the National Board of Health and Welfare (Dnr: 2014/ 856, 2015/341).

Funding statement

This work was supported by The Swedish Research Council (Vetenskapsrådet) grant number: [2017-01321] https://www.swecris.se/betasearch/details/project/201701321VR

Competing interests

None declared.

Data sharing statement

Public access to the data is restricted by the Swedish Authorities (Public Access to Information and Secrecy Act; http://www.government.se/information-material/2009/09/public-access-to-information-and-secrecy-act/) but data can be made available for researchers after a special review that includes approval of the research project by both an Ethics Committee and the authorities' data safety committees. The National Board of Health and Welfare is a government agency under the Ministry of Health and Social Affairs. It is not their policy to provide individual level data to researchers abroad. Instead, they normally advise researchers in other countries to cooperate with Swedish colleagues, to whom they can provide data according to standard legal provisions and procedures. Requests for access to the data can be made to the National Board of Health and Welfare and Statistics Sweden (http://www.socialstyrelsen.se/statistics; https://www.scb.se/en/services/guidance-for-researchers-and-universities/).

Contributorship statement

Sofia Zettermark: Conceptualization, design, analysis, interpretation of data, writing original draft, final approvement of version to be published.

For peer review only - http://bmjopen.bmj.com/site/about/guidelines.xhtml

Kani Kahlaf: Interpretation of data, revising draft critically for intellectual content, final approvement of version to be published.

Raquel Perez-Vicente: Design, analysis, interpretation of data, revising draft critically for intellectual content, final approvement of version to be published.

George Leckie: Analysis, interpretation of data, revising draft critically for intellectual content, final approvement of version to be published.

Diana Mulinari: Interpretation of data, revising draft critically for intellectual content, final approvement of version to be published.

Juan Merlo: Conceptualization, design, analysis, interpretation of data, revising draft critically for intellectual content, final approvement of version to be published.

REFERENCES

1 Bitzer J. Hormonal contraception and depression: another Pill scandal? *Eur J Contracept Repr* 2017;22(1):1-2.
2 Frokjaer VG. Pharmacological sex hormone manipulation as a risk model for depression. *J Neurosci Res* 2020;98(7):1283-92.
3 Rosenberg MJ, Waugh MS. Oral contraceptive discontinuation: a prospective evaluation of frequency and reasons. *Am J Obstet Gynecol* 1998;179(3;1):577-82.
4 Simmons RG, Sanders JN, Geist C, et al. Predictors of contraceptive switching and discontinuation within the first 6 months of use among Highly Effective Reversible Contraceptive Initiative Salt Lake study participants. *Am J Obstet Gynecol* 2019;220(4):376e1-e12.
5 Sanders SA, Graham CA, Bass JL, et al. A prospective study of the effects of oral contraceptives on sexuality and well-being and their relationship to discontinuation. *Contraception* 2001;64(1):51-8.
6 Skovlund CW, Morch LS, Kessing LV, et al. Association of Hormonal Contraception With Depression. *JAMA Psychiat* 2016;73(11):1154-62.
7 Zettermark S, Perez Vicente R, Merlo J. Hormonal contraception increases the risk of psychotropic drug use in adolescent girls but not in adults: A pharmacoepidemiological study on 800 000 Swedish women. *PLoS One* 2018;13(3):e0194773 doi:10.1371/journal.pone.0194773.
8 Zethraeus N, Dreber A, Ranehill E, et al. A first-choice combined oral contraceptive influences general well-being in healthy women: a double-blind, randomized, placebo-controlled trial. *Fertil Steril* 2017;107(5):1238-45.
9 Lundin C, Malmborg A, Slezak J, et al. Sexual function and combined oral contraceptives - a randomised, placebo-controlled trial. *Endocr Connect* 2018;7(11):1208-1216 doi:10.1530/EC-18-0384
10 Gingnell M, Engman J, Frick A, et al. Oral contraceptive use changes brain activity and mood in women with previous negative affect on the pill--a double-blinded, placebo-controlled randomized trial of a levonorgestrel-containing combined oral contraceptive. *Psychoneuroendocrino* 2013;38(7):1133-44.
11 Fisher PM, Larsen CB, Beliveau V et al. Pharmacologically Induced Sex Hormone Fluctuation Effects on Resting-State Functional Connectivity in a Risk Model for Depression: A Randomized Trial. *Neuropsychopharmacol* 2017;42(2):446-53.

For peer review only - http://bmjopen.bmj.com/site/about/guidelines.xhtml

12 Toffoletto S, Lanzenberger R, Gingnell M, et al. Emotional and cognitive functional imaging of estrogen and progesterone effects in the female human brain: A systematic review. *Psychoneuroendocrino* 2014;50:28-52.
13 Schaffir J, Worly BL, Gur TL. Combined hormonal contraception and its effects on mood: a critical review. *Eur J Contracept Repr* 2016;21(5):347-55.
14 Robakis T, Williams KE, Nutkiewicz L, et al. Hormonal Contraceptives and Mood: Review of the Literature and Implications for Future Research. *Curr Psychiat Rep* 2019;21(7):57. doi:10.1007/s11920-019-1034-z
15 Lewis CA, Kimmig AS, Zsido RG, et al. Effects of Hormonal Contraceptives on Mood: A Focus on Emotion Recognition and Reactivity, Reward Processing, and Stress Response. *Curr Psychiat Rep* 2019;21(11):115.
16 Worly BL, Gur TL, Schaffir J. The relationship between progestin hormonal contraception and depression: a systematic review. *Contraception* 2018;97(6):478-89.
17 de Wit AE, Booij SH, Giltay EJ, et al. Association of Use of Oral Contraceptives With Depressive Symptoms Among Adolescents and Young Women. *JAMA Psychiat* 2020;77(1):52-59 doi:10.1001/jamapsychiatry.2019.2838
18 Gregory ST, Hall K, Quast T, et al. Hormonal Contraception, depression, and Academic Performance among females attending college in the United States. *Psychiat Res* 2018;270:111-6.
19 Lundin C, Danielsson KG, Bixo M, et al. Combined oral contraceptive use is associated with both improvement and worsening of mood in the different phases of the treatment cycle - A double-blind, placebo-controlled randomized trial. *Psychoneuroendocrino* 2017;76:135-43.
20 Bengtsdotter H, Lundin C, Gemzell Danielsson K, Bixo M, Baumgart J, Marions L, et al. Ongoing or previous mental disorders predispose to adverse mood reporting during combined oral contraceptive use. *Eur J Contracept Repr* 2018;23(1):45-51.
21 Wemrell M, Mulinari S, Merlo J. An intersectional approach to multilevel analysis of individual heterogeneity (MAIH) and discriminatory accuracy. *Soc Sci Med* 2017;178:217-9.
22 Merlo J. Multilevel analysis of individual heterogeneity and discriminatory accuracy (MAIHDA) within an intersectional framework. *Soc Sci Med* 2018;203:74-80.
23 Evans CR, Williams DR, Onnela JP, et al. A multilevel approach to modeling health inequalities at the intersection of multiple social identities. *Soc Sci Med* 2018;203:64-73.
24 Bauer GR. Incorporating intersectionality theory into population health research methodology: Challenges and the potential to advance health equity. *Soc Sci Med* 2014;110:10–7.
25 McCall L. The complexity of intersectionlity. *Signs* 2005;30:1771-800.
26 Evans CR, Erickson N. Intersectionality and depression in adolescence and early adulthood: A MAIHDA analysis of the national longitudinal study of adolescent to adult health, 1995-2008. *Soc Sci Med* 2019;220:1-11.
27 Crenshaw K. Mapping the Margins: Intersectionality, Identity Politics, and Violence against Women of Color. *Stanford Law Rev* 1991;43(6):1241-99.
28 Cho S, Crenshaw K, McCall L. Toward a field of Interscetionality Studies: Theory, Applications and Praxis. *Signs* 2013;38(4):785-810.
29 Price K. It's Not Just About Abortion: Incorporating Intersectionality in Research About Women of Color and Reproduction. *Women Health Iss* 2011;21(3):55-7.
30 Ross LJ, Rickie S. Reproductive Justice: An Introduction. Oakland, CA: University of California Press 2017:1-360.
31 Dehlendorf C, Ruskin R, Grumbach K, et al. Recommendations for intrauterine contraception: a randomized trial of the effects of patients' race/ethnicity and socioeconomic status. *Am J Obstet Gynecol* 2010;203(4):319e1-8.

For peer review only - http://bmjopen.bmj.com/site/about/guidelines.xhtml

32 Montoya ER, Bos PA. How Oral Contraceptives Impact Social-Emotional Behavior and Brain Function. *Trends Cogn Sci* 2017;21(2):125-36.
33 Persmark A, Wemrell M, Zettermark S, et al. Precision public health: Mapping socioeconomic disparities in opioid dispensations at Swedish pharmacies by Multilevel Analysis of Individual Heterogeneity and Discriminatory Accuracy (MAIHDA). *PLoS One* 2019;14(8):e0220322 doi:10.1371/journal.pone.0220322
34 Hernandez-Yumar A, Wemrell M, Abasolo Alesson I, et al. Socioeconomic differences in body mass index in Spain: An intersectional multilevel analysis of individual heterogeneity and discriminatory accuracy. *PLoS One* 2018;13(12):e0208624.
35 Lorant V, Deliège D, Eaton W, et al. Socioeconomic inequalities in depression: a meta-analysis. *Am J Epidemiol* 2003;157(2):98-112.
36 Dehlendorf C, Rodriguez MI, Levy K, et al. Disparities in family planning. *Am J Obstet Gynecol* 2010;202(3):214-20.
37 Swedish National Board of Health and Welfare. DRG-koder och text 2019 [DRG codes and text 2019]. Available from: https://www.socialstyrelsen.se/utveckla-verksamhet/e-halsa/klassificering-och-koder/drg/drg-koder-och-definitioner/. [Accessed 2020 Dec 15]
38 Hankivsky O. Women's health, men's health, and gender and health: Implications of intersectionality. *Soc Sci Med* 2012;74(11):1712- 20.
39 Frederick A, Shrifrer D. Race and Disability: From Analogy to Intersectionality. *Sociology of Race and Ethnicity* 2019;5(2):200-14.
40 Knaak S, Mantler E, Szeto A. Mental illness-related stigma in healthcare: Barriers to access and care and evidence-based solutions. *Healthcare Management Forum* 2017;30(2):111-6.
41 Merlo J, Wagner P, Ghith N, et al. An Original Stepwise Multilevel Logistic Regression Analysis of Discriminatory Accuracy: The Case of Neighbourhoods and Health. *PLoS One* 2016;11(4):e0153778.
42 Axelsson Fisk S, Mulinari S, Wemrell M, et al. Chronic Obstructive Pulmonary Disease in Sweden: An intersectional multilevel analysis of individual heterogeneity and discriminatory accuracy. *SSM Popuulationl Health* 2018;4:334-46.
43 Leckie G, Charlton C. Runmlwin - a program to Run the MLwiN multilevel modelling software from within stata. *J Stat Softw* 2013;52(11):1-40.
44 Evans CR, Leckie G, Merlo J. Multilevel versus single-level regression for the analysis of multilevel information: The case of quantitative intersectional analysis. *Soc Sci Med* 2020;245:112499 doi:10.1016/j.socscimed.2019.112499.
45 Segebladh B, Borgström A, Odlind V, et al. Prevalence of psychiatric disorders and premenstrual dysphoric symptoms in patients with experience of adverse mood during treatment with combined oral contraceptives. *Contraception* 2009;79(1):50-5.
46 Borgström A, Odlind V, Ekselius L, et al. Adverse mood effects of combined oral contraceptives in relation to personality traits. *Eur J Obstet Gyn R B* 2008;141(2):127-30.
47 Carlsten A, Waern M, Ekedahl A, et al. Antidepressant medication and suicide in Sweden. *Pharmacoepidem Dr S* 2001;10(6):525-30.
48 Kalmar S, Szanto K, Rihmer Z, et al. Antidepressant prescription and suicide rates: effect of age and gender. *Suicide Life Threat* 2008;38(4):363-74.
49 Mangrio E, Carlson E, Zdravkovic S. Understanding experiences of the Swedish health care system from the perspective of newly arrived refugees. *BMC Research Notes* 2018;11(1):616.
50 Kirmayer LJ, Narasiah L, Munoz M, et al. Common mental health problems in immigrants and refugees: general approach in primary care. *Can Med Assoc J* 2011;183(12):E959-67.
51 Brenner H. Political economy and health. In: Amick III B, Levine S, Tarlov A, et al, eds. Society and health. New York, NY: Oxford university press 1995:211–46.

For peer review only - http://bmjopen.bmj.com/site/about/guidelines.xhtml

52 Lynch JW, Kaplan GA, Salonen JT. Why do poor people behave poorly? Variation in adult health behaviours and psychosocial characteristics by stages of the socioeconomic lifecourse. *Soc Sci Med* 1997;44(6):809-19.
53 Collins PY, von Unger H, Armbrister A. Church ladies, good girls, and locas: stigma and the intersection of gender, ethnicity, mental illness, and sexuality in relation to HIV risk. *Soc Sci Med* 2008;67(3):389-97.
54 Honjo K. Social epidemiology: Definition, history, and research examples. *Environ Health Prev* 2004;9(5):193-9.
55 Kubzansky L, Kawachi I. Affective States and Health. In: Berkman L, Kawachi I, eds. Social epidemiology. New York, NY: Oxford University Press 2000:13–41.
56 Krieger N. Epidemiology and the Peoples Health - Theory and Context. Oxford: Oxford University Press 2011:1-381.
57 Krieger N. Epidemiology and social sciences: towards a critical reengagement in the 21st century. *Epidemiol Rev* 2000;22(1):155-63.
58 Link B, Phelan J. Social conditions as fundamental causes of disease. *J Health Soc Behav* 1995(Extra Issue:Forty Years of Medical Sociology):80-94
59 Schatzberg AF. New indications for antidepressants. *J Clin Psychiat* 2000;61(Suppl)11:9-17.
60 Gardarsdottir H, Heerdink ER, van Dijk L, et al. Indications for antidepressant drug prescribing in general practice in the Netherlands. *J Affect Disorders* 2007;98(1-2):109-15.
61 Merlo J, Mulinari S, Wemrell M, et al. The tyranny of the averages and the indiscriminate use of risk factors in public health: The case of coronary heart disease. *SSM Population Health* 2017;3:684-98 doi:10.1016/j.ssmph.2017.08.005
62 Warner LR. A Best Practices Guide to Intersectional Approaches in Psychological Research. *Sex Roles* 2008;59:454–63.
63 Marmot M. Social justice, epidemiology and health inequalities. *Eur J Epidemiol* 2017;32(7):537-46.

For peer review only

For peer review only - http://bmjopen.bmj.com/site/about/guidelines.xhtml

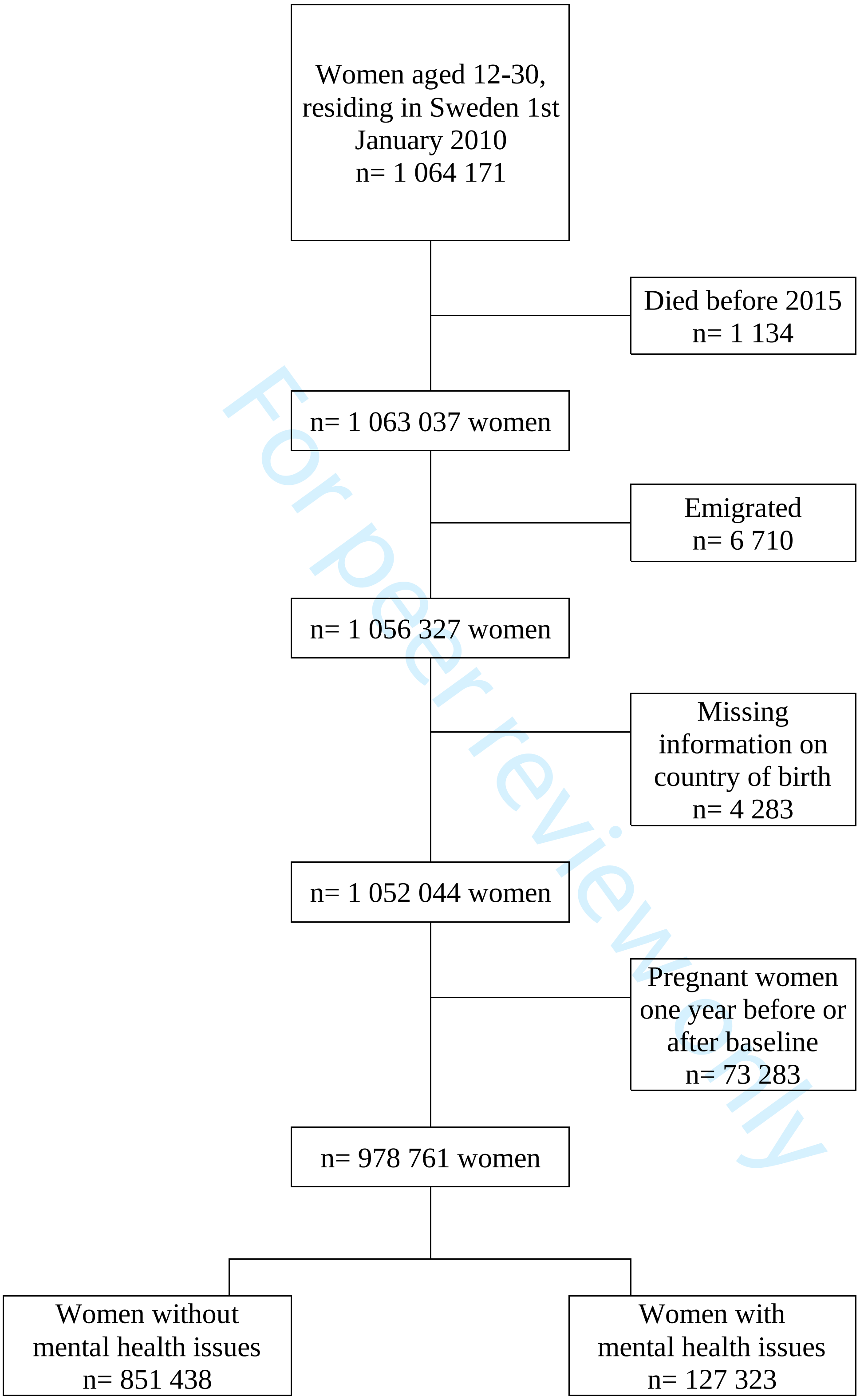

Figure 1. Selection of the study population.

For peer review only

For peer review only

```
* Hormonal Contraception and Antidepressant Use in Sweden: An
Intersectional Multilevel Analysis of Individual Heterogeneity and
Discriminatory Accuracy
* (MAIHDA)
**************************************************************************
clear *
global MLwiN_path "C:\Program Files\MLwiN v3.05\mlwin.exe"
set cformat %9.2f



**************************************************************************
* TABLE 1
**************************************************************************

* Load the data
use "final_mlMENTAL.dta", clear
keep age_cat1 age_cat2 age_cat3 inc1 inc2 inc3 imm pp proportion denom
order age_cat1 age_cat2 age_cat3 inc1 inc2 inc3 imm pp proportion denom

generate percentage = 100*proportion
drop proportion
format %9.2f percentage

generate age_cat = .
replace age_cat = 1 if age_cat1==1
replace age_cat = 2 if age_cat2==1
replace age_cat = 3 if age_cat3==1

generate inc_cat = .
replace inc_cat = 1 if inc1==1
replace inc_cat = 2 if inc2==1
replace inc_cat = 3 if inc3==1

* Results for the table
tabulate pp [fweight = denom]
table pp [fweight = denom], contents(mean percentage )
tabulate age_cat pp [fweight = denom], column nofreq
tabulate inc_cat pp [fweight = denom], column nofreq
tabulate imm pp [fweight = denom], column nofreq



**************************************************************************
* TABLE 2: MODEL 1
**************************************************************************

* Load the data
use "final_mlMENTAL.dta", clear

* IGLS estimation, for MCMC initial values
runmlwin prop cons, ///
  level2(inter: cons) ///
  level1(inter:) ///
  discrete(distribution(binomial) link(logit) denom(denom) mql1) ///
  nopause

* MCMC
runmlwin prop cons, ///
  level2(inter: cons, residuals(u, savechains("m1u.dta",replace))) ///
  level1(inter:) ///
```

```
  discrete(distribution(binomial) link(logit) denom(denom)) ///
  mcmc(burnin(10000) chain(50000) thin(10) savechains("m1b.dta", replace))
///
  initsprevious ///
  nopause

* Level-2 variance
scalar m1sigma2u = [RP2]var(cons)
scalar list m1sigma2u

* Level-1 variance
scalar m1sigma2e = _pi^2/3
scalar list m1sigma2e

* VPC
display "VPC_u = " %9.4f m1sigma2u/(m1sigma2u + m1sigma2e)

* Compress and save the data
compress
save "m1.dta", replace



*---------------------------------------------------------------------------*
* PREPARE FIXED-PART PAREMETER CHAINS
*---------------------------------------------------------------------------*

use "m1b.dta", clear
drop deviance RP2_var_cons_ OD_bcons_1
rename FP1_* b_*
format %9.2f b_*
compress
save "m1b_prepped.dta", replace
isid iteration
codebook iteration, compact



*----------------------------------------------------------------------------*
* PREPARE RANDOM EFFECTS CHAINS
*----------------------------------------------------------------------------*
use "m1u.dta", clear
drop residual idnum
rename value u
format %9.2f u
sort inter iteration
order inter iteration
compress
save "m1u_prepped.dta", replace
isid inter iteration
codebook iteration, compact



*----------------------------------------------------------------------------*
* MERGE DATA, FIXED-PART PARAMETER AND RANDOM EFFECT CHAINS TOGETHER
*----------------------------------------------------------------------------*
use "final_mlMENTAL", clear
count
cross using "m1b_prepped.dta"
count
```

```
merge m:1 inter iteration using "m1u_prepped.dta", nogenerate assert(match)
count
compress
save "m1data_prepped.dta", replace



*---------------------------------------------------------------------------*
* ROC
*---------------------------------------------------------------------------*
use "m1data_prepped.dta", clear
count
generate p = invlogit(b_cons + u)
gcollapse (mean) p, by(inter num denom)
count
expand denom
sort inter
bysort inter: generate y = (_n<=numerator)
generate prop = denom/_N
generate weight = int(1/prop)
roctab y p [fw=weight]



*---------------------------------------------------------------------------*
* TABLE 3
*---------------------------------------------------------------------------*
use "m1data_prepped.dta", clear
keep iteration inter age_cat1 age_cat2 age_cat3 inc1 inc2 inc3 imm pp denom
b_cons u
count
generate p = 100*invlogit(b_cons + u)
drop b_cons u
format %9.1f p
drop inter
reshape wide denom p, i(iteration age_cat1 age_cat2 age_cat3 inc1 inc2 inc3
imm) j(pp)
generate denom = denom0 + denom1
drop denom0 denom1
generate pdiff = p1 - p0
gcollapse (mean) p0 p1 pdiff (p2.5) pdifflo=pdiff (p97.5) pdiffhi=pdiff,
by(age_cat1 age_cat2 age_cat3 inc1 inc2 inc3 imm denom)
format %9.1f pdiff pdifflo pdiffhi
order p1 p0 pdiff pdifflo pdiffhi, last
gsort -age_cat1 -age_cat2 -age_cat3 -inc1 -inc2 -inc3 imm



****************************************************************************
* TABLE 2: MODEL 2:
****************************************************************************

* Load the data
use "final_mlMENTAL.dta", clear

* IGLS estimation, for MCMC initial values
runmlwin prop cons age_cat2 age_cat3 inc1 inc2 imm pp, ///
  level2(inter: cons) ///
  level1(inter:) ///
  discrete(distribution(binomial) link(logit) denom(denom) mql1) ///
  nopause
```

```
* MCMC
runmlwin prop cons age_cat2 age_cat3 inc1 inc2 imm  pp, ///
  level2(inter: cons, residuals(u,savechains("m2u.dta",replace))) ///
  level1(inter:) ///
  discrete(distribution(binomial) link(logit) denom(denom)) ///
  mcmc(burnin(10000) chain(50000) thin(10) savechains("m2b.dta", replace))
///
  initsprevious ///
  nopause

* Odds ratios
runmlwin, or

* Level-2 variance
scalar m2sigma2u = [RP2]var(cons)
scalar list m2sigma2u

* Level-1 variance
scalar m2sigma2e = _pi^2/3
scalar list m2sigma2e

* VPC
display "VPC_u = " %9.4f m2sigma2u/(m2sigma2u + m2sigma2e)

* Compress and save the data
compress
save "m2.dta", replace

* PCV
display "PCV = " %9.4f (m2sigma2u - m1sigma2u)/m1sigma2u



*---------------------------------------------------------------------------*
* PREPARE FIXED-PART PAREMETER CHAINS
*---------------------------------------------------------------------------*
use "m2b.dta", clear
drop deviance RP2_var_cons_ OD_bcons_1
rename FP1_* b_*
format %9.2f b_*
compress
save "m2b_prepped.dta", replace
isid iteration
codebook iteration, compact



*---------------------------------------------------------------------------*
* PREPARE inter RANDOM EFFECTS CHAINS
*---------------------------------------------------------------------------*
use "m2u.dta", clear
drop residual idnum
rename value u
format %9.2f u
sort inter iteration
order inter iteration
compress
save "m2u_prepped.dta", replace
isid inter iteration
codebook iteration, compact
```

```
*----------------------------------------------------------------------------*
* MERGE DATA, FIXED-PART PARAMETER AND RANDOM EFFECT CHAINS TOGETHER
*----------------------------------------------------------------------------*
use "final_mlMENTAL", clear
count
cross using "m2b_prepped.dta"
count
merge m:1 inter iteration using "m2u_prepped.dta"
count
save "m2data_prepped.dta", replace



*----------------------------------------------------------------------------*
* ROC
*----------------------------------------------------------------------------*
use "m2data_prepped.dta", clear
count
generate p = invlogit(b_cons + b_age_cat2*age_cat2 + b_age_cat3*age_cat3 +
b_inc1*inc1 + b_inc2*inc2 + b_imm*imm + b_pp*pp)
gcollapse (mean) p, by(inter num denom)
count
expand denom
sort inter
bysort inter: generate y = (_n<=numerator)
generate prop = denom/_N
generate weight = int(1/prop)
roctab y p [fw=weight]



*----------------------------------------------------------------------------*
* TABLE 3
*----------------------------------------------------------------------------*
use "m1data_prepped.dta", clear
keep iteration inter age_cat1 age_cat2 age_cat3 inc1 inc2 inc3 imm pp denom
b_cons u
count
generate p = 100*invlogit(b_cons + u)
drop b_cons u
format %9.1f p
drop inter
reshape wide denom p, i(iteration age_cat1 age_cat2 age_cat3 inc1 inc2 inc3
imm) j(pp)
generate denom = denom0 + denom1
drop denom0 denom1
generate pdiff = p1 - p0
gcollapse (mean) p0 p1 pdiff (p2.5) pdifflo=pdiff (p97.5) pdiffhi=pdiff,
by(age_cat1 age_cat2 age_cat3 inc1 inc2 inc3 imm denom)
format %9.1f pdiff pdifflo pdiffhi
order p1 p0 pdiff pdifflo pdiffhi, last
gsort -age_cat1 -age_cat2 -age_cat3 -inc1 -inc2 -inc3 imm



****************************************************************************
exit
```

```
*************************************************************************
* Hormonal Contraception and Antidepressant Use in Sweden: An
Intersectional Multilevel Analysis of Individual Heterogeneity and
Discriminatory Accuracy (MAIHDA)

*************************************************************************
clear *
global MLwiN_path "C:\Program Files\MLwiN v3.05\mlwin.exe"
set cformat %9.2f



**************************************************************************
* TABLE 1
**************************************************************************

* Load the data
use "final_mlNoMENTAL.dta", clear
keep age_cat1 age_cat2 age_cat3 inc1 inc2 inc3 imm pp proportion denom
order age_cat1 age_cat2 age_cat3 inc1 inc2 inc3 imm pp proportion denom

generate percentage = 100*proportion
drop proportion
format %9.2f percentage

generate age_cat = .
replace age_cat = 1 if age_cat1==1
replace age_cat = 2 if age_cat2==1
replace age_cat = 3 if age_cat3==1

generate inc_cat = .
replace inc_cat = 1 if inc1==1
replace inc_cat = 2 if inc2==1
replace inc_cat = 3 if inc3==1

* Results for the table
tabulate pp [fweight = denom]
table pp [fweight = denom], contents(mean percentage )
tabulate age_cat pp [fweight = denom], column nofreq
tabulate inc_cat pp [fweight = denom], column nofreq
tabulate imm pp [fweight = denom], column nofreq



**************************************************************************
* TABLE 2: MODEL 1
**************************************************************************

* Load the data
use "final_mlNoMENTAL.dta", clear

* IGLS estimation, for MCMC initial values
runmlwin prop cons, ///
  level2(inter: cons) ///
  level1(inter:) ///
  discrete(distribution(binomial) link(logit) denom(denom) mql1) ///
  nopause

* MCMC
runmlwin prop cons, ///
  level2(inter: cons, residuals(u, savechains("m1u.dta",replace))) ///
```

```
  level1(inter:) ///
  discrete(distribution(binomial) link(logit) denom(denom)) ///
  mcmc(burnin(10000) chain(50000) thin(10) savechains("m1b.dta", replace))
///
  initsprevious ///
  nopause

* Level-2 variance
scalar m1sigma2u = [RP2]var(cons)
scalar list m1sigma2u

* Level-1 variance
scalar m1sigma2e = _pi^2/3
scalar list m1sigma2e

* VPC
display "VPC_u = " %9.4f m1sigma2u/(m1sigma2u + m1sigma2e)

* Compress and save the data
compress
save "m1.dta", replace



*----------------------------------------------------------------------------*
* PREPARE FIXED-PART PAREMETER CHAINS
*----------------------------------------------------------------------------*

use "m1b.dta", clear
drop deviance RP2_var_cons_ OD_bcons_1
rename FP1_* b_*
format %9.2f b_*
compress
save "m1b_prepped.dta", replace
isid iteration
codebook iteration, compact



*----------------------------------------------------------------------------*
* PREPARE RANDOM EFFECTS CHAINS
*----------------------------------------------------------------------------*
use "m1u.dta", clear
drop residual idnum
rename value u
format %9.2f u
sort inter iteration
order inter iteration
compress
save "m1u_prepped.dta", replace
isid inter iteration
codebook iteration, compact



*----------------------------------------------------------------------------*
* MERGE DATA, FIXED-PART PARAMETER AND RANDOM EFFECT CHAINS TOGETHER
*----------------------------------------------------------------------------*
use "final_mlNoMENTAL", clear
count
cross using "m1b_prepped.dta"
```

```
count
merge m:1 inter iteration using "m1u_prepped.dta", nogenerate assert(match)
count
compress
save "m1data_prepped.dta", replace


*-------------------------------------------------------------------------*
* ROC
*-------------------------------------------------------------------------*
use "m1data_prepped.dta", clear
count
generate p = invlogit(b_cons + u)
gcollapse (mean) p, by(inter num denom)
count
expand denom
sort inter
bysort inter: generate y = (_n<=numerator)
generate prop = denom/_N
generate weight = int(1/prop)
roctab y p [fw=weight]


*-------------------------------------------------------------------------*
* TABLE 3
*-------------------------------------------------------------------------*
use "m1data_prepped.dta", clear
keep iteration inter age_cat1 age_cat2 age_cat3 inc1 inc2 inc3 imm pp denom
b_cons u
count
generate p = 100*invlogit(b_cons + u)
drop b_cons u
format %9.1f p
drop inter
reshape wide denom p, i(iteration age_cat1 age_cat2 age_cat3 inc1 inc2 inc3
imm) j(pp)
generate denom = denom0 + denom1
drop denom0 denom1
generate pdiff = p1 - p0
gcollapse (mean) p0 p1 pdiff (p2.5) pdifflo=pdiff (p97.5) pdiffhi=pdiff,
by(age_cat1 age_cat2 age_cat3 inc1 inc2 inc3 imm denom)
format %9.1f pdiff pdifflo pdiffhi
order p1 p0 pdiff pdifflo pdiffhi, last
gsort -age_cat1 -age_cat2 -age_cat3 -inc1 -inc2 -inc3 imm


***************************************************************************
* TABLE 2: MODEL 2:
***************************************************************************

* Load the data
use "final_mlNoMENTAL.dta", clear

* IGLS estimation, for MCMC initial values
runmlwin prop cons age_cat2 age_cat3 inc1 inc2 imm pp, ///
  level2(inter: cons) ///
  level1(inter:) ///
  discrete(distribution(binomial) link(logit) denom(denom) mql1) ///
```

```
  nopause

* MCMC
runmlwin prop cons age_cat2 age_cat3 inc1 inc2 imm  pp, ///
  level2(inter: cons, residuals(u,savechains("m2u.dta",replace))) ///
  level1(inter:) ///
  discrete(distribution(binomial) link(logit) denom(denom)) ///
  mcmc(burnin(10000) chain(50000) thin(10) savechains("m2b.dta", replace))
///
  initsprevious ///
  nopause

* Odds ratios
runmlwin, or

* Level-2 variance
scalar m2sigma2u = [RP2]var(cons)
scalar list m2sigma2u

* Level-1 variance
scalar m2sigma2e = _pi^2/3
scalar list m2sigma2e

* VPC
display "VPC_u = " %9.4f m2sigma2u/(m2sigma2u + m2sigma2e)

* Compress and save the data
compress
save "m2.dta", replace

* PCV
display "PCV = " %9.4f (m2sigma2u - m1sigma2u)/m1sigma2u



*------------------------------------------------------------------------------*
* PREPARE FIXED-PART PAREMETER CHAINS
*------------------------------------------------------------------------------*
use "m2b.dta", clear
drop deviance RP2_var_cons_ OD_bcons_1
rename FP1_* b_*
format %9.2f b_*
compress
save "m2b_prepped.dta", replace
isid iteration
codebook iteration, compact



*------------------------------------------------------------------------------*
* PREPARE inter RANDOM EFFECTS CHAINS
*------------------------------------------------------------------------------*
use "m2u.dta", clear
drop residual idnum
rename value u
format %9.2f u
sort inter iteration
order inter iteration
compress
save "m2u_prepped.dta", replace
isid inter iteration
```

```
codebook iteration, compact


*-------------------------------------------------------------------------*
* MERGE DATA, FIXED-PART PARAMETER AND RANDOM EFFECT CHAINS TOGETHER
*-------------------------------------------------------------------------*
use "final_mlNoMENTAL", clear
count
cross using "m2b_prepped.dta"
count
merge m:1 inter iteration using "m2u_prepped.dta"
count
save "m2data_prepped.dta", replace


*-------------------------------------------------------------------------*
* ROC
*-------------------------------------------------------------------------*
use "m2data_prepped.dta", clear
count
generate p = invlogit(b_cons + b_age_cat2*age_cat2 + b_age_cat3*age_cat3 +
b_inc1*inc1 + b_inc2*inc2 + b_imm*imm + b_pp*pp)
gcollapse (mean) p, by(inter num denom)
count
expand denom
sort inter
bysort inter: generate y = (_n<=numerator)
generate prop = denom/_N
generate weight = int(1/prop)
roctab y p [fw=weight]


*-------------------------------------------------------------------------*
* TABLE 3
*-------------------------------------------------------------------------*
use "m1data_prepped.dta", clear
keep iteration inter age_cat1 age_cat2 age_cat3 inc1 inc2 inc3 imm pp denom
b_cons u
count
generate p = 100*invlogit(b_cons + u)
drop b_cons u
format %9.1f p
drop inter
reshape wide denom p, i(iteration age_cat1 age_cat2 age_cat3 inc1 inc2 inc3
imm) j(pp)
generate denom = denom0 + denom1
drop denom0 denom1
generate pdiff = p1 - p0
gcollapse (mean) p0 p1 pdiff (p2.5) pdifflo=pdiff (p97.5) pdiffhi=pdiff,
by(age_cat1 age_cat2 age_cat3 inc1 inc2 inc3 imm denom)
format %9.1f pdiff pdifflo pdiffhi
order p1 p0 pdiff pdifflo pdiffhi, last
gsort -age_cat1 -age_cat2 -age_cat3 -inc1 -inc2 -inc3 imm


***************************************************************************
exit
```

For peer review only

```
pp,imm,inter,age_cat1,age_cat2,age_cat3,inc1,inc2,inc3,proportion,numerator,denom,cons
0,0,12-17 Low income 0 0,1,0,0,1,0,0,.22574355,463,2051,1
1,0,12-17 Low income 0 1,1,0,0,1,0,0,.31181487,512,1642,1
0,1,12-17 Low income 1 0,1,0,0,1,0,0,.12383901,40,323,1
1,1,12-17 Low income 1 1,1,0,0,1,0,0,.23703703,32,135,1
0,0,12-17 Middle income 0 0,1,0,0,0,1,0,.23362993,1024,4383,1
1,0,12-17 Middle income 0 1,1,0,0,0,1,0,.32785809,998,3044,1
0,1,12-17 Middle income 1 0,1,0,0,0,1,0,.13422818,60,447,1
1,1,12-17 Middle income 1 1,1,0,0,0,1,0,.20512821,32,156,1
0,0,12-17 High income 0 0,1,0,0,0,0,1,.28093326,1469,5229,1
1,0,12-17 High income 0 1,1,0,0,0,0,1,.34466448,1166,3383,1
0,1,12-17 High income 1 0,1,0,0,0,0,1,.18867925,50,265,1
1,1,12-17 High income 1 1,1,0,0,0,0,1,.30496454,43,141,1
0,0,18-23 Low income 0 0,0,1,0,1,0,0,.37809917,2013,5324,1
1,0,18-23 Low income 0 1,0,1,0,1,0,0,.40359595,2761,6841,1
0,1,18-23 Low income 1 0,0,1,0,1,0,0,.19350649,149,770,1
1,1,18-23 Low income 1 1,0,1,0,1,0,0,.30901289,144,466,1
0,0,18-23 Middle income 0 0,0,1,0,0,1,0,.36302635,2164,5961,1
1,0,18-23 Middle income 0 1,0,1,0,0,1,0,.38645083,3223,8340,1
0,1,18-23 Middle income 1 0,0,1,0,0,1,0,.19285715,108,560,1
1,1,18-23 Middle income 1 1,0,1,0,0,1,0,.26923078,98,364,1
0,0,18-23 High income 0 0,0,1,0,0,0,1,.39782199,2959,7438,1
1,0,18-23 High income 0 1,0,1,0,0,0,1,.39391655,4701,11934,1
0,1,18-23 High income 1 0,0,1,0,0,0,1,.25,82,328,1
1,1,18-23 High income 1 1,0,1,0,0,0,1,.2887139,110,381,1
0,0,24-30 Low income 0 0,0,0,1,1,0,0,.49862742,9082,18214,1
1,0,24-30 Low income 0 1,0,0,1,1,0,0,.51707798,7857,15195,1
0,1,24-30 Low income 1 0,0,0,1,1,0,0,.32457545,1013,3121,1
1,1,24-30 Low income 1 1,0,0,1,1,0,0,.40912095,619,1513,1
0,0,24-30 Middle income 0 0,0,0,1,0,1,0,.50859779,2869,5641,1
1,0,24-30 Middle income 0 1,0,0,1,0,1,0,.51465154,2090,4061,1
0,1,24-30 Middle income 1 0,0,0,1,0,1,0,.37214136,358,962,1
1,1,24-30 Middle income 1 1,0,0,1,0,1,0,.38345864,153,399,1
0,0,24-30 High income 0 0,0,0,1,0,0,1,.48993289,1971,4023,1
1,0,24-30 High income 0 1,0,0,1,0,0,1,.49569198,1841,3714,1
0,1,24-30 High income 1 0,0,0,1,0,0,1,.37669376,139,369,1
1,1,24-30 High income 1 1,0,0,1,0,0,1,.4585366,94,205,1
```

For peer review only

```
pp,imm,inter,age_cat1,age_cat2,age_cat3,inc1,inc2,inc3,proportion,numerator,denom,cons
0,0,12-17 Low income 0 0,1,0,0,1,0,0,.013224002,279,21098,1
1,0,12-17 Low income 0 1,1,0,0,1,0,0,.038649708,316,8176,1
0,1,12-17 Low income 1 0,1,0,0,1,0,0,.0040497542,28,6914,1
1,1,12-17 Low income 1 1,1,0,0,1,0,0,.0092807421,8,862,1
0,0,12-17 Middle income 0 0,1,0,0,0,1,0,.010099272,587,58123,1
1,0,12-17 Middle income 0 1,1,0,0,0,1,0,.031604379,641,20282,1
0,1,12-17 Middle income 1 0,1,0,0,0,1,0,.0056107035,52,9268,1
1,1,12-17 Middle income 1 1,1,0,0,0,1,0,.019550342,20,1023,1
0,0,12-17 High income 0 0,1,0,0,0,0,1,.008893352,859,96589,1
1,0,12-17 High income 0 1,1,0,0,0,0,1,.021960761,741,33742,1
0,1,12-17 High income 1 0,1,0,0,0,0,1,.0076045627,30,3945,1
1,1,12-17 High income 1 1,1,0,0,0,0,1,.029999999,24,800,1
0,0,18-23 Low income 0 0,0,1,0,1,0,0,.029676914,530,17859,1
1,0,18-23 Low income 0 1,0,1,0,1,0,0,.036956303,1102,29819,1
0,1,18-23 Low income 1 0,0,1,0,1,0,0,.011607248,98,8443,1
1,1,18-23 Low income 1 1,0,1,0,1,0,0,.024938423,81,3248,1
0,0,18-23 Middle income 0 0,0,1,0,0,1,0,.027664155,771,27870,1
1,0,18-23 Middle income 0 1,0,1,0,0,1,0,.029785307,1447,48581,1
0,1,18-23 Middle income 1 0,0,1,0,0,1,0,.011609907,75,6460,1
1,1,18-23 Middle income 1 1,0,1,0,0,1,0,.022484334,61,2713,1
0,0,18-23 High income 0 0,0,1,0,0,0,1,.023347162,1058,45316,1
1,0,18-23 High income 0 1,0,1,0,0,0,1,.024447564,2455,100419,1
0,1,18-23 High income 1 0,0,1,0,0,0,1,.017995911,44,2445,1
1,1,18-23 High income 1 1,0,1,0,0,0,1,.023872679,54,2262,1
0,0,24-30 Low income 0 0,0,0,1,1,0,0,.032189574,2168,67351,1
1,0,24-30 Low income 0 1,0,0,1,1,0,0,.034294236,2553,74444,1
0,1,24-30 Low income 1 0,0,0,1,1,0,0,.013751426,446,32433,1
1,1,24-30 Low income 1 1,0,0,1,1,0,0,.031545039,284,9003,1
0,0,24-30 Middle income 0 0,0,0,1,0,1,0,.030455342,818,26859,1
1,0,24-30 Middle income 0 1,0,0,1,0,1,0,.038922433,838,21530,1
0,1,24-30 Middle income 1 0,0,0,1,0,1,0,.023714487,202,8518,1
1,1,24-30 Middle income 1 1,0,0,1,0,1,0,.029383583,92,3131,1
0,0,24-30 High income 0 0,0,0,1,0,0,1,.025993951,593,22813,1
1,0,24-30 High income 0 1,0,0,1,0,0,1,.027252503,686,25172,1
0,1,24-30 High income 1 0,0,0,1,0,0,1,.023088569,61,2642,1
1,1,24-30 High income 1 1,0,0,1,0,0,1,.021011673,27,1285,1
```

For peer review only

SUPPLEMENTARY

Age	Hormonal contraceptives (%)	Mental health issues (%)
12-17	26.0	7.5
18-23	62.6	14.2
24-30	45.3	16.3
Income		
Low	45.1	16.6
Middle	42.3	12.8
High	48.9	10.0
Immigrant background		
No	48.7	13.5
Yes	24.2	9.4

Supplementary table 1. Summary statistics. Percentage of women within each intersectional dimension using hormonal contraceptives and with previous mental health issues.

Reporting checklist for cohort study.

Based on the STROBE cohort guidelines.

Instructions to authors

Complete this checklist by entering the page numbers from your manuscript where readers will find each of the items listed below.

Your article may not currently address all the items on the checklist. Please modify your text to include the missing information. If you are certain that an item does not apply, please write "n/a" and provide a short explanation.

Upload your completed checklist as an extra file when you submit to a journal.

In your methods section, say that you used the STROBE cohortreporting guidelines, and cite them as:

von Elm E, Altman DG, Egger M, Pocock SJ, Gotzsche PC, Vandenbroucke JP. The Strengthening the Reporting of Observational Studies in Epidemiology (STROBE) Statement: guidelines for reporting observational studies.

For peer review only

		Reporting Item	Page Number
**Title and abstract**			
Title	#1a	Indicate the study's design with a commonly used term in the title or the abstract	1
Abstract	#1b	Provide in the abstract an informative and balanced summary of what was done and what was found	2
**Introduction**			
Background / rationale	#2	Explain the scientific background and rationale for the investigation being reported	4-5
Objectives	#3	State specific objectives, including any prespecified hypotheses	5
**Methods**			
Study design	#4	Present key elements of study design early in the paper	5

Setting	#5	Describe the setting, locations, and relevant dates, including periods of recruitment, exposure, follow-up, and data collection	5-6
Eligibility criteria	#6a	Give the eligibility criteria, and the sources and methods of selection of participants. Describe methods of follow-up.	5-6
Eligibility criteria	#6b	For matched studies, give matching criteria and number of exposed and unexposed	5-6
Variables	#7	Clearly define all outcomes, exposures, predictors, potential confounders, and effect modifiers. Give diagnostic criteria, if applicable	6-7
Data sources / measurement	#8	For each variable of interest give sources of data and details of methods of assessment (measurement). Describe comparability of assessment methods if there is more than one group. Give information separately for for exposed and unexposed groups if applicable.	6-7
Bias	#9	Describe any efforts to address potential sources of bias	6-7
Study size	#10	Explain how the study size was arrived at	5-6
Quantitative variables	#11	Explain how quantitative variables were handled in the analyses. If applicable, describe which groupings were chosen, and why	7-8
Statistical methods	#12a	Describe all statistical methods, including those used to control for confounding	
6-7			
Statistical methods	#12b	Describe any methods used to examine subgroups and interactions	7-8
Statistical methods	#12c	Explain how missing data were addressed	7-8
Statistical methods	#12d	If applicable, explain how loss to follow-up was addressed	n/a Follow-up was complete for the final cohort
Statistical methods	#12e	Describe any sensitivity analyses	

For peer review only

6-7			
**Results**			
Participants	#13a	Report numbers of individuals at each stage of study—eg numbers potentially eligible, examined for eligibility, confirmed eligible, included in the study, completing follow-up, and analysed. Give information separately for for exposed and unexposed groups if applicable.	5-6
Participants	#13b	Give reasons for non-participation at each stage	5-6
Participants	#13c	Consider use of a flow diagram	
Figure 1			
Descriptive data	#14a	Give characteristics of study participants (eg demographic, clinical, social) and information on exposures and potential confounders. Give information separately for exposed and unexposed groups if applicable.	9, table 1
Descriptive data	#14b	Indicate number of participants with missing data for each variable of interest	
n/a All participants included in final analysis had complete data			
Descriptive data	#14c	Summarise follow-up time (eg, average and total amount)	
5-6			
Outcome data	#15	Report numbers of outcome events or summary measures over time. Give information separately for exposed and unexposed groups if applicable.	
9-10			
Main results	#16a	Give unadjusted estimates and, if applicable, confounder-adjusted estimates and their precision (eg, 95% confidence interval). Make clear which confounders were adjusted for and why they were included	10-11

For peer review only

Main results	#16b	Report category boundaries when continuous variables were categorized	11-12
Main results	#16c	If relevant, consider translating estimates of relative risk into absolute risk for a meaningful time period	
10-12			
Other analyses	#17	Report other analyses done—eg analyses of subgroups and interactions, and sensitivity analyses	11-13
**Discussion**			
Key results	#18	Summarise key results with reference to study objectives	13-14
Limitations	#19	Discuss limitations of the study, taking into account sources of potential bias or imprecision. Discuss both direction and magnitude of any potential bias.	15-16
Interpretation	#20	Give a cautious overall interpretation considering objectives, limitations, multiplicity of analyses, results from similar studies, and other relevant evidence.	16
Generalisability	#21	Discuss the generalisability (external validity) of the study results	16
**Other Information**			
Funding	#22	Give the source of funding and the role of the funders for the present study and, if applicable, for the original study on which the present article is based	17

Notes:

- 12d: n/a Follow-up was complete for the final cohort
- 14b: n/a All participants included in final analysis had complete data The STROBE checklist is distributed under the terms of the Creative Commons Attribution License CC-BY. This checklist was completed on 26. January 2021 using https://www.goodreports.org/, a tool made by the EQUATOR Network in collaboration with Penelope.ai

For peer review only

BMJ Open

Population Heterogeneity in Associations Between Hormonal Contraception and Antidepressant Use in Sweden: A Prospective Cohort Study Applying Intersectional Multilevel Analysis of Individual Heterogeneity and Discriminatory Accuracy (MAIHDA)

Journal:	*BMJ Open*
Manuscript ID	bmjopen-2021-049553.R1
Article Type:	Original research
Date Submitted by the Author:	15-Jun-2021
Complete List of Authors:	Zettermark, Sofia; Lund University, Unit for Social Epidemiology, Department of Clinical Sciences, Malmö<br>Khalaf, Kani; Lund University, Unit for Social Epidemiology, Department of Clinical Sciences, Malmö<br>Perez-Vicente, Raquel; Lund University, Unit for Social Epidemiology, Department of Clinical Sciences, Malmö<br>Leckie, George; University of Bristol, Center for Multilevel Modelling, School of Education; Lund University, Unit for Social Epidemiology, Department of Clinical Sciences<br>Mulinari, Diana; Lund University, Department of Gender Studies, Faculty of Social Sciences<br>Merlo, Juan; Lund University, Unit for Social Epidemiology, Department of Clinical Sciences; Region Skane Health Care, Center for Primary Health Care Research, Region Skåne
**Primary Subject Heading**:	Epidemiology
Secondary Subject Heading:	Public health, Mental health, Reproductive medicine
Keywords:	MENTAL HEALTH, Sex steroids & HRT < DIABETES & ENDOCRINOLOGY, SOCIAL MEDICINE, EPIDEMIOLOGY, Depression & mood disorders < PSYCHIATRY

SCHOLARONE™
Manuscripts

I, the Submitting Author has the right to grant and does grant on behalf of all authors of the Work (as defined in the below author licence), an exclusive licence and/or a non-exclusive licence for contributions from authors who are: i) UK Crown employees; ii) where BMJ has agreed a CC-BY licence shall apply, and/or iii) in accordance with the terms applicable for US Federal Government officers or employees acting as part of their official duties; on a worldwide, perpetual, irrevocable, royalty-free basis to BMJ Publishing Group Ltd ("BMJ") its licensees and where the relevant Journal is co-owned by BMJ to the co-owners of the Journal, to publish the Work in this journal and any other BMJ products and to exploit all rights, as set out in our licence.

The Submitting Author accepts and understands that any supply made under these terms is made by BMJ to the Submitting Author unless you are acting as an employee on behalf of your employer or a postgraduate student of an affiliated institution which is paying any applicable article publishing charge ("APC") for Open Access articles. Where the Submitting Author wishes to make the Work available on an Open Access basis (and intends to pay the relevant APC), the terms of reuse of such Open Access shall be governed by a Creative Commons licence – details of these licences and which Creative Commons licence will apply to this Work are set out in our licence referred to above.

Other than as permitted in any relevant BMJ Author's Self Archiving Policies, I confirm this Work has not been accepted for publication elsewhere, is not being considered for publication elsewhere and does not duplicate material already published. I confirm all authors consent to publication of this Work and authorise the granting of this licence.

For peer review only

Population Heterogeneity in Associations Between Hormonal Contraception and Antidepressant Use in Sweden: A Prospective Cohort Study Applying Intersectional Multilevel Analysis of Individual Heterogeneity and Discriminatory Accuracy (MAIHDA)

Sofia Zettermark[1*], Kani Khalaf[1], Raquel Perez-Vicente[1], George Leckie[1,3], Diana Mulinari[2], Juan Merlo[1,4]

[1] Unit for Social Epidemiology, Department of Clinical Sciences, Faculty of Medicine, Lund University, Malmö, Sweden
[2] Department of Gender Studies, Faculty of Social Sciences, Lund University, Lund, Sweden
[3] Center for Multilevel Modelling, School of Education, University of Bristol, Bristol, United Kingdom
[4] Center for Primary Health Care Research, Region Skåne, Malmö, Sweden

*Corresponding author:
Sofia Zettermark
sofia.zettermark@med.lu.se
Unit for Social Epidemiology, Department of Clinical Sciences
Faculty of Medicine, Lund University
Jan Waldenströms gata 35, CRC
214 28, Malmö, Sweden

Word count: 4390

Keywords: Epidemiology, Contraception, Hormonal contraception, Mental health, Multilevel analysis

For peer review only
For peer review only - http://bmjopen.bmj.com/site/about/guidelines.xhtml

ABSTRACT

Objectives From a reproductive justice framework, we aimed to investigate how a possible association between hormonal contraceptive (HC) and anti-depressants use (as a proxy for depression) is distributed across intersectional strata in the population. We aimed to visualize how intersecting power dynamics may operate in combination with HC use to increase or decrease subsequent use of anti-depressants. Our main hypothesis was that the previously observed association between HC and anti-depressants use would vary between strata, being more pronounced in more oppressed intersectional contexts. For this purpose, we applied an intersectional Multilevel Analysis of Individual Heterogeneity and Discriminatory Accuracy (MAIHDA) approach.

Design Observational prospective cohort study using record linkage of national Swedish registers.

Setting The population of Sweden.

Participants All 915 954 women aged 12-30 residing in Sweden 2010, without a recent pregnancy and alive during the individual one-year follow-up.

Primary outcome measure Use of any anti-depressant, meaning being dispensed at least one anti-depressant (ATC N06A) during follow-up.

Results Previously mentally healthy hormonal contraceptive users had an odds ratio of 1.79 for use of anti-depressants compared to non-users, whereas this number was 1.28 for women with previous mental health issues. The highest anti-depressant use were uniformly found in strata with previous mental health issues, with highest usage in women aged 24-30 with no immigrant background, low income, and HC use (51.4%). The largest difference in anti-depressant use between HC users and non-users was found in teenagers, and in adult women of immigrant background with low income. Of the total individual variance in the latent propensity of using antidepressant 9.01% (healthy) and 8.16% (with previous mental health issues) was found at the intersectional stratum level.

Conclusions Our study suggests teenagers and women with immigrant background and low income could be more sensitive to mood effects of HC, a heterogeneity important to consider moving forward.

For peer review only

STRENGTHS AND LIMITATIONS OF THIS STUDY

- Entire Swedish population of women aged 12-30 included
- Pharmacy dispensing automatically linked to individual personal identification number in Sweden through the Swedish Prescribed Drug Register and thus very reliable
- Intersectional MAHIDA is a fruitful way of epidemiologically investigating heterogeneity within a population while considering individual conditions determined by societal power dimensions such as class, gender and race
- Anti-depressant dispensing is not a perfect proxy for depression
- Registers cannot not measure actual use of any medication

For peer review only - http://bmjopen.bmj.com/site/about/guidelines.xhtml

For peer review only

INTRODUCTION

In recent years, attention in the medical community has increasingly been drawn towards depression and other adverse effects on mood related to use of hormonal contraception (HC).(1, 2) Discontinuation rates are high, with mood disturbances or depression being one of the most common complaints.(3-5) Two large epidemiological studies, one in Denmark and the other performed in Sweden, have recently shown a higher risk of anti-depressants and psychotropic drugs use in adolescent users of HC.(6, 7) Randomized controlled trials are rare, but suggest a negative influence of HC on well-being and sexual function,(8, 9) as well as evidence of HC modulating brain activity with subsequent mood alterations in some women.(10, 11) Even though oestrogen and progesterone are known to affect mood,(12) the growing body of evidence in this field is contradictory, with recent reviews concluding that both protective and negative effects of HC on mood exist and more research is needed.(13-16) Despite this uncertainty, many scholars agree that certain subgroups of women seem more vulnerable to psychological side effects of HC than others, particularly teenagers and women with previous mental health issues.(10, 13, 17-20) A call for further investigation into these vulnerable subgroups has been made.(14)

A fruitful way of epidemiologically investigating heterogeneity within a population while considering individual conditions determined by societal power dimensions such as class, gender and race has been developed through intersectional theory in recent years.(21-26) Intersectionality theory was first articulated by Black feminist scholars as a way of understanding how an individual inhabits and is formed by more than one social relation such as gender, “race” or class, and how these classification systems interconnect to create specific contexts of oppression or privilege.(27, 28) These categorizations should not be seen as individual “risky” identities, but as the social, political and economic contextual conditions that outline our lives through structural inequalities.(29) Reproductive justice is a theoretical framework that builds upon intersectionality and centres diverse groups of unprivileged women’s reproductive experiences to recognize that societal context and differing resources available shape reproductive health.(30) Applying a reproductive justice framework, it becomes clear that we need to take notice of disparate sociocultural contexts and interlocking power dimensions to understand different patterns of usage as well as possible diverse responses to HC.(31, 32)

To operationalize an intersectional mapping of heterogeneity in use of anti-depressants in relation to HC on a population level, we used a multilevel analysis of individual heterogeneity and discriminatory accuracy (MAIHDA).(21-23, 33, 34) We created

For peer review only - http://bmjopen.bmj.com/site/about/guidelines.xhtml

intersectional strata based on previous literature showing that age, socioeconomic position, and previous mental illness are relevant intersecting dimensions in understanding the relation between HC and depression.(17, 20, 35, 36)

We conceptualise the intersectional strata as social contexts rather than static individual traits, thereby visualising how intersecting power dynamics can act in combination with HC to predispose for depressive mood. Our main hypothesis was that the previously observed association between HC and use of anti-depressants would vary between strata and that this association would be more pronounced in more oppressed intersectional contexts. We investigate this hypothesis on the whole population of women susceptible to HC use in Sweden.

METHOD

Databases and study population

After allowance from the Swedish Ethical Authority and the data safety committees from Statistics Sweden and the Swedish National Board of Health and Welfare, we obtained a database created by record linkage of several nationwide registers administered by Statistics Sweden (the Swedish Population Register and the Longitudinal Integration Database for Health Insurance and Labour Market Studies, LISA) and the Swedish National Board of Health and Welfare (National Patient Register, the Swedish Prescribed Drug Register (SPDR) and the Cause of Death Register). The Swedish authorities linked the registries using a unique personal identification number, but the database was anonymized before delivering it to us. We defined an initial cohort containing all 1,064,171 women aged 12 - 30 years residing in Sweden 1st January 2010 and obtained individual level data on medication use from SPDR, which contain all dispensed drug prescriptions at Swedish pharmacies since 2006.

Every woman was assigned an individual baseline date, defined by the first dispensed prescription of an HC drug between 1 January 2010 and 31 December 2014 after 12 years of age, and was then followed for one year after her individual baseline date. A woman obtaining her first prescription 1 of September 2013 was therefore followed to the 1 of September 2014. For non-users of HC the baseline date could not be based on a HC-prescription and was therefore assigned, to 1st of July 2012 for all adults, but later for some of the younger girls turning 12 during our period of investigation. This means all non-users had been true non-users for at least 1.5 years before their follow-up started (1 January 2010 to 1 July 2012) but also continued to be non-users all the way to 31 December 2014. From the individual baseline date, the women were followed for one year to find out if a prescription of

For peer review only - http://bmjopen.bmj.com/site/about/guidelines.xhtml

an antidepressant was dispensed. Data was also collected on psychiatric disorders and psychotropic drug use in the past three years (see Assessment of variables). After excluding women with incomplete follow-up time due to death, emigration, missing information on country of birth, and pregnancies one year before and after the baseline as well as, the final database consisted of 915 952 women. This database was divided into two cohorts according to the presence or absence of previous mental health issues, see Figure 1.

Assessment of variables

Users of HC were defined as any women who, according the SPDR, filled a prescription of HC (Anatomical Therapeutical Chemical (ATC) classification system codes G02B, G03AA-C) between 1 January 2010 and 31 December 2014, while non-users did not have a prescription filled during the same period. Emergency contraception (G03AD) that are mainly bought over the counter in Sweden was excluded. The majority of HC prescriptions are acquired via midwifes in Sweden (86.0% in our original cohort), whom can only prescribe HC for contraceptive purposes. Physicians, most often gynecologists, can also can prescribe HC for other purposes such as in response to bleeding disturbances or endometriosis. Since these indications could confound our results, we excluded women with physician-issued prescriptions, see Figure 1. HC prescriptions can be dispensed by pharmacies annually or every three months.

Anti-depressant use, the outcome of our study, was defined, according to the SPDR, as being dispensed at least one prescription of antidepressants (ATC: N06A) during the individual one-year follow-up.

Previous mental health issues were defined as having any psychiatric disorder diagnosed at a hospital (ICD: F00-F99) or a dispensed prescription of a psychotropic drug (ATC: N05A, N05B, N06A) in the past three years.

Pregnancies one year previous to baseline and during follow-up were identified according to the 2019 version of the Nordic Diagnosis-Related Group classification (NordDRG), Major Diagnostic Categories codes M14 for pregnancy, delivery and post-partum care.(37)

We used family level data on income as of 31 December 2010 from Statistics Sweden's LISA. Individualized disposable family income was calculated by dividing the total disposable income of the family by the number of family members, taking into account the different consumption weights of adults and children determined by Statistics Sweden. Thereafter, we created three categories (i.e., low, medium, and high) of income using tertile

For peer review only - http://bmjopen.bmj.com/site/about/guidelines.xhtml

cut-offs based on the total Swedish population aged 18 - 80 years. We considered the high-income category as the reference in the comparisons.

We defined immigrant status at the family level as no family member >18 years of age born in Sweden, since understanding of and access to institutions such as health care differ depending on social position such as it is constructed by the power dimensions of race/immigration, as well as the experience of xenophobia. This variable should therefore be considered as an effort to capture a social position affecting possibilities and life trajectories rather than an essentialist view of otherness.We categorized age at the individual baseline into the following groups: 12 to 17, 18 to 23, and 24 to 30 years to capture age specific conditions of adolescents, young adults, and adult women.

Intersectional Strata

Within each cohort stratified by previous mental health issues, we generated 36 intersectional strata by combining three categories of age, three categories of income, two categories of immigrant background, and two categories of HC use. Mental health issues can be considered as a valid category of intersectional investigation in a society that considers an able body and mind vital, in other words relating to the power dimension of able-bodiedness,(38, 39). Mental health issues were also included in the analysis since they are a strong determinant of antidepressant use that needs to be addressed. We could consider that over and above individual characteristics, mental illness-related stigma may condition inequities in health care.(40) As with gender or income, able-bodiedness concerning mental health can therefore be conceptualized as a contextual dimension when defining intersectional strata.

Statistical analysis

We performed an intersectional MAIHDA with individual women at the first level of analysis and the 36 intersectional strata at the second level, stratified by previous mental health issues (See Supplementary material 1-4). The use of antidepressants in the population was thus analysed through two successive multilevel logistic regression models distinguishing between measures of association and measures of variance and discriminatory accuracy.

Model 1

The first model included only an intercept and a random effect for the intersectional strata with no covariates. In this model 1 we first (i) performed a simple analysis of components of variance and calculated the Variance Partition Coefficient (VPC). That is, the share

For peer review only - http://bmjopen.bmj.com/site/about/guidelines.xhtml

(expressed as a percentage) of the total individual variance in the latent propensity of antidepressant use that is at the intersectional strata level. In this simple model, the VPC correspond with the Intraclass Correlation Coefficient (ICC) which informs on the clustering of antidepressant use within intersectional strata. The VPC values extend from 0 to 100%. Second, (ii) we calculate the stratum-specific absolute usage of anti-depressants and their 95% credible intervals (CI) by transformation of the information from the logistic regression to the probability scale. We used this information to map the user heterogeneity across the intersectional strata. Then, (iii) using these stratum-specific predictions, we calculated the Area Under the receiver operator characteristics Curve (AUC). The AUC informs on the accuracy of the intersectional strata information for discriminating those women who used antidepressants from those who did not. The AUC values extend from 0.5 to 1, where 0.5 represent absence of accuracy and 1 represents total accuracy. Both the VPC and the AUC in model 1 can be interpreted as measures of discriminatory accuracy,(41) and inform on the magnitude of the general intersectional effects. The higher the VPC and AUC values, the higher the influence of the intersectional context on individual use of antidepressants. Finally, (iv) we calculated the difference in anti-depressant use and 95% CI between similar pairs of strata differing only on the use of HC. This represents the stratum specific association between HC and antidepressant use.

Model 2 or fixed main effects model

This model includes the fixed, main effects of all the intersectional dimensions (i.e., age, income, immigrant background, and HC use) used to define the intersectional strata. In model 2 we quantified, (i) the association between the intersectional dimensions and use of antidepressants as expressed by odds ratio (OR) and 95% CI. We also to calculate (ii) the Proportional Change in the Variance (PCV). The PCV measures the overall proportion of strata variance of model 1 explained by the specific intersectional dimensions. Since model 2 contains all the variables used to construct the intersectional strata as main effects, it should explain all the strata variance (i.e., PCV= 100%). If this is not the case, the remaining between strata variance would be due to the existence of multiplicative interaction of effects between the intersectional dimensions defining the strata.(22, 42)

The AUCs of the models 1 and 2 are expected to be the same because model 2 only decomposes the stratum-specific predicted probabilities obtained in model 1 into fixed and random effect components and their sum equals the prediction obtained only by random effects in model 1.

For peer review only - http://bmjopen.bmj.com/site/about/guidelines.xhtml

We ran the models using MLwiN 3.00 by calling it from within Stata 14.1 using the *runmlwin* command.(43) The estimations were performed using Markov chain Monte Carlo (MCMC) methods. All points estimations and their 95% credible intervals were based on the parameter and random effect chains obtained from the MCMC estimation. See elsewhere for further information on the statistical MAIHDA analysis including Stata commands,(33, 42) and discussion on the theory and methodological approach.(22, 44)

Patient and Public Involvement statement

The research was developed with a grassroot perspective in mind, whereby women's experiences of use of hormonal contraception inspired and informed the choice of research area and research questions. The anonymised data and scope of the study, including around 1 million women, prohibited direct patent involvement.

RESULTS

Characteristics of the population

The selection of the study population is shown in Figure 1. Out of the 915 952 women 12.4% (n = 113 711) had previous mental health issues. Mean age was somewhat older for women with previous mental health issues (22.5 years; SD 4.8) than for those without such concerns (20.8 years; SD 5.3). Supplementary material 5 shows pooled statistics for usage of previous mental health issues and HC use, while Supplementary material 6 displays a frequency table over all included HC. Table 1 displays the baseline characteristics of the population by previous mental health issues and use of hormonal contraceptives.

Table 1. Characteristics of the 915 954 women aged 12 - 30 years by previous mental health issues and use of hormonal contraceptives. Values are percentages (number of women in parenthesis).

	Previous mental health issues			
	Yes 12.4 (113 711)		No 87.6 (802 243)	
	Use of HC		Use of HC	
	Yes 42.5 (48 302)	No 57.5 (65 409)	Yes 42.0 (337 297)	No 58.0 (464 946)
Antidepressant during follow-up	41.2 (19 886)	39.8 (26 013)	2.7 (9 215)	1.9 (8 699)
Age				
12-17 years	14.2 (6 838)	19.4 (12 698)	16.7 (56 343)	42.1 (195 937)
18-23 years	48.3 (23 347)	31.2 (20 381)	50.1 (168 968)	23.3 (108 939)

For peer review only - http://bmjopen.bmj.com/site/about/guidelines.xhtml

24-30 years	37.5 (18 117)	49.4 (32 330)	33.2 (11 986)	34.6 (160 616)
Income level				
Low inc.	40.4 (19 513)	45.6 (29 803)	31.8 (107 119)	33.1 (154 098)
Medium inc.	27.1 (13 078)	27.5 (17 954)	25.4 (85 620)	29.5 (137 098)
High inc.	32.5 (15 711)	27.0 (17 652)	42.9 (144 558)	37.4 (173 750)
Immigrant background				
None	94.6 (45 674)	89.1 (58 264)	94.2 (317 716)	82.6 (383 878)
Yes	5.4 (2 628)	10.9 (7 145)	5.8 (19 581)	17.4 (81 068)

The share of HC users was very similar in healthy women and those with previous mental health issues, 42.0% and 42.5%, respectively. Anti-depressants were dispensed to 2.7% of HC users compared to 1.9% of non-users among healthy women during follow-up. For women with previous mental health issues, 41.2% of HC users and 39.8% of non-users dispensed an anti-depressant prescription. The income levels were generally higher among women without mental health issues, and HC users were somewhat more affluent in both cohorts.

Results from the MAIHDA

Table 2 shows the results from the MAIHDA distinguishing between measures of association and measures of variance and discriminatory accuracy.

For peer review only

Table 2. Results from the Multilevel Analysis of Individual Heterogeneity and Discriminatory Accuracy (MAIHDA) distinguishing between measures of association (Odds Ratios) and measures of variance and

For peer review only - http://bmjopen.bmj.com/site/about/guidelines.xhtml

discriminatory accuracy. The analyses are stratified by the existence of previous mental issues. Values are point estimations (with 95% credible intervals) or percentages where indicated.

	Without metal health issues		With mental health issues	
	Model 1	Model 2	Model 1	Model 2
**Measures of association**				
Age				
12-17 years		Reference		Reference
18-23 years		1.78 (1.36-2.42)		1.57 (1.38-1.76)
24-30 years		2.09 (1.65-2.70)		2.66 (2.36-3.00)
Income				
High inc.		Reference		Reference
Medium inc.		1.05 (0.78-1.37)		0.87 (0.77-0.98)
Low inc.		1.10 (0.81-1.41)		0.87 (0.77-0.98)
Immigrant background				
None		Reference		Reference
Yes		0.63 (0.49-0.79)		0.55 (0.49-0.61)
Hormonal contraception				
No		Reference		Reference
Yes		1.62 (1.34-2.06)		1.19 (1.08-1.31)
**Measures of variance and discriminatory accuracy***				
Variance	0.30 (0.18-0.50)	0.10 (0.06-0.18)	0.29 (0.18-0.49)	0.02 (0.01-0.03)
VPC	8.45%	3.02%	8.18%	0.49%
PCV		66.29%		94.48%
AUC	0.62 (0.62-0.62)	0.62 (0.62-0.62)	0.64 (0.64-0.64)	0.64 (0.64-0.64)

*Between-strata variance, variance partition coefficient (VPC), proportional change of the variance (PCV), Area under the curve (AUC)

288

289 Model 1 indicates that 8.45% (without mental health issues) and 8.18% (with previous mental
290 health issues) of the total individual variance in the latent propensity of using antidepressant is
291 at the intersectional strata level. These VPCs correspond with AUC values of 0.62 and 0.64
292 respectively. Both measures suggest the existence of a moderate intersectional effect. The
293 PCV was high in both groups, but especially so in the group with previous mental health
294 issues, meaning the intersectional dimensions or main effects explain more of the inter-strata
295 variance for these women. Model 2 shows that HC was associated with increased usage of
296 antidepressants after adjustment for all other intersectional dimensions. This result was seen
297 within both cohorts, but more strongly so in women without previous mental health issues

For peer review only - http://bmjopen.bmj.com/site/about/guidelines.xhtml
For peer review only

(OR 1.62 compared to 1.19). Finally, the VPC in model 2 was very small (3.02% and 0.49% respectively) but did not vanish. This finding means that while the intersectional strata effect was mainly due the additive effect of variables defining the strata, a small component due to interaction of effects could also be detected.

Heterogeneity concerning antidepressant use in our cohort

Women with previous mental health issues had a much higher usage of antidepressants than women without such issues, but the association with HC use nonetheless varied across the other intersectional dimensions. Table 3 show the stratum-specific incidence rates for antidepressant use and 95% CI obtained in model 1.

Table 3. Distribution of antidepressant use between different intersectional strata, and difference in usage between user and non-users of hormonal contraceptives but otherwise sharing the same intersectional stratum. The values are calculated from the multilevel analysis of individual heterogeneity and discriminatory accuracy (MAIHDA). Numbers are percentages.

Previous mental health issues	Age (years)	Income level	Immigrant background	Number of women	Use of hormonal contraceptives (%)		
					Yes	No	Yes-No difference
No	12 – 17	Low	No	28182	3.7	1.3	**2.4 (1.9 , 2.8)**
			Yes	7643	1.2	0.5	**0.7 (0.1 , 1.5)**
		Middle	No	75836	3.0	1.0	**2.0 (1.8 , 2.3)**
			Yes	10110	1.8	0.6	**1.2 (0.5 , 2.1)**
		High	No	125903	2.0	0.9	**1.1 (0.9 , 1.2)**
			Yes	4606	2.5	0.8	**1.6 (0.6 , 2.8)**
	18 – 23	Low	No	44723	3.5	3.0	**0.5 (0.2 , 0.9)**
			Yes	11174	2.3	1.2	**1.1 (0.5 , 1.7)**
		Middle	No	72018	2.8	2.8	0.1 (-0.2 , 0.3)
			Yes	8776	2.3	1.2	**1.1 (0.5 , 1.8)**
		High	No	136284	2.3	2.3	0 (-0.2 , 0.1)
			Yes	4386	2.0	1.8	**0.2 (-0.6 , 0.9)**
	24 – 30	Low	No	130127	3.1	3.2	-0.1 (-0.3 , 0.1)
			Yes	39368	2.7	1.4	**1.3 (0.9 , 1.7)**
		Middle	No	45013	3.6	3.0	**0.5 (0.2 , 0.9)**
			Yes	10965	2.7	2.4	0.4 (-0.3 , 1.1)
		High	No	43508	2.4	2.6	-0.2 (-0.5 , 0.1)
			Yes	3621	1.9	2.3	-0.3 (-1.3 , 0.7)
Yes	12 – 17	Low	No	3402	30.5	22.7	**7.8 (4.7 , 10.8)**
			Yes	434	20.8	13.7	7.1 (-0.3 , 15.1)
		Middle	No	6854	31.2	23.4	**7.8 (5.6 , 10.1)**

For peer review only - http://bmjopen.bmj.com/site/about/guidelines.xhtml

		Yes	569	19.9	14.2	5.7 (-1.2 , 13.1)
	High	No	7906	34.2	28.1	**6.1 (3.9 , 8.3)**
		Yes	371	30.4	19.8	**10.6 (1.4 , 19.9)**
18 – 23	Low	No	10937	39.2	37.8	1.4 (-0.4 , 3.2)
		Yes	1127	28.5	19.7	**8.8 (3.4 , 14.4)**
	Middle	No	12915	37.8	36.3	1.5 (-0.2 , 3.1)
		Yes	844	27.4	19.7	**7.7 (1.9 , 13.7)**
	High	No	17276	38.3	39.8	-1.5 (-3 , 0)
		Yes	629	28.1	25.4	2.8 (-4 , 9.4)
24 – 30	Low	No	29333	50.1	49.9	0.2 (-1 , 1.4)
		Yes	4083	37.3	32.4	**4.9 (1.5 , 8.4)**
	Middle	No	8629	49.7	50.8	-1.1 (-3.4 , 1.1)
		Yes	1221	33.5	37.1	-3.6 (-10 , 2.6)
	High	No	6686	48.5	48.9	-0.4 (-2.9 , 2)
		Yes	495	43.7	37.5	6.3 (-3.2 , 15.8)

The highest use of anti-depressants were observed in non-immigrant women, aged 24-30, with previous mental health issues, using HC and with low income (50.1%). The lowest usage were found in teenagers without previous mental health issues and no HC use, especially in the strata of immigrant girls from low (0.50%) and middle-income (0.60%) households.

Heterogeneity concerning the association between hormonal contraceptive and antidepressant use

Overall, the propensity to use antidepressants was consistently higher in HC users compared to non-users in younger women between 12 and 17 years of age, both without previous mental health issues (0.7 – 2.4 percentage points), and with a mental health history (5.7 – 7.8 percentage points) with the magnitude being higher in the latter group. However, the 95% credible intervals were broad since the number of individuals was relatively small in these latter strata. Table 3 gives detailed information on these associations. In adolescents the tendency was that an immigrant background lowered the use of anti-depressants, while the opposite was true for adult women, where a positive association between HC use and later antidepressant use was mainly found in women with low income and immigrant background, again with higher magnitudes in women with previous mental health issues. The association between HC and antidepressant use was smaller in adult women native to Sweden no matter their income, and completely disappeared in adult women with high income regardless of immigrant background.

For peer review only - http://bmjopen.bmj.com/site/about/guidelines.xhtml

DISCUSSION

The main hypothesis of our study was that the previously observed association between HC and antidepressant use, mainly seen in adolescent girls(6-9, 17, 45), would be modified by the intersectional context of the women, being more pronounced in more oppressed intersectional contexts. We confirmed that subsequent use of anti-depressants after an HC prescription compared to non-users of HC within the same intersectional context was heterogenous across intersectional strata pairs. As hypothesized, the difference in propensity to use anti-depressants was more pronounced in more oppressed intersectional contexts like those composed by immigrant, low-income women with previous mental issues. That is, the use of antidepressants and to some extent the difference in use between HC users and non-users varied mainly depending on previous mental health issues, but the HC-antidepressant association was considerably modified across pair of strata with other characteristics equal but where HC use and non-use differed, in both cohorts. Aside from adolescent girls, low and middle income adult women with immigrant background had a more pronounced difference in propensity for using anti-depressants, while adult women without immigrant background had both the lowest anti-depressant use and a low grade of modification by HC use.

Independently of previous mental health issues, the propensity for using anti-depressants was consistently higher for HC users than for non-users in teenagers aged 12-17, a result aligned with previous studies that has found a heterogeneous response with regard to both age and other factors.(6, 7, 17, 18, 20, 45-47) As discussed in a previous paper, this higher risk for adolescents could be due to a *selective discontinuation bias,*(7) a development of the *healthy worker survivor effect,* describing how bias is introduced through a continuous selection where those staying in the workforce are healthier than those who leave.(48) Women who experience a negative influence of HC on psychological health might discontinue treatment in early ages, while those without symptoms continued on HC into adulthood, creating this age-dependent *selective discontinuation bias*. This could explain why the observed association between HC and adverse mental health outcomes are stronger in adolescents. Most Swedish women do however continue their HC treatment with the same method.(49) A previous study found that new users of HC has a higher risk of obtaining anti-depressants within the first six months of HC use than continuous users.(6) To address this possible bias we ran a sensitivity analysis differentiating between women who filed a first prescription of an HC for the first time during the study period (26.2% of HC users) and those

For peer review only - http://bmjopen.bmj.com/site/about/guidelines.xhtml

that had a repeat prescription. The results showed that in our cohort the association between HC use and subsequent anti-depressant use was very similar in new and continuous users (Odds Ratio 1.52 and 1.45, respectively, with overlapping 95% confidence intervals).

As expected, among adult women the overall propensity for using anti-depressants was higher, as it is known that anti-depressant use increases by age,(50,51) and the difference between HC users and non-users was smaller. Women native to Sweden had a higher propensity for using anti-depressants, but this was moderated by HC exposure to a lower extent than for immigrant women. In adult women native to Sweden, HC use gave no increase of antidepressant use among those with high income. The lower utilization of anti-depressants does not necessarily mean that immigrant women are healthier, since earlier studies have found immigrants utilize healthcare to a lesser extent, even though the need is pronounced, with reasons including discrimination.(52,53) A recent study found that adjustement for health care access eliminatied the association between HC initiation and subsequent anti-depressant use in a US population.(54) Although the health care system is different in Sweden and visits to midwifes for contraceptive purposes free, we conducted a sensitivity analysis including only women who had accessed health care within the last three years to adress this. Using only care-accessors as the reference group did not change our results in any substansive way, see Supplementary material 7 .

Intersectional considerations

The big difference in anti-depressant consumption depending on HC use for lower income immigrant women could be interpreted as the intersectional contexts embodied by these women are more susceptible to the potential detrimental effect of HC on mood. The interrelating negative consequences of low income as a proxy for class or social position, gender and xenophobia may accumulate over the life course and lead to a higher vulnerability to exposures that predispose for antidepressant use later in life,(55-57) whereas this diverse vulnerability to HC exposure might not be visible in teenagers. Social experiences can vary depending on for example social position, which in turn impact psychological development, mood and cognition, thus influencing health.(58, 59) In understanding how HC can impact women's mental health differently, both possible individual biological predispositions and social settings need to be investigated, since the emotional response to HC is influenced by context.(32) In other words, the interlocking power axes that create oppression could predispose women already under structural burdens for adverse mental health reactions when using HC. The fact that adult women native to Sweden were almost unaffected by HC use,

For peer review only - http://bmjopen.bmj.com/site/about/guidelines.xhtml

could strengthen this suggestion. Without the intersectional strata this disparity would not have been so easily identified and visualized.

Focusing on women whose lives are affected by several interlocking power dimensions such as low social position and xenophobia is fundamental to achieving reproductive justice.(30) Nonetheless, our intersectional strata should not be considered static categories of inherently "risky identities" but must be interpreted as context specific vulnerabilities of women within certain interlocking positions, constituted in relation to power dynamics created by unequal schemes such as the economic system.(25, 29) It is likely that in other contexts, other groups could be more vulnerable. It is also important to remember that the purpose of HC most commonly is protection against unwanted pregnancy, a situation that if it arises in itself can have negative mental health effects. In identifying the underlying power systems creating these intersectional categories and acknowledging their constant movement and changing dynamics on a societal level, it furthermore becomes possible to address these inequalities through social change.

In this study, we have combined a classical epidemiological approach of exposure to HC and an intersectional MAHIDA to create a novel understanding of how intersecting power dynamics could create particular vulnerabilities to this specific exposure. Because of our study design, where women are followed for one year after a dispensed prescription of HC, it is more theoretically coherent to view use of HC as an exposure rather than a component of the intersectional strata. However, it is possible to within our approach view HC use as a socio-contextual factor that captures certain living conditions (for example more likely to be sexually active or in a heterosexual relationship), which somewhat changes the interpretation of the results. This epistemological tension is not necessarily a limitation, but could enrich the dialogue in social epidemiology on whether it is possible to separate contextual factors from "pure" exposure.(60-62)

Limitations

The findings from this study must be interpreted in the context of its limitations. The SPDR has highly reliable data on dispensed prescriptions but cannot measure the actual use of dispensed medications. Along the same line of reasoning, whether the women was exposed to HC treatment during her entire follow-up is not possible to determine with our method, although previous Swedish data suggest continuation rates for any HC after 6 months are almost 90%.(47) Use of anti-depressants can be considered a proxy for depression, but anti-depressants are also prescribed for other reasons than depression, including generalized

For peer review only - http://bmjopen.bmj.com/site/about/guidelines.xhtml

anxiety disorder, obsessive-compulsive disorder and panic disorder.(63) Therefore it is not a perfect proxy of depression but may be a more general indication of impaired mental health.(64) However, out of all women with potentially unfavorable mental health effects from HC, only a subset would have symptoms severe enough to get an anti-depressant prescription, leading instead to many missed cases. Since the outcome is rather common, the risk of underestimation is further enhanced and the true risk of adverse mental health effects could be higher.

As in any observational study, ours only allows for measurements of associations and cannot determine causation. Furthermore, apparently strong average associations do not necessarily convey a high discriminatory accuracy (see elsewhere for a short review and discussion).(65) Nevertheless, since our analysis yielded a moderate accuracy (i.e., AUC=0.6), the intersectional strata do matter for the propensity to use antidepressants. A consideration in every quantitative intersectional study is the basis for creating intersectional categories, since comprehensive information on background and lived experiences are lacking and the categories are created based on available but crude proxies such as income level. For example, in our study the group of women with immigrant background was very heterogenous, so we cannot exclude that the increased antidepressant use is located on more specific country of birth categories. There is an ongoing debate whether these crude categorizations are feasible, and extra caution should be taken when investigating emerging intersectional categories rather than established ones.(66)

Conclusion

It is important to recognise intersectional perspectives and interacting axes of oppression to tailor better public health interventions, as well as acknowledging the experiences of oppressed women to reach reproductive and social justice. (29, 66) Our intersectional MAIHDA methodology operationalizes this idea by providing information on the discriminatory accuracy of the contexts that define the intersectional strata. It highlights the need to consider disadvantages consisting of several interlocking structural dimensions such as income/class, age and immigration to better understand how HC might predispose certain women, mainly teenagers and low-income women with immigrant background, for depression. These vulnerabilities are based in inequalities that are not static, but structurally created and therefore possible to redeem.

For peer review only - http://bmjopen.bmj.com/site/about/guidelines.xhtml

Figure 1. Selection of the study population.

Acknowledgements

A previous version of this study was presented as a poster at the Gynecological Endocrinology, the 19th World Congress in December 2020. We thank all colleagues at the Unit for Social epidemiology, Lund university, for valuable discussions.

Ethical statement

The database was approved by the Regional Ethical Review Board in Lund, Sweden, the Data Safety Board at Statistics Sweden and the National Board of Health and Welfare (Dnr: 2014/856, 2015/341).

Funding statement

This work was supported by The Swedish Research Council (Vetenskapsrådet) grant number: [2017-01321] https://www.swecris.se/betasearch/details/project/201701321VR

Competing interests

None declared.

Data sharing statement

Public access to the data is restricted by the Swedish Authorities (Public Access to Information and Secrecy Act; http://www.government.se/information-material/2009/09/public-access-to-information-and-secrecy-act/) but data can be made available for researchers after a special review that includes approval of the research project by both an Ethics Committee and the authorities' data safety committees. The National Board of Health and Welfare is a government agency under the Ministry of Health and Social Affairs. It is not their policy to provide individual level data to researchers abroad. Instead, they normally advise researchers in other countries to cooperate with Swedish colleagues, to whom they can provide data according to standard legal provisions and procedures. Requests for access to the data can be made to the National Board of Health and Welfare and Statistics

For peer review only - http://bmjopen.bmj.com/site/about/guidelines.xhtml

Sweden (http://www.socialstyrelsen.se/statistics; https://www.scb.se/en/services/guidance-for-researchers-and-universities/).

Contributorship statement

Sofia Zettermark: Conceptualization, design, analysis, interpretation of data, writing original draft, final approvement of version to be published.

Kani Kahlaf: Interpretation of data, revising draft critically for intellectual content, final approvement of version to be published.

Raquel Perez-Vicente: Design, analysis, interpretation of data, revising draft critically for intellectual content, final approvement of version to be published.

George Leckie: Analysis, interpretation of data, revising draft critically for intellectual content, final approvement of version to be published.

Diana Mulinari: Interpretation of data, revising draft critically for intellectual content, final approvement of version to be published.

Juan Merlo: Conceptualization, design, analysis, interpretation of data, revising draft critically for intellectual content, final approvement of version to be published.

REFERENCES

1 Bitzer J. Hormonal contraception and depression: another Pill scandal? *Eur J Contracept Repr* 2017;22(1):1-2.

2 Frokjaer VG. Pharmacological sex hormone manipulation as a risk model for depression. *J Neurosci Res* 2020;98(7):1283-92.

3 Rosenberg MJ, Waugh MS. Oral contraceptive discontinuation: a prospective evaluation of frequency and reasons. *Am J Obstet Gynecol* 1998;179(3;1):577-82.

4 Simmons RG, Sanders JN, Geist C, et al. Predictors of contraceptive switching and discontinuation within the first 6 months of use among Highly Effective Reversible Contraceptive Initiative Salt Lake study participants. *Am J Obstet Gynecol* 2019;220(4):376e1-e12.

5 Sanders SA, Graham CA, Bass JL, et al. A prospective study of the effects of oral contraceptives on sexuality and well-being and their relationship to discontinuation. *Contraception* 2001;64(1):51-8.

6 Skovlund CW, Morch LS, Kessing LV, et al. Association of Hormonal Contraception With Depression. *JAMA Psychiat* 2016;73(11):1154-62.

7 Zettermark S, Perez Vicente R, Merlo J. Hormonal contraception increases the risk of psychotropic drug use in adolescent girls but not in adults: A pharmacoepidemiological study on 800 000 Swedish women. *PLoS One* 2018;13(3):e0194773 doi:10.1371/journal.pone.0194773.

For peer review only - http://bmjopen.bmj.com/site/about/guidelines.xhtml

8 Zethraeus N, Dreber A, Ranehill E, et al. A first-choice combined oral contraceptive influences general well-being in healthy women: a double-blind, randomized, placebo-controlled trial. *Fertil Steril* 2017;107(5):1238-45.

9 Lundin C, Malmborg A, Slezak J, et al. Sexual function and combined oral contraceptives - a randomised, placebo-controlled trial. *Endocr Connect* 2018;7(11):1208-1216 doi:10.1530/EC-18-0384

10 Gingnell M, Engman J, Frick A, et al. Oral contraceptive use changes brain activity and mood in women with previous negative affect on the pill--a double-blinded, placebo-controlled randomized trial of a levonorgestrel-containing combined oral contraceptive. *Psychoneuroendocrino* 2013;38(7):1133-44.

11 Fisher PM, Larsen CB, Beliveau V et al. Pharmacologically Induced Sex Hormone Fluctuation Effects on Resting-State Functional Connectivity in a Risk Model for Depression: A Randomized Trial. *Neuropsychopharmacol* 2017;42(2):446-53.

12 Toffoletto S, Lanzenberger R, Gingnell M, et al. Emotional and cognitive functional imaging of estrogen and progesterone effects in the female human brain: A systematic review. *Psychoneuroendocrino* 2014;50:28-52.

13 Schaffir J, Worly BL, Gur TL. Combined hormonal contraception and its effects on mood: a critical review. *Eur J Contracept Repr* 2016;21(5):347-55.

14 Robakis T, Williams KE, Nutkiewicz L, et al. Hormonal Contraceptives and Mood: Review of the Literature and Implications for Future Research. *Curr Psychiat Rep* 2019;21(7):57. doi:10.1007/s11920-019-1034-z

15 Lewis CA, Kimmig AS, Zsido RG, et al. Effects of Hormonal Contraceptives on Mood: A Focus on Emotion Recognition and Reactivity, Reward Processing, and Stress Response. *Curr Psychiat Rep* 2019;21(11):115.

16 Worly BL, Gur TL, Schaffir J. The relationship between progestin hormonal contraception and depression: a systematic review. *Contraception* 2018;97(6):478-89.

17 de Wit AE, Booij SH, Giltay EJ, et al. Association of Use of Oral Contraceptives With Depressive Symptoms Among Adolescents and Young Women. *JAMA Psychiat* 2020;77(1):52-59 doi:10.1001/jamapsychiatry.2019.2838

18 Gregory ST, Hall K, Quast T, et al. Hormonal Contraception, depression, and Academic Performance among females attending college in the United States. *Psychiat Res* 2018;270:111-6.

19 Lundin C, Danielsson KG, Bixo M, et al. Combined oral contraceptive use is associated with both improvement and worsening of mood in the different phases of the treatment cycle - A double-blind, placebo-controlled randomized trial. *Psychoneuroendocrino* 2017;76:135-43.

20 Bengtsdotter H, Lundin C, Gemzell Danielsson K, Bixo M, Baumgart J, Marions L, et al. Ongoing or previous mental disorders predispose to adverse mood reporting during combined oral contraceptive use. *Eur J Contracept Repr* 2018;23(1):45-51.

21 Wemrell M, Mulinari S, Merlo J. An intersectional approach to multilevel analysis of individual heterogeneity (MAIH) and discriminatory accuracy. *Soc Sci Med* 2017;178:217-9.

22 Merlo J. Multilevel analysis of individual heterogeneity and discriminatory accuracy (MAIHDA) within an intersectional framework. *Soc Sci Med* 2018;203:74-80.

23 Evans CR, Williams DR, Onnela JP, et al. A multilevel approach to modeling health inequalities at the intersection of multiple social identities. *Soc Sci Med* 2018;203:64-73.

24 Bauer GR. Incorporating intersectionality theory into population health research methodology: Challenges and the potential to advance health equity. *Soc Sci Med* 2014;110:10–7.

25 McCall L. The complexity of intersectionlity. *Signs* 2005;30:1771-800.

For peer review only - http://bmjopen.bmj.com/site/about/guidelines.xhtml

26 Evans CR, Erickson N. Intersectionality and depression in adolescence and early adulthood: A MAIHDA analysis of the national longitudinal study of adolescent to adult health, 1995-2008. *Soc Sci Med* 2019;220:1-11.
27 Crenshaw K. Mapping the Margins: Intersectionality, Identity Politics, and Violence against Women of Color. *Stanford Law Rev* 1991;43(6):1241-99.
28 Cho S, Crenshaw K, McCall L. Toward a field of Interscetionality Studies: Theory, Applications and Praxis. *Signs* 2013;38(4):785-810.
29 Price K. It's Not Just About Abortion: Incorporating Intersectionality in Research About Women of Color and Reproduction. *Women Health Iss* 2011;21(3):55-7.
30 Ross LJ, Rickie S. Reproductive Justice: An Introduction. Oakland, CA: University of California Press 2017:1-360.
31 Dehlendorf C, Ruskin R, Grumbach K, et al. Recommendations for intrauterine contraception: a randomized trial of the effects of patients' race/ethnicity and socioeconomic status. *Am J Obstet Gynecol* 2010;203(4):319e1-8.
32 Montoya ER, Bos PA. How Oral Contraceptives Impact Social-Emotional Behavior and Brain Function. *Trends Cogn Sci* 2017;21(2):125-36.
33 Persmark A, Wemrell M, Zettermark S, et al. Precision public health: Mapping socioeconomic disparities in opioid dispensations at Swedish pharmacies by Multilevel Analysis of Individual Heterogeneity and Discriminatory Accuracy (MAIHDA). *PLoS One* 2019;14(8):e0220322 doi:10.1371/journal.pone.0220322
34 Hernandez-Yumar A, Wemrell M, Abasolo Alesson I, et al. Socioeconomic differences in body mass index in Spain: An intersectional multilevel analysis of individual heterogeneity and discriminatory accuracy. *PLoS One* 2018;13(12):e0208624.
35 Lorant V, Deliège D, Eaton W, et al. Socioeconomic inequalities in depression: a meta-analysis. *Am J Epidemiol* 2003;157(2):98-112.
36 Dehlendorf C, Rodriguez MI, Levy K, et al. Disparities in family planning. *Am J Obstet Gynecol* 2010;202(3):214-20.
37 Swedish National Board of Health and Welfare. DRG-koder och text 2019 [DRG codes and text 2019]. Available from: https://www.socialstyrelsen.se/utveckla-verksamhet/e-halsa/klassificering-och-koder/drg/drg-koder-och-definitioner/. [Accessed 2020 Dec 15]
38 Hankivsky O. Women's health, men's health, and gender and health: Implications of intersectionality. *Soc Sci Med* 2012;74(11):1712- 20.
39 Frederick A, Shrifrer D. Race and Disability: From Analogy to Intersectionality. *Sociology of Race and Ethnicity* 2019;5(2):200-14.
40 Knaak S, Mantler E, Szeto A. Mental illness-related stigma in healthcare: Barriers to access and care and evidence-based solutions. *Healthcare Management Forum* 2017;30(2):111-6.
41 Merlo J, Wagner P, Ghith N, et al. An Original Stepwise Multilevel Logistic Regression Analysis of Discriminatory Accuracy: The Case of Neighbourhoods and Health. *PLoS One* 2016;11(4):e0153778.
42 Axelsson Fisk S, Mulinari S, Wemrell M, et al. Chronic Obstructive Pulmonary Disease in Sweden: An intersectional multilevel analysis of individual heterogeneity and discriminatory accuracy. *SSM Popuulationl Health* 2018;4:334-46.
43 Leckie G, Charlton C. Runmlwin - a program to Run the MLwiN multilevel modelling software from within stata. *J Stat Softw* 2013;52(11):1-40.
44 Evans CR, Leckie G, Merlo J. Multilevel versus single-level regression for the analysis of multilevel information: The case of quantitative intersectional analysis. *Soc Sci Med* 2020;245:112499 doi:10.1016/j.socscimed.2019.112499.

For peer review only - http://bmjopen.bmj.com/site/about/guidelines.xhtml

45 Fruzzetti F, Fidecicchi T. Hormonal Contraception and Depression: Updated Evidence and Implications in Clinical Practice. *Clin Drug Investig*. 2020;40(12):1097-1106 doi: 10.1007/s40261-020-00966-8.

46 Segebladh B, Borgström A, Odlind V, et al. Prevalence of psychiatric disorders and premenstrual dysphoric symptoms in patients with experience of adverse mood during treatment with combined oral contraceptives. *Contraception* 2009;79(1):50-5.

47 Borgström A, Odlind V, Ekselius L, et al. Adverse mood effects of combined oral contraceptives in relation to personality traits. *Eur J Obstet Gyn R B* 2008;141(2):127-30.

48 Arrighi HM, Hertz-Picciotto I. The evolving concept of the healthy worker survivor effect *Epidemiology* 1995;5(2):189-96 doi: 10.1097/00001648-199403000-00009.

49 Josefsson A, Wiréhn A-B, Lindberg M, et al. Continuation rates of oral hormonal contraceptives in a cohort of first-time users: a population-based registry study, Sweden 2005–2010. *BMJ Open* 2013;3:e003401 doi:10.1136/bmjopen-2013-003401

50 Carlsten A, Waern M, Ekedahl A, et al. Antidepressant medication and suicide in Sweden. *Pharmacoepidem Dr S* 2001;10(6):525-30.

51 Kalmar S, Szanto K, Rihmer Z, et al. Antidepressant prescription and suicide rates: effect of age and gender. *Suicide Life Threat* 2008;38(4):363-74.

52 Mangrio E, Carlson E, Zdravkovic S. Understanding experiences of the Swedish health care system from the perspective of newly arrived refugees. *BMC Research Notes* 2018;11(1):616.

53 Kirmayer LJ, Narasiah L, Munoz M, et al. Common mental health problems in immigrants and refugees: general approach in primary care. *Can Med Assoc J* 2011;183(12):E959-67.

54 Ditch S, Roberts TA, Hansen S. The influence of health care utilization on the association between hormonal contraception initiation and subsequent depression diagnosis and antidepressant use. *Contraception* 2020;101(4):237-43.

55 Brenner H. Political economy and health. In: Amick III B, Levine S, Tarlov A, et al, eds. Society and health. New York, NY: Oxford university press 1995:211–46.

56 Lynch JW, Kaplan GA, Salonen JT. Why do poor people behave poorly? Variation in adult health behaviours and psychosocial characteristics by stages of the socioeconomic lifecourse. *Soc Sci Med* 1997;44(6):809-19.

57 Collins PY, von Unger H, Armbrister A. Church ladies, good girls, and locas: stigma and the intersection of gender, ethnicity, mental illness, and sexuality in relation to HIV risk. *Soc Sci Med* 2008;67(3):389-97.

58 Honjo K. Social epidemiology: Definition, history, and research examples. *Environ Health Prev* 2004;9(5):193-9.

59 Kubzansky L, Kawachi I. Affective States and Health. In: Berkman L, Kawachi I, eds. Social epidemiology. New York, NY: Oxford University Press 2000:13–41.

60 Krieger N. Epidemiology and the Peoples Health - Theory and Context. Oxford: Oxford University Press 2011:1-381.

61 Krieger N. Epidemiology and social sciences: towards a critical reengagement in the 21st century. *Epidemiol Rev* 2000;22(1):155-63.

62 Link B, Phelan J. Social conditions as fundamental causes of disease. *J Health Soc Behav* 1995(Extra Issue:Forty Years of Medical Sociology):80-94

59 Schatzberg AF. New indications for antidepressants. *J Clin Psychiat* 2000;61(Suppl)11:9-17.

63 Gardarsdottir H, Heerdink ER, van Dijk L, et al. Indications for antidepressant drug prescribing in general practice in the Netherlands. *J Affect Disorders* 2007;98(1-2):109-15.

For peer review only - http://bmjopen.bmj.com/site/about/guidelines.xhtml

64 Merlo J, Mulinari S, Wemrell M, et al. The tyranny of the averages and the indiscriminate use of risk factors in public health: The case of coronary heart disease. *SSM Population Health* 2017;3:684-98 doi:10.1016/j.ssmph.2017.08.005

65 Warner LR. A Best Practices Guide to Intersectional Approaches in Psychological Research. *Sex Roles* 2008;59:454–63.

66 Marmot M. Social justice, epidemiology and health inequalities. *Eur J Epidemiol* 2017;32(7):537-46.

For peer review only

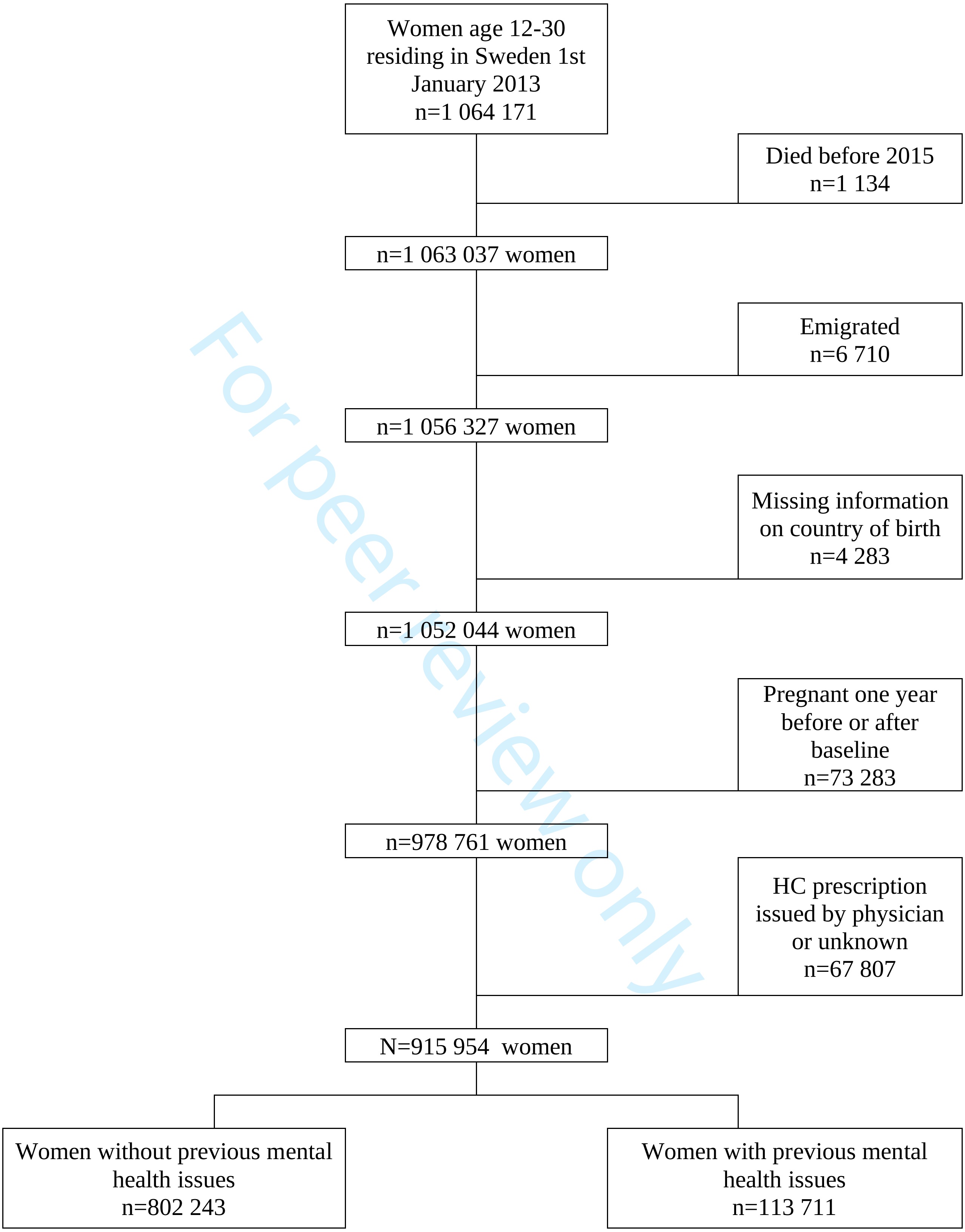

Figure 1. Selection of the study population.

For peer review only

```
* Hormonal Contraception and Antidepressant Use in Sweden: An
Intersectional Multilevel Analysis of Individual Heterogeneity and
Discriminatory Accuracy
* (MAIHDA)
***************************************************************************
clear *
global MLwiN_path "C:\Program Files\MLwiN v3.05\mlwin.exe"
set cformat %9.2f



***************************************************************************
* TABLE 1
***************************************************************************

* Load the data
use "final_mlMENTAL.dta", clear
keep age_cat1 age_cat2 age_cat3 inc1 inc2 inc3 imm pp proportion denom
order age_cat1 age_cat2 age_cat3 inc1 inc2 inc3 imm pp proportion denom

generate percentage = 100*proportion
drop proportion
format %9.2f percentage

generate age_cat = .
replace age_cat = 1 if age_cat1==1
replace age_cat = 2 if age_cat2==1
replace age_cat = 3 if age_cat3==1

generate inc_cat = .
replace inc_cat = 1 if inc1==1
replace inc_cat = 2 if inc2==1
replace inc_cat = 3 if inc3==1

* Results for the table
tabulate pp [fweight = denom]
table pp [fweight = denom], contents(mean percentage )
tabulate age_cat pp [fweight = denom], column nofreq
tabulate inc_cat pp [fweight = denom], column nofreq
tabulate imm pp [fweight = denom], column nofreq



***************************************************************************
* TABLE 2: MODEL 1
***************************************************************************

* Load the data
use "final_mlMENTAL.dta", clear

* IGLS estimation, for MCMC initial values
runmlwin prop cons, ///
  level2(inter: cons) ///
  level1(inter:) ///
  discrete(distribution(binomial) link(logit) denom(denom) mql1) ///
  nopause

* MCMC
runmlwin prop cons, ///
  level2(inter: cons, residuals(u, savechains("m1u.dta",replace))) ///
  level1(inter:) ///
```

```
  discrete(distribution(binomial) link(logit) denom(denom)) ///
  mcmc(burnin(10000) chain(50000) thin(10) savechains("m1b.dta", replace))
///
  initsprevious ///
  nopause

* Level-2 variance
scalar m1sigma2u = [RP2]var(cons)
scalar list m1sigma2u

* Level-1 variance
scalar m1sigma2e = _pi^2/3
scalar list m1sigma2e

* VPC
display "VPC_u = " %9.4f m1sigma2u/(m1sigma2u + m1sigma2e)

* Compress and save the data
compress
save "m1.dta", replace



*---------------------------------------------------------------------------*
* PREPARE FIXED-PART PAREMETER CHAINS
*---------------------------------------------------------------------------*

use "m1b.dta", clear
drop deviance RP2_var_cons_ OD_bcons_1
rename FP1_* b_*
format %9.2f b_*
compress
save "m1b_prepped.dta", replace
isid iteration
codebook iteration, compact



*----------------------------------------------------------------------------*
* PREPARE RANDOM EFFECTS CHAINS
*----------------------------------------------------------------------------*
use "m1u.dta", clear
drop residual idnum
rename value u
format %9.2f u
sort inter iteration
order inter iteration
compress
save "m1u_prepped.dta", replace
isid inter iteration
codebook iteration, compact



*----------------------------------------------------------------------------*
* MERGE DATA, FIXED-PART PARAMETER AND RANDOM EFFECT CHAINS TOGETHER
*----------------------------------------------------------------------------*
use "final_mlMENTAL", clear
count
cross using "m1b_prepped.dta"
count
```

```
merge m:1 inter iteration using "m1u_prepped.dta", nogenerate assert(match)
count
compress
save "m1data_prepped.dta", replace



*------------------------------------------------------------------------*
* ROC
*------------------------------------------------------------------------*
use "m1data_prepped.dta", clear
count
generate p = invlogit(b_cons + u)
gcollapse (mean) p, by(inter num denom)
count
expand denom
sort inter
bysort inter: generate y = (_n<=numerator)
generate prop = denom/_N
generate weight = int(1/prop)
roctab y p [fw=weight]



*------------------------------------------------------------------------*
* TABLE 3
*------------------------------------------------------------------------*
use "m1data_prepped.dta", clear
keep iteration inter age_cat1 age_cat2 age_cat3 inc1 inc2 inc3 imm pp denom
b_cons u
count
generate p = 100*invlogit(b_cons + u)
drop b_cons u
format %9.1f p
drop inter
reshape wide denom p, i(iteration age_cat1 age_cat2 age_cat3 inc1 inc2 inc3
imm) j(pp)
generate denom = denom0 + denom1
drop denom0 denom1
generate pdiff = p1 - p0
gcollapse (mean) p0 p1 pdiff (p2.5) pdifflo=pdiff (p97.5) pdiffhi=pdiff,
by(age_cat1 age_cat2 age_cat3 inc1 inc2 inc3 imm denom)
format %9.1f pdiff pdifflo pdiffhi
order p1 p0 pdiff pdifflo pdiffhi, last
gsort -age_cat1 -age_cat2 -age_cat3 -inc1 -inc2 -inc3 imm



**************************************************************************
* TABLE 2: MODEL 2:
**************************************************************************

* Load the data
use "final_mlMENTAL.dta", clear

* IGLS estimation, for MCMC initial values
runmlwin prop cons age_cat2 age_cat3 inc1 inc2 imm pp, ///
  level2(inter: cons) ///
  level1(inter:) ///
  discrete(distribution(binomial) link(logit) denom(denom) mql1) ///
  nopause
```

```
* MCMC
runmlwin prop cons age_cat2 age_cat3 inc1 inc2 imm  pp, ///
  level2(inter: cons, residuals(u,savechains("m2u.dta",replace))) ///
  level1(inter:) ///
  discrete(distribution(binomial) link(logit) denom(denom)) ///
  mcmc(burnin(10000) chain(50000) thin(10) savechains("m2b.dta", replace))
///
  initsprevious ///
  nopause

* Odds ratios
runmlwin, or

* Level-2 variance
scalar m2sigma2u = [RP2]var(cons)
scalar list m2sigma2u

* Level-1 variance
scalar m2sigma2e = _pi^2/3
scalar list m2sigma2e

* VPC
display "VPC_u = " %9.4f m2sigma2u/(m2sigma2u + m2sigma2e)

* Compress and save the data
compress
save "m2.dta", replace

* PCV
display "PCV = " %9.4f (m2sigma2u - m1sigma2u)/m1sigma2u



*-------------------------------------------------------------------------*
* PREPARE FIXED-PART PAREMETER CHAINS
*-------------------------------------------------------------------------*
use "m2b.dta", clear
drop deviance RP2_var_cons_ OD_bcons_1
rename FP1_* b_*
format %9.2f b_*
compress
save "m2b_prepped.dta", replace
isid iteration
codebook iteration, compact



*-------------------------------------------------------------------------*
* PREPARE inter RANDOM EFFECTS CHAINS
*-------------------------------------------------------------------------*
use "m2u.dta", clear
drop residual idnum
rename value u
format %9.2f u
sort inter iteration
order inter iteration
compress
save "m2u_prepped.dta", replace
isid inter iteration
codebook iteration, compact
```

```
*--------------------------------------------------------------------------*
* MERGE DATA, FIXED-PART PARAMETER AND RANDOM EFFECT CHAINS TOGETHER
*--------------------------------------------------------------------------*
use "final_mlMENTAL", clear
count
cross using "m2b_prepped.dta"
count
merge m:1 inter iteration using "m2u_prepped.dta"
count
save "m2data_prepped.dta", replace



*--------------------------------------------------------------------------*
* ROC
*--------------------------------------------------------------------------*
use "m2data_prepped.dta", clear
count
generate p = invlogit(b_cons + b_age_cat2*age_cat2 + b_age_cat3*age_cat3 +
b_inc1*inc1 + b_inc2*inc2 + b_imm*imm + b_pp*pp)
gcollapse (mean) p, by(inter num denom)
count
expand denom
sort inter
bysort inter: generate y = (_n<=numerator)
generate prop = denom/_N
generate weight = int(1/prop)
roctab y p [fw=weight]



*--------------------------------------------------------------------------*
* TABLE 3
*--------------------------------------------------------------------------*
use "m1data_prepped.dta", clear
keep iteration inter age_cat1 age_cat2 age_cat3 inc1 inc2 inc3 imm pp denom
b_cons u
count
generate p = 100*invlogit(b_cons + u)
drop b_cons u
format %9.1f p
drop inter
reshape wide denom p, i(iteration age_cat1 age_cat2 age_cat3 inc1 inc2 inc3
imm) j(pp)
generate denom = denom0 + denom1
drop denom0 denom1
generate pdiff = p1 - p0
gcollapse (mean) p0 p1 pdiff (p2.5) pdifflo=pdiff (p97.5) pdiffhi=pdiff,
by(age_cat1 age_cat2 age_cat3 inc1 inc2 inc3 imm denom)
format %9.1f pdiff pdifflo pdiffhi
order p1 p0 pdiff pdifflo pdiffhi, last
gsort -age_cat1 -age_cat2 -age_cat3 -inc1 -inc2 -inc3 imm



****************************************************************************
exit
```

```
*****************************************************************************
* Hormonal Contraception and Antidepressant Use in Sweden: An
Intersectional Multilevel Analysis of Individual Heterogeneity and
Discriminatory Accuracy (MAIHDA)

*****************************************************************************
clear *
global MLwiN_path "C:\Program Files\MLwiN v3.05\mlwin.exe"
set cformat %9.2f



******************************************************************************
* TABLE 1
******************************************************************************

* Load the data
use "final_mlNoMENTAL.dta", clear
keep age_cat1 age_cat2 age_cat3 inc1 inc2 inc3 imm pp proportion denom
order age_cat1 age_cat2 age_cat3 inc1 inc2 inc3 imm pp proportion denom

generate percentage = 100*proportion
drop proportion
format %9.2f percentage

generate age_cat = .
replace age_cat = 1 if age_cat1==1
replace age_cat = 2 if age_cat2==1
replace age_cat = 3 if age_cat3==1

generate inc_cat = .
replace inc_cat = 1 if inc1==1
replace inc_cat = 2 if inc2==1
replace inc_cat = 3 if inc3==1

* Results for the table
tabulate pp [fweight = denom]
table pp [fweight = denom], contents(mean percentage )
tabulate age_cat pp [fweight = denom], column nofreq
tabulate inc_cat pp [fweight = denom], column nofreq
tabulate imm pp [fweight = denom], column nofreq



******************************************************************************
* TABLE 2: MODEL 1
******************************************************************************

* Load the data
use "final_mlNoMENTAL.dta", clear

* IGLS estimation, for MCMC initial values
runmlwin prop cons, ///
  level2(inter: cons) ///
  level1(inter:) ///
  discrete(distribution(binomial) link(logit) denom(denom) mql1) ///
  nopause

* MCMC
runmlwin prop cons, ///
  level2(inter: cons, residuals(u, savechains("m1u.dta",replace))) ///
```

```
  level1(inter:) ///
  discrete(distribution(binomial) link(logit) denom(denom)) ///
  mcmc(burnin(10000) chain(50000) thin(10) savechains("m1b.dta", replace))
///
  initsprevious ///
  nopause

* Level-2 variance
scalar m1sigma2u = [RP2]var(cons)
scalar list m1sigma2u

* Level-1 variance
scalar m1sigma2e = _pi^2/3
scalar list m1sigma2e

* VPC
display "VPC_u = " %9.4f m1sigma2u/(m1sigma2u + m1sigma2e)

* Compress and save the data
compress
save "m1.dta", replace



*-------------------------------------------------------------------------*
* PREPARE FIXED-PART PAREMETER CHAINS
*-------------------------------------------------------------------------*

use "m1b.dta", clear
drop deviance RP2_var_cons_ OD_bcons_1
rename FP1_* b_*
format %9.2f b_*
compress
save "m1b_prepped.dta", replace
isid iteration
codebook iteration, compact



*-------------------------------------------------------------------------*
* PREPARE RANDOM EFFECTS CHAINS
*-------------------------------------------------------------------------*
use "m1u.dta", clear
drop residual idnum
rename value u
format %9.2f u
sort inter iteration
order inter iteration
compress
save "m1u_prepped.dta", replace
isid inter iteration
codebook iteration, compact



*-------------------------------------------------------------------------*
* MERGE DATA, FIXED-PART PARAMETER AND RANDOM EFFECT CHAINS TOGETHER
*-------------------------------------------------------------------------*
use "final_mlNoMENTAL", clear
count
cross using "m1b_prepped.dta"
```

```
count
merge m:1 inter iteration using "m1u_prepped.dta", nogenerate assert(match)
count
compress
save "m1data_prepped.dta", replace


*-------------------------------------------------------------------------*
* ROC
*-------------------------------------------------------------------------*
use "m1data_prepped.dta", clear
count
generate p = invlogit(b_cons + u)
gcollapse (mean) p, by(inter num denom)
count
expand denom
sort inter
bysort inter: generate y = (_n<=numerator)
generate prop = denom/_N
generate weight = int(1/prop)
roctab y p [fw=weight]


*-------------------------------------------------------------------------*
* TABLE 3
*-------------------------------------------------------------------------*
use "m1data_prepped.dta", clear
keep iteration inter age_cat1 age_cat2 age_cat3 inc1 inc2 inc3 imm pp denom
b_cons u
count
generate p = 100*invlogit(b_cons + u)
drop b_cons u
format %9.1f p
drop inter
reshape wide denom p, i(iteration age_cat1 age_cat2 age_cat3 inc1 inc2 inc3
imm) j(pp)
generate denom = denom0 + denom1
drop denom0 denom1
generate pdiff = p1 - p0
gcollapse (mean) p0 p1 pdiff (p2.5) pdifflo=pdiff (p97.5) pdiffhi=pdiff,
by(age_cat1 age_cat2 age_cat3 inc1 inc2 inc3 imm denom)
format %9.1f pdiff pdifflo pdiffhi
order p1 p0 pdiff pdifflo pdiffhi, last
gsort -age_cat1 -age_cat2 -age_cat3 -inc1 -inc2 -inc3 imm


***************************************************************************
* TABLE 2: MODEL 2:
***************************************************************************

* Load the data
use "final_mlNoMENTAL.dta", clear

* IGLS estimation, for MCMC initial values
runmlwin prop cons age_cat2 age_cat3 inc1 inc2 imm pp, ///
  level2(inter: cons) ///
  level1(inter:) ///
  discrete(distribution(binomial) link(logit) denom(denom) mql1) ///
```

```
  nopause

* MCMC
runmlwin prop cons age_cat2 age_cat3 inc1 inc2 imm  pp, ///
  level2(inter: cons, residuals(u,savechains("m2u.dta",replace))) ///
  level1(inter:) ///
  discrete(distribution(binomial) link(logit) denom(denom)) ///
  mcmc(burnin(10000) chain(50000) thin(10) savechains("m2b.dta", replace))
///
  initsprevious ///
  nopause

* Odds ratios
runmlwin, or

* Level-2 variance
scalar m2sigma2u = [RP2]var(cons)
scalar list m2sigma2u

* Level-1 variance
scalar m2sigma2e = _pi^2/3
scalar list m2sigma2e

* VPC
display "VPC_u = " %9.4f m2sigma2u/(m2sigma2u + m2sigma2e)

* Compress and save the data
compress
save "m2.dta", replace

* PCV
display "PCV = " %9.4f (m2sigma2u - m1sigma2u)/m1sigma2u



*---------------------------------------------------------------------------*
* PREPARE FIXED-PART PAREMETER CHAINS
*---------------------------------------------------------------------------*
use "m2b.dta", clear
drop deviance RP2_var_cons_ OD_bcons_1
rename FP1_* b_*
format %9.2f b_*
compress
save "m2b_prepped.dta", replace
isid iteration
codebook iteration, compact



*---------------------------------------------------------------------------*
* PREPARE inter RANDOM EFFECTS CHAINS
*---------------------------------------------------------------------------*
use "m2u.dta", clear
drop residual idnum
rename value u
format %9.2f u
sort inter iteration
order inter iteration
compress
save "m2u_prepped.dta", replace
isid inter iteration
```

```
codebook iteration, compact



*-----------------------------------------------------------------------------*
* MERGE DATA, FIXED-PART PARAMETER AND RANDOM EFFECT CHAINS TOGETHER
*-----------------------------------------------------------------------------*
use "final_mlNoMENTAL", clear
count
cross using "m2b_prepped.dta"
count
merge m:1 inter iteration using "m2u_prepped.dta"
count
save "m2data_prepped.dta", replace



*-----------------------------------------------------------------------------*
* ROC
*-----------------------------------------------------------------------------*
use "m2data_prepped.dta", clear
count
generate p = invlogit(b_cons + b_age_cat2*age_cat2 + b_age_cat3*age_cat3 +
b_inc1*inc1 + b_inc2*inc2 + b_imm*imm + b_pp*pp)
gcollapse (mean) p, by(inter num denom)
count
expand denom
sort inter
bysort inter: generate y = (_n<=numerator)
generate prop = denom/_N
generate weight = int(1/prop)
roctab y p [fw=weight]



*-----------------------------------------------------------------------------*
* TABLE 3
*-----------------------------------------------------------------------------*
use "m1data_prepped.dta", clear
keep iteration inter age_cat1 age_cat2 age_cat3 inc1 inc2 inc3 imm pp denom
b_cons u
count
generate p = 100*invlogit(b_cons + u)
drop b_cons u
format %9.1f p
drop inter
reshape wide denom p, i(iteration age_cat1 age_cat2 age_cat3 inc1 inc2 inc3
imm) j(pp)
generate denom = denom0 + denom1
drop denom0 denom1
generate pdiff = p1 - p0
gcollapse (mean) p0 p1 pdiff (p2.5) pdifflo=pdiff (p97.5) pdiffhi=pdiff,
by(age_cat1 age_cat2 age_cat3 inc1 inc2 inc3 imm denom)
format %9.1f pdiff pdifflo pdiffhi
order p1 p0 pdiff pdifflo pdiffhi, last
gsort -age_cat1 -age_cat2 -age_cat3 -inc1 -inc2 -inc3 imm



*******************************************************************************
exit
```

For peer review only

```
pp,imm,inter,age_cat1,age_cat2,age_cat3,inc1,inc2,inc3,proportion,numerator,denom,cons
0,0,12-17 Low income 0 0,1,0,0,1,0,0,.013224002,279,21098,1
1,0,12-17 Low income 0 1,1,0,0,1,0,0,.037408244,265,7084,1
0,1,12-17 Low income 1 0,1,0,0,1,0,0,.0040497542,28,6914,1
1,1,12-17 Low income 1 1,1,0,0,1,0,0,.0096021947,7,729,1
0,0,12-17 Middle income 0 0,1,0,0,0,1,0,.010099272,587,58123,1
1,0,12-17 Middle income 0 1,1,0,0,0,1,0,.030316716,537,17713,1
0,1,12-17 Middle income 1 0,1,0,0,0,1,0,.0056107035,52,9268,1
1,1,12-17 Middle income 1 1,1,0,0,0,1,0,.017814728,15,842,1
0,0,12-17 High income 0 0,1,0,0,0,0,1,.008893352,859,96589,1
1,0,12-17 High income 0 1,1,0,0,0,0,1,.01951286,572,29314,1
0,1,12-17 High income 1 0,1,0,0,0,0,1,.0076045627,30,3945,1
1,1,12-17 High income 1 1,1,0,0,0,0,1,.025718609,17,661,1
0,0,18-23 Low income 0 0,0,1,0,1,0,0,.029676914,530,17859,1
1,0,18-23 Low income 0 1,0,1,0,1,0,0,.034916617,938,26864,1
0,1,18-23 Low income 1 0,0,1,0,1,0,0,.011607248,98,8443,1
1,1,18-23 Low income 1 1,0,1,0,1,0,0,.022702307,62,2731,1
0,0,18-23 Middle income 0 0,0,1,0,0,1,0,.027664155,771,27870,1
1,0,18-23 Middle income 0 1,0,1,0,0,1,0,.0282459,1247,44148,1
0,1,18-23 Middle income 1 0,0,1,0,0,1,0,.011609907,75,6460,1
1,1,18-23 Middle income 1 1,0,1,0,0,1,0,.023316063,54,2316,1
0,0,18-23 High income 0 0,0,1,0,0,0,1,.023347162,1058,45316,1
1,0,18-23 High income 0 1,0,1,0,0,0,1,.022887168,2082,90968,1
0,1,18-23 High income 1 0,0,1,0,0,0,1,.017995911,44,2445,1
1,1,18-23 High income 1 1,0,1,0,0,0,1,.019577537,38,1941,1
0,0,24-30 Low income 0 0,0,0,1,1,0,0,.032189574,2168,67351,1
1,0,24-30 Low income 0 1,0,0,1,1,0,0,.031126546,1954,62776,1
0,1,24-30 Low income 1 0,0,0,1,1,0,0,.013751426,446,32433,1
1,1,24-30 Low income 1 1,0,0,1,1,0,0,.026964672,187,6935,1
0,0,24-30 Middle income 0 0,0,0,1,0,1,0,.030455342,818,26859,1
1,0,24-30 Middle income 0 1,0,0,1,0,1,0,.03591495,652,18154,1
0,1,24-30 Middle income 1 0,0,0,1,0,1,0,.023714487,202,8518,1
1,1,24-30 Middle income 1 1,0,0,1,0,1,0,.027789129,68,2447,1
0,0,24-30 High income 0 0,0,0,1,0,0,1,.025993951,593,22813,1
1,0,24-30 High income 0 1,0,0,1,0,0,1,.024208747,501,20695,1
0,1,24-30 High income 1 0,0,0,1,0,0,1,.023088569,61,2642,1
1,1,24-30 High income 1 1,0,0,1,0,0,1,.019407559,19,979,1
```

For peer review only

```
pp,imm,inter,age_cat1,age_cat2,age_cat3,inc1,inc2,inc3,proportion,numerator,denom,cons
0,0,12-17 Low income 0 0,1,0,0,1,0,0,.22574355,463,2051,1
1,0,12-17 Low income 0 1,1,0,0,1,0,0,.3049593,412,1351,1
0,1,12-17 Low income 1 0,1,0,0,1,0,0,.12383901,40,323,1
1,1,12-17 Low income 1 1,1,0,0,1,0,0,.1891892,21,111,1
0,0,12-17 Middle income 0 0,1,0,0,0,1,0,.23362993,1024,4383,1
1,0,12-17 Middle income 0 1,1,0,0,0,1,0,.31201944,771,2471,1
0,1,12-17 Middle income 1 0,1,0,0,0,1,0,.13422818,60,447,1
1,1,12-17 Middle income 1 1,1,0,0,0,1,0,.18032786,22,122,1
0,0,12-17 High income 0 0,1,0,0,0,0,1,.28093326,1469,5229,1
1,0,12-17 High income 0 1,1,0,0,0,0,1,.34217408,916,2677,1
0,1,12-17 High income 1 0,1,0,0,0,0,1,.18867925,50,265,1
1,1,12-17 High income 1 1,1,0,0,0,0,1,.3018868,32,106,1
0,0,18-23 Low income 0 0,0,1,0,1,0,0,.37809917,2013,5324,1
1,0,18-23 Low income 0 1,0,1,0,1,0,0,.39212543,2201,5613,1
0,1,18-23 Low income 1 0,0,1,0,1,0,0,.19350649,149,770,1
1,1,18-23 Low income 1 1,0,1,0,1,0,0,.28291318,101,357,1
0,0,18-23 Middle income 0 0,0,1,0,0,1,0,.36302635,2164,5961,1
1,0,18-23 Middle income 0 1,0,1,0,0,1,0,.37776819,2627,6954,1
0,1,18-23 Middle income 1 0,0,1,0,0,1,0,.19285715,108,560,1
1,1,18-23 Middle income 1 1,0,1,0,0,1,0,.27112675,77,284,1
0,0,18-23 High income 0 0,0,1,0,0,0,1,.39782199,2959,7438,1
1,0,18-23 High income 0 1,0,1,0,0,0,1,.38269973,3765,9838,1
0,1,18-23 High income 1 0,0,1,0,0,0,1,.25,82,328,1
1,1,18-23 High income 1 1,0,1,0,0,0,1,.27906978,84,301,1
0,0,24-30 Low income 0 0,0,0,1,1,0,0,.49862742,9082,18214,1
1,0,24-30 Low income 0 1,0,0,1,1,0,0,.50085437,5569,11119,1
0,1,24-30 Low income 1 0,0,0,1,1,0,0,.32457545,1013,3121,1
1,1,24-30 Low income 1 1,0,0,1,1,0,0,.37422037,360,962,1
0,0,24-30 Middle income 0 0,0,0,1,0,1,0,.50859779,2869,5641,1
1,0,24-30 Middle income 0 1,0,0,1,0,1,0,.49799198,1488,2988,1
0,1,24-30 Middle income 1 0,0,0,1,0,1,0,.37214136,358,962,1
1,1,24-30 Middle income 1 1,0,0,1,0,1,0,.33590734,87,259,1
0,0,24-30 High income 0 0,0,0,1,0,0,1,.48993289,1971,4023,1
1,0,24-30 High income 0 1,0,0,1,0,0,1,.48666918,1296,2663,1
0,1,24-30 High income 1 0,0,0,1,0,0,1,.37669376,139,369,1
1,1,24-30 High income 1 1,0,0,1,0,0,1,.45238096,57,126,1
```

For peer review only

For peer review only - http://bmjopen.bmj.com/site/about/guidelines.xhtml

Supplementary material 5

Supplementary table, summary statistics. Numbers are percentages (numbers within brackets).

		Hormonal contraception	Mental health issues
Age	12-17	23.2 (63 181)	7.2 (19 536)
	18-23	59.9 (192 315)	13.6 (43 729)
	24-30	40.3 (130 103)	15.6 (50 447)
Income	Low	40.8 (126 632)	15.9 (49 316)
	Middle	38.9 (98 698)	12.2 (31 032)
	High	45.6 (160 269)	9.5 (33 363)
Immigrant background	No	45.1 (363 390)	12.9 (103 938)
	Yes	20.1 (22 209)	8.9 (9 773)

Supplementary table. Percentage of women within each intersectional dimension using hormonal contraceptives and with previous mental health issues.

For peer review only

Supplementary material 6

ATC	Freq.	Percent
G02BA03	12 535	3.25
G02BB	96	0.02
G0BB01	26 022	6.75
G02BB01	48	0.01
G03AA03	4 786	1.24
G03AA07	126 061	32.69
G03AA09	3 227	0.84
G03AA11	15 463	4.01
G03AA12	4 596	13.69
G03AA13	12 329	1.19
G03AA14	5 958	3.20
G03AB	5 958	1.55
G03AB03	8 014	2.08
G03AB04	5 341	1.39
G03AC01	4 249	1.10
G03AC02	2 483	0.64
G03AC06	2 710	0.70
G03AC08	21 284	5.52
G03AC09	77 595	20.12

Supplementary table, frequency table of hormonal contraceptives. Frequency of all included hormonal contraceptives in the final cohort of 915 954 women.

For peer review only

Supplementary material 7

Sensitivity analysis only including women with a recent health care contact (defined as any dispensed prescription or appointment at a hospital in the last 3 years) = 60.46% of the original population

Table 1. Characteristics of the 553 789 women aged 12 - 30 years and residing in Sweden by 1st January 2013 by previous mental health issues and use of hormonal contraceptives. Values are percentages (number of women) if not otherwise indicated.

	Previous mental health issues			
	Yes 19.01 (n = 105 283) Use of Hormonal contraceptives		No 80.99 (n = 448 506) Use of Hormonal contraceptives	
	Yes 42.42 (n = 44657)	No 57.58 (n = 60 626)	Yes 44.41 (n= 199 170)	No 55.59 (n = 249 336)
Antidepressant drugs	41.17 (19 886)	39.77 (26 013)	2.73 (9 215)	1.87 (8 699)
Age				
12-17 years	15.11 (6 747)	20.75 (12 581)	15.63 (31 133)	37.24 (92 846)
18-23 years	48.78 (21 784)	31.29 (18 968)	48.30 (96 200)	22.75 (56 735)
24-30 years	36.11 (16 126)	47.96 (29 077)	36.07 (71 837)	40.01 (99 755)
Income level				
Low	39.70 (17 731)	45.01 (27 286)	33.06 (65 847)	35.08 (87 456)
Middle	27.55 (12 302)	27.85 (16 887)	26.00 (51 776)	29.91 (74 575)
High	32.75 (14 624)	27.14 (16 453)	40.94 (81 547)	35.01 (87 305)
Immigrant background				
No	94.50 (42 200)	88.94 (53 919)	93.76 (186 745)	83.61 (208 465)
Yes	5.50 (2 457)	11.06 (6 707)	6.24 (12 425)	16.39 (40 871)

For peer review only - http://bmjopen.bmj.com/site/about/guidelines.xhtml

Table 2. Results from the Multilevel Analysis of Individual Heterogeneity and Discriminatory Accuracy (MAIHDA) distinguishing between measures of association and measures of variance and discriminatory accuracy. The analyses are stratified by the existence of previous mental issues. Values are point estimations with (95% Confidence Intervals)

	Without metal health issues		With mental health issues	
	Model 1	Model 2	Model 1	Model 2
**Measures of association,** Odds Ratios				
Age				
12-17		Reference		Reference
18-23		1.73 (1.33-2.22)		1.52 (1.33-1.71)
24-30		1.90 (1.48-2.40)		2.58 (2.29-2.91)
Income				
High		Reference		Reference
Middle		1.16 (0.91-1.48)		0.89 (0.79-1.01)
Low		1.17 (0.92-1.55)		0.89 (0.78-1.01)
Immigrant background				
No		Reference		Reference
Yes		0.65 (0.53-0.81)		0.55 (0.49-0.61)
Hormonal contraceptives				
No		Reference		Reference
Yes		1.40 (1.12-1.71)		1.18 (1.06-1.34)
**Measures of variance**				
Variance*	0.224 (0.130-0.372)	0.077 (0.038-0.141)	0.287 (0.174-0.468)	0.017 (0.008-0.033)
VPC	6.38%	2.29%	8.02%	0.51%
PCV		65.67%		94.09%
AUC	0.61 (0.61-0.61)	0.61 (0.61-0.61)	0.64 (0.64-0.64)	0.64 (0.64-0.64)

*Between-strata variance, variance partition coefficient (VPC), proportional change of the variance (PCV), Area under the curve (AUC)

For peer review only - http://bmjopen.bmj.com/site/about/guidelines.xhtml

Table 3. Absolute risk (AR) of antidepressant use, and AR difference (ARD) between user and non-users of hormonal contraceptives but otherwise sharing the same intersectional stratum. The values are calculated from the multilevel analysis of individual heterogeneity and discriminatory accuracy (MAIHDA)

Previous mental health issues	Age (years)	Income level	Immigrant background	Number of women	Use of hormonal contraceptive		
					Yes AR	No AR	Yes-No difference ARD
No	12 – 17	Low	No	14060	4.4	1.8	2.7 (2 - 3.4)
			Yes	3123	1.5	0.8	0.8 (-0.1 - 2)
		Middle	No	37376	3.6	1.3	2.2 (1.8 - 2.6)
			Yes	4543	2.4	1.0	1.4 (0.3 - 2.8)
		High	No	62712	2.4	1.1	1.3 (1 - 1.5)
			Yes	2165	2.8	1.2	1.7 (0.4 - 3.3)
	18 – 23	Low	No	25939	4.2	3.4	0.8 (0.3 - 1.2)
			Yes	5720	2.5	2.0	0.5 (-0.3 - 1.4)
		Middle	No	40241	3.3	3.6	-0.3 (-0.6 - 0.1)
			Yes	4547	2.7	1.8	0.9 (0 - 1.9)
		High	No	74281	2.7	2.9	-0.2 (-0.4 - 0.1)
			Yes	2207	2.0	2.7	-0.6 (-1.9 - 0.5)
	24 – 30	Low	No	83448	3.6	3.7	0 (-0.3 - 0.2)
			Yes	21013	2.9	2.1	0.8 (0.3 - 1.4)
		Middle	No	31818	4.0	3.5	0.5 (0.1 - 0.9)
			Yes	7826	3.0	2.7	0.3 (-0.5 - 1.2)
		High	No	25335	2.9	3.0	-0.1 (-0.5 - 0.3)
			Yes	2152	2.5	2.7	-0.2 (-1.5 - 1.2)
Yes	12 – 17	Low	No	3371	30.1	22.3	7.8 (4.7 - 10.9)
			Yes	429	20.4	13.4	7 (-0.4 - 14.8)
		Middle	No	6787	31.0	23.1	7.9 (5.7 - 10.1)
			Yes	565	19.5	14.3	5.2 (-1.5 - 12.7)
		High	No	7807	33.8	27.7	6.1 (3.9 - 8.2)
			Yes	369	29.7	19.4	10.3 (1.2 - 19.6)
	18 – 23	Low	No	10205	38.8	36.9	1.9 (0.1 - 3.8)
			Yes	1068	28.1	19.0	9.1 (3.5 - 14.6)
		Middle	No	12082	36.6	35.0	1.6 (-0.1 - 3.3)
			Yes	805	27.2	19.1	8.1 (2 - 14.3)
		High	No	15994	37.2	38.5	-1.3 (-2.8 - 0.3)
			Yes	598	26.3	25.4	0.8 (-6.1 - 7.7)
	24 – 30	Low	No	26185	49.2	48.9	0.3 (-0.9 - 1.6)
			Yes	3759	36.0	32.6	3.4 (-0.3 - 7)
		Middle	No	7820	49.2	50.4	-1.3 (-3.6 - 1)
			Yes	1130	31.4	37.2	-5.8 (-12.4 - 0.9)

For peer review only - http://bmjopen.bmj.com/site/about/guidelines.xhtml

For peer review only

High	No	5868	47.8	47.8	0 (-2.6 - 2.6)
	Yes	441	41.3	35.2	6.1 (-3.6 - 16)

For peer review only

For peer review only - http://bmjopen.bmj.com/site/about/guidelines.xhtml

BMJ Open

Population Heterogeneity in Associations Between Hormonal Contraception and Antidepressant Use in Sweden: A Prospective Cohort Study Applying Intersectional Multilevel Analysis of Individual Heterogeneity and Discriminatory Accuracy (MAIHDA)

Journal:	*BMJ Open*
Manuscript ID	bmjopen-2021-049553.R2
Article Type:	Original research
Date Submitted by the Author:	26-Aug-2021

Complete List of Authors:	Zettermark, Sofia; Lund University, Unit for Social Epidemiology, Department of Clinical Sciences, Malmö<br>Khalaf, Kani; Lund University, Unit for Social Epidemiology, Department of Clinical Sciences, Malmö<br>Perez-Vicente, Raquel; Lund University, Unit for Social Epidemiology, Department of Clinical Sciences, Malmö<br>Leckie, George; University of Bristol, Center for Multilevel Modelling, School of Education; Lund University, Unit for Social Epidemiology, Department of Clinical Sciences<br>Mulinari, Diana; Lund University, Department of Gender Studies, Faculty of Social Sciences<br>Merlo, Juan; Lund University, Unit for Social Epidemiology, Department of Clinical Sciences; Region Skane Health Care, Center for Primary Health Care Research, Region Skåne

**Primary Subject Heading**:	Epidemiology
Secondary Subject Heading:	Public health, Mental health, Reproductive medicine
Keywords:	MENTAL HEALTH, Sex steroids & HRT < DIABETES & ENDOCRINOLOGY, SOCIAL MEDICINE, EPIDEMIOLOGY, Depression & mood disorders < PSYCHIATRY

SCHOLARONE™
Manuscripts

I, the Submitting Author has the right to grant and does grant on behalf of all authors of the Work (as defined in the below author licence), an exclusive licence and/or a non-exclusive licence for contributions from authors who are: i) UK Crown employees; ii) where BMJ has agreed a CC-BY licence shall apply, and/or iii) in accordance with the terms applicable for US Federal Government officers or employees acting as part of their official duties; on a worldwide, perpetual, irrevocable, royalty-free basis to BMJ Publishing Group Ltd ("BMJ") its licensees and where the relevant Journal is co-owned by BMJ to the co-owners of the Journal, to publish the Work in this journal and any other BMJ products and to exploit all rights, as set out in our licence.

The Submitting Author accepts and understands that any supply made under these terms is made by BMJ to the Submitting Author unless you are acting as an employee on behalf of your employer or a postgraduate student of an affiliated institution which is paying any applicable article publishing charge ("APC") for Open Access articles. Where the Submitting Author wishes to make the Work available on an Open Access basis (and intends to pay the relevant APC), the terms of reuse of such Open Access shall be governed by a Creative Commons licence – details of these licences and which Creative Commons licence will apply to this Work are set out in our licence referred to above.

Other than as permitted in any relevant BMJ Author's Self Archiving Policies, I confirm this Work has not been accepted for publication elsewhere, is not being considered for publication elsewhere and does not duplicate material already published. I confirm all authors consent to publication of this Work and authorise the granting of this licence.

For peer review only

Population Heterogeneity in Associations Between Hormonal Contraception and Antidepressant Use in Sweden: A Prospective Cohort Study Applying Intersectional Multilevel Analysis of Individual Heterogeneity and Discriminatory Accuracy (MAIHDA)

Sofia Zettermark[1*], Kani Khalaf[1], Raquel Perez-Vicente[1], George Leckie[1,3], Diana Mulinari[2], Juan Merlo[1,4]

[1] Unit for Social Epidemiology, Department of Clinical Sciences, Faculty of Medicine, Lund University, Malmö, Sweden

[2] Department of Gender Studies, Faculty of Social Sciences, Lund University, Lund, Sweden

[3] Center for Multilevel Modelling, School of Education, University of Bristol, Bristol, United Kingdom

[4] Center for Primary Health Care Research, Region Skåne, Malmö, Sweden

*Corresponding author:
Sofia Zettermark
sofia.zettermark@med.lu.se
Unit for Social Epidemiology, Department of Clinical Sciences
Faculty of Medicine, Lund University
Jan Waldenströms gata 35, CRC
214 28, Malmö, Sweden

Word count: 4390

Keywords: Epidemiology, Contraception, Hormonal contraception, Mental health, Multilevel analysis

For peer review only - http://bmjopen.bmj.com/site/about/guidelines.xhtml

For peer review only

ABSTRACT

Objectives From a reproductive justice framework, we aimed to investigate how a possible association between hormonal contraceptive (HC) and anti-depressants use (as a proxy for depression) is distributed across intersectional strata in the population. We aimed to visualize how intersecting power dynamics may operate in combination with HC use to increase or decrease subsequent use of anti-depressants. Our main hypothesis was that the previously observed association between HC and anti-depressants use would vary between strata, being more pronounced in more oppressed intersectional contexts. For this purpose, we applied an intersectional Multilevel Analysis of Individual Heterogeneity and Discriminatory Accuracy (MAIHDA) approach.

Design Observational prospective cohort study using record linkage of national Swedish registers.

Setting The population of Sweden.

Participants All 915 954 women aged 12-30 residing in Sweden 2010, without a recent pregnancy and alive during the individual one-year follow-up.

Primary outcome measure Use of any anti-depressant, meaning being dispensed at least one anti-depressant (ATC N06A) during follow-up.

Results Previously mentally healthy hormonal contraceptive users had an odds ratio of 1.79 for use of anti-depressants compared to non-users, whereas this number was 1.28 for women with previous mental health issues. The highest anti-depressant use were uniformly found in strata with previous mental health issues, with highest usage in women aged 24-30 with no immigrant background, low income, and HC use (51.4%). The largest difference in anti-depressant use between HC users and non-users was found in teenagers, and in adult women of immigrant background with low income. Of the total individual variance in the latent propensity of using antidepressant 9.01% (healthy) and 8.16% (with previous mental health issues) was found at the intersectional stratum level.

Conclusions Our study suggests teenagers and women with immigrant background and low income could be more sensitive to mood effects of HC, a heterogeneity important to consider moving forward.

For peer review only - http://bmjopen.bmj.com/site/about/guidelines.xhtml

STRENGTHS AND LIMITATIONS OF THIS STUDY

- Entire Swedish population of women aged 12-30 included
- Pharmacy dispensing automatically linked to individual personal identification number in Sweden through the Swedish Prescribed Drug Register and thus very reliable
- Intersectional MAHIDA is a fruitful way of epidemiologically investigating heterogeneity within a population while considering individual conditions determined by societal power dimensions such as class, gender and race
- Anti-depressant dispensing is not a perfect proxy for depression
- Registers cannot not measure actual use of any medication

For peer review only

For peer review only - http://bmjopen.bmj.com/site/about/guidelines.xhtml

INTRODUCTION

In recent years, attention in the medical community has increasingly been drawn towards depression and other adverse effects on mood related to use of hormonal contraception (HC).(1, 2) Discontinuation rates are high, with mood disturbances or depression being one of the most common complaints.(3-5) Two large epidemiological studies, one in Denmark and the other performed in Sweden, have recently shown a higher risk of anti-depressants and psychotropic drugs use in adolescent users of HC.(6, 7) Randomized controlled trials are rare, but suggest a negative influence of HC on well-being and sexual function,(8, 9) as well as evidence of HC modulating brain activity with subsequent mood alterations in some women.(10, 11) Even though oestrogen and progesterone are known to affect mood,(12) the growing body of evidence in this field is contradictory, with recent reviews concluding that both protective and negative effects of HC on mood exist and more research is needed.(13-16) Despite this uncertainty, many scholars agree that certain subgroups of women seem more vulnerable to psychological side effects of HC than others, particularly teenagers and women with previous mental health issues.(10, 13, 17-20) A call for further investigation into these vulnerable subgroups has been made.(14)

A fruitful way of epidemiologically investigating heterogeneity within a population while considering individual conditions determined by societal power dimensions such as class, gender and race has been developed through intersectional theory in recent years.(21-26) Intersectionality theory was first articulated by Black feminist scholars as a way of understanding how an individual inhabits and is formed by more than one social relation such as gender, "race" or class, and how these classification systems interconnect to create specific contexts of oppression or privilege.(27, 28) These categorizations should not be seen as individual "risky" identities, but as the social, political and economic contextual conditions that outline our lives through structural inequalities.(29) Reproductive justice is a theoretical framework that builds upon intersectionality and centres diverse groups of unprivileged women's reproductive experiences to recognize that societal context and differing resources available shape reproductive health.(30) Applying a reproductive justice framework, it becomes clear that we need to take notice of disparate sociocultural contexts and interlocking power dimensions to understand different patterns of usage as well as possible diverse responses to HC.(31, 32)

To operationalize an intersectional mapping of heterogeneity in use of anti-depressants in relation to HC on a population level, we used a multilevel analysis of individual heterogeneity and discriminatory accuracy (MAIHDA).(21-23, 33, 34) We created

For peer review only - http://bmjopen.bmj.com/site/about/guidelines.xhtml

intersectional strata based on previous literature showing that age, socioeconomic position, and previous mental illness are relevant intersecting dimensions in understanding the relation between HC and depression.(17, 20, 35, 36)

We conceptualise the intersectional strata as social contexts rather than static individual traits, thereby visualising how intersecting power dynamics can act in combination with HC to predispose for depressive mood. Our main hypothesis was that the previously observed association between HC and use of anti-depressants would vary between strata and that this association would be more pronounced in more oppressed intersectional contexts. We investigate this hypothesis on the whole population of women susceptible to HC use in Sweden.

METHOD

Databases and study population

After allowance from the Swedish Ethical Authority and the data safety committees from Statistics Sweden and the Swedish National Board of Health and Welfare, we obtained a database created by record linkage of several nationwide registers administered by Statistics Sweden (the Swedish Population Register and the Longitudinal Integration Database for Health Insurance and Labour Market Studies, LISA) and the Swedish National Board of Health and Welfare (National Patient Register, the Swedish Prescribed Drug Register (SPDR) and the Cause of Death Register). The Swedish authorities linked the registries using a unique personal identification number, but the database was anonymized before delivering it to us. We defined an initial cohort containing all 1,064,171 women aged 12 - 30 years residing in Sweden 1st January 2010 and obtained individual level data on medication use from SPDR, which contain all dispensed drug prescriptions at Swedish pharmacies since 2006.

Every woman was assigned an individual baseline date, defined by the first dispensed prescription of an HC drug between 1 January 2010 and 31 December 2014 after 12 years of age, and was then followed for one year after her individual baseline date. A woman obtaining her first prescription 1 of September 2013 was therefore followed to the 1 of September 2014. For non-users of HC the baseline date could not be based on a HC-prescription and was therefore assigned, to 1st of July 2012 for all adults, but later for some of the younger girls turning 12 during our period of investigation. This means all non-users had been true non-users for at least 1.5 years before their follow-up started (1 January 2010 to 1 July 2012) but also continued to be non-users all the way to 31 December 2014. From the individual baseline date, the women were followed for one year to find out if a prescription of

For peer review only - http://bmjopen.bmj.com/site/about/guidelines.xhtml

an antidepressant was dispensed. Data was also collected on psychiatric disorders and psychotropic drug use in the past three years (see Assessment of variables). After excluding women with incomplete follow-up time due to death, emigration, missing information on country of birth, and pregnancies one year before and after the baseline as well as, the final database consisted of 915 952 women. This database was divided into two cohorts according to the presence or absence of previous mental health issues, see Figure 1.

Assessment of variables

Users of HC were defined as any women who, according the SPDR, filled a prescription of HC (Anatomical Therapeutical Chemical (ATC) classification system codes G02B, G03AA-C) between 1 January 2010 and 31 December 2014, while non-users did not have a prescription filled during the same period. Emergency contraception (G03AD) that are mainly bought over the counter in Sweden was excluded. The majority of HC prescriptions are acquired via midwifes in Sweden (86.0% in our original cohort), whom can only prescribe HC for contraceptive purposes. Physicians, most often gynecologists, can also can prescribe HC for other purposes such as in response to bleeding disturbances or endometriosis. Since these indications could confound our results, we excluded women with physician-issued prescriptions, see Figure 1. HC prescriptions can be dispensed by pharmacies annually or every three months.

Anti-depressant use, the outcome of our study, was defined, according to the SPDR, as being dispensed at least one prescription of antidepressants (ATC: N06A) during the individual one-year follow-up.

Previous mental health issues were defined as having any psychiatric disorder diagnosed at a hospital (ICD: F00-F99) or a dispensed prescription of a psychotropic drug (ATC: N05A, N05B, N06A) in the past three years.

Pregnancies one year previous to baseline and during follow-up were identified according to the 2019 version of the Nordic Diagnosis-Related Group classification (NordDRG), Major Diagnostic Categories codes M14 for pregnancy, delivery and post-partum care.(37)

We used family level data on income as of 31 December 2010 from Statistics Sweden's LISA. Individualized disposable family income was calculated by dividing the total disposable income of the family by the number of family members, taking into account the different consumption weights of adults and children determined by Statistics Sweden. Thereafter, we created three categories (i.e., low, medium, and high) of income using tertile

For peer review only - http://bmjopen.bmj.com/site/about/guidelines.xhtml

cut-offs based on the total Swedish population aged 18 - 80 years. We considered the high-income category as the reference in the comparisons.

We defined immigrant status at the family level as no family member >18 years of age born in Sweden, since understanding of and access to institutions such as health care differ depending on social position such as it is constructed by the power dimensions of race/immigration, as well as the experience of xenophobia. This variable should therefore be considered as an effort to capture a social position affecting possibilities and life trajectories rather than an essentialist view of otherness.We categorized age at the individual baseline into the following groups: 12 to 17, 18 to 23, and 24 to 30 years to capture age specific conditions of adolescents, young adults, and adult women.

Intersectional Strata

Within each cohort stratified by previous mental health issues, we generated 36 intersectional strata by combining three categories of age, three categories of income, two categories of immigrant background, and two categories of HC use. Mental health issues can be considered as a valid category of intersectional investigation in a society that considers an able body and mind vital, in other words relating to the power dimension of able-bodiedness,(38, 39). Mental health issues were also included in the analysis since they are a strong determinant of antidepressant use that needs to be addressed. We could consider that over and above individual characteristics, mental illness-related stigma may condition inequities in health care.(40) As with gender or income, able-bodiedness concerning mental health can therefore be conceptualized as a contextual dimension when defining intersectional strata.

Statistical analysis

We performed an intersectional MAIHDA with individual women at the first level of analysis and the 36 intersectional strata at the second level, stratified by previous mental health issues (See Supplementary material 1-4). The use of antidepressants in the population was thus analysed through two successive multilevel logistic regression models distinguishing between measures of association and measures of variance and discriminatory accuracy.

Model 1

The first model included only an intercept and a random effect for the intersectional strata with no covariates. In this model 1 we first (i) performed a simple analysis of components of variance and calculated the Variance Partition Coefficient (VPC). That is, the share

For peer review only - http://bmjopen.bmj.com/site/about/guidelines.xhtml

(expressed as a percentage) of the total individual variance in the latent propensity of antidepressant use that is at the intersectional strata level. In this simple model, the VPC correspond with the Intraclass Correlation Coefficient (ICC) which informs on the clustering of antidepressant use within intersectional strata. The VPC values extend from 0 to 100%. Second, (ii) we calculate the stratum-specific absolute usage of anti-depressants and their 95% credible intervals (CI) by transformation of the information from the logistic regression to the probability scale. We used this information to map the user heterogeneity across the intersectional strata. Then, (iii) using these stratum-specific predictions, we calculated the Area Under the receiver operator characteristics Curve (AUC). The AUC informs on the accuracy of the intersectional strata information for discriminating those women who used antidepressants from those who did not. The AUC values extend from 0.5 to 1, where 0.5 represent absence of accuracy and 1 represents total accuracy. Both the VPC and the AUC in model 1 can be interpreted as measures of discriminatory accuracy,(41) and inform on the magnitude of the general intersectional effects. The higher the VPC and AUC values, the higher the influence of the intersectional context on individual use of antidepressants. Finally, (iv) we calculated the difference in anti-depressant use and 95% CI between similar pairs of strata differing only on the use of HC. This represents the stratum specific association between HC and antidepressant use.

Model 2 or fixed main effects model

This model includes the fixed, main effects of all the intersectional dimensions (i.e., age, income, immigrant background, and HC use) used to define the intersectional strata. In model 2 we quantified, (i) the association between the intersectional dimensions and use of antidepressants as expressed by odds ratio (OR) and 95% CI. We also to calculate (ii) the Proportional Change in the Variance (PCV). The PCV measures the overall proportion of strata variance of model 1 explained by the specific intersectional dimensions. Since model 2 contains all the variables used to construct the intersectional strata as main effects, it should explain all the strata variance (i.e., PCV= 100%). If this is not the case, the remaining between strata variance would be due to the existence of multiplicative interaction of effects between the intersectional dimensions defining the strata.(22, 42)

The AUCs of the models 1 and 2 are expected to be the same because model 2 only decomposes the stratum-specific predicted probabilities obtained in model 1 into fixed and random effect components and their sum equals the prediction obtained only by random effects in model 1.

For peer review only - http://bmjopen.bmj.com/site/about/guidelines.xhtml

We ran the models using MLwiN 3.00 by calling it from within Stata 14.1 using the *runmlwin* command.(43) The estimations were performed using Markov chain Monte Carlo (MCMC) methods. All points estimations and their 95% credible intervals were based on the parameter and random effect chains obtained from the MCMC estimation. See elsewhere for further information on the statistical MAIHDA analysis including Stata commands,(33, 42) and discussion on the theory and methodological approach.(22, 44)

Patient and Public Involvement statement

The research was developed with a grassroot perspective in mind, whereby women's experiences of use of hormonal contraception inspired and informed the choice of research area and research questions. The anonymised data and scope of the study, including around 1 million women, prohibited direct patent involvement.

RESULTS

Characteristics of the population

The selection of the study population is shown in Figure 1. Out of the 915 952 women 12.4% (n = 113 711) had previous mental health issues. Mean age was somewhat older for women with previous mental health issues (22.5 years; SD 4.8) than for those without such concerns (20.8 years; SD 5.3). Supplementary material 5 shows pooled statistics for usage of previous mental health issues and HC use, while Supplementary material 6 displays a frequency table over all included HC. Table 1 displays the baseline characteristics of the population by previous mental health issues and use of hormonal contraceptives.

Table 1. Characteristics of the 915 954 women aged 12 - 30 years by previous mental health issues and use of hormonal contraceptives. Values are percentages (number of women in parenthesis).

	Previous mental health issues			
	Yes 12.4 (113 711)		No 87.6 (802 243)	
	Use of HC		Use of HC	
	Yes 42.5 (48 302)	No 57.5 (65 409)	Yes 42.0 (337 297)	No 58.0 (464 946)
Antidepressant during follow-up	41.2 (19 886)	39.8 (26 013)	2.7 (9 215)	1.9 (8 699)
Age				
12-17 years	14.2 (6 838)	19.4 (12 698)	16.7 (56 343)	42.1 (195 937)
18-23 years	48.3 (23 347)	31.2 (20 381)	50.1 (168 968)	23.3 (108 939)

For peer review only - http://bmjopen.bmj.com/site/about/guidelines.xhtml

24-30 years	37.5 (18 117)	49.4 (32 330)	33.2 (11 986)	34.6 (160 616)
Income level				
Low inc.	40.4 (19 513)	45.6 (29 803)	31.8 (107 119)	33.1 (154 098)
Medium inc.	27.1 (13 078)	27.5 (17 954)	25.4 (85 620)	29.5 (137 098)
High inc.	32.5 (15 711)	27.0 (17 652)	42.9 (144 558)	37.4 (173 750)
Immigrant background				
None	94.6 (45 674)	89.1 (58 264)	94.2 (317 716)	82.6 (383 878)
Yes	5.4 (2 628)	10.9 (7 145)	5.8 (19 581)	17.4 (81 068)

The share of HC users was very similar in healthy women and those with previous mental health issues, 42.0% and 42.5%, respectively. Anti-depressants were dispensed to 2.7% of HC users compared to 1.9% of non-users among healthy women during follow-up. For women with previous mental health issues, 41.2% of HC users and 39.8% of non-users dispensed an anti-depressant prescription. The income levels were generally higher among women without mental health issues, and HC users were somewhat more affluent in both cohorts.

Results from the MAIHDA

Table 2 shows the results from the MAIHDA distinguishing between measures of association and measures of variance and discriminatory accuracy.

For peer review only

Table 2. Results from the Multilevel Analysis of Individual Heterogeneity and Discriminatory Accuracy (MAIHDA) distinguishing between measures of association (Odds Ratios) and measures of variance and

For peer review only - http://bmjopen.bmj.com/site/about/guidelines.xhtml

discriminatory accuracy. The analyses are stratified by the existence of previous mental issues. Values are point estimations (with 95% credible intervals) or percentages where indicated.

	Without metal health issues		With mental health issues	
	Model 1	Model 2	Model 1	Model 2
**Measures of association**				
Age				
12-17 years		Reference		Reference
18-23 years		1.78 (1.36-2.42)		1.57 (1.38-1.76)
24-30 years		2.09 (1.65-2.70)		2.66 (2.36-3.00)
Income				
High inc.		Reference		Reference
Medium inc.		1.05 (0.78-1.37)		0.87 (0.77-0.98)
Low inc.		1.10 (0.81-1.41)		0.87 (0.77-0.98)
Immigrant background				
None		Reference		Reference
Yes		0.63 (0.49-0.79)		0.55 (0.49-0.61)
Hormonal contraception				
No		Reference		Reference
Yes		1.62 (1.34-2.06)		1.19 (1.08-1.31)
**Measures of variance and discriminatory accuracy***				
Variance	0.30 (0.18-0.50)	0.10 (0.06-0.18)	0.29 (0.18-0.49)	0.02 (0.01-0.03)
VPC	8.45%	3.02%	8.18%	0.49%
PCV		66.29%		94.48%
AUC	0.62 (0.62-0.62)	0.62 (0.62-0.62)	0.64 (0.64-0.64)	0.64 (0.64-0.64)

*Between-strata variance, variance partition coefficient (VPC), proportional change of the variance (PCV), Area under the curve (AUC)

Model 1 indicates that 8.45% (without mental health issues) and 8.18% (with previous mental health issues) of the total individual variance in the latent propensity of using antidepressant is at the intersectional strata level. These VPCs correspond with AUC values of 0.62 and 0.64 respectively. Both measures suggest the existence of a moderate intersectional effect. The PCV was high in both groups, but especially so in the group with previous mental health issues, meaning the intersectional dimensions or main effects explain more of the inter-strata variance for these women. Model 2 shows that HC was associated with increased usage of antidepressants after adjustment for all other intersectional dimensions. This result was seen within both cohorts, but more strongly so in women without previous mental health issues

For peer review only - http://bmjopen.bmj.com/site/about/guidelines.xhtml

(OR 1.62 compared to 1.19). Finally, the VPC in model 2 was very small (3.02% and 0.49% respectively) but did not vanish. This finding means that while the intersectional strata effect was mainly due the additive effect of variables defining the strata, a small component due to interaction of effects could also be detected.

Heterogeneity concerning antidepressant use in our cohort

Women with previous mental health issues had a much higher usage of antidepressants than women without such issues, but the association with HC use nonetheless varied across the other intersectional dimensions. Table 3 show the stratum-specific incidence rates for antidepressant use and 95% CI obtained in model 1.

Table 3. Distribution of antidepressant use between different intersectional strata, and difference in usage between user and non-users of hormonal contraceptives but otherwise sharing the same intersectional stratum. The values are calculated from the multilevel analysis of individual heterogeneity and discriminatory accuracy (MAIHDA). Numbers are percentages.

Previous mental health issues	Age (years)	Income level	Immigrant background	Number of women	Use of hormonal contraceptives (%)		
					Yes	No	Yes-No difference
No	12 – 17	Low	No	28182	3.7	1.3	**2.4 (1.9 , 2.8)**
			Yes	7643	1.2	0.5	**0.7 (0.1 , 1.5)**
		Middle	No	75836	3.0	1.0	**2.0 (1.8 , 2.3)**
			Yes	10110	1.8	0.6	**1.2 (0.5 , 2.1)**
		High	No	125903	2.0	0.9	**1.1 (0.9 , 1.2)**
			Yes	4606	2.5	0.8	**1.6 (0.6 , 2.8)**
	18 – 23	Low	No	44723	3.5	3.0	**0.5 (0.2 , 0.9)**
			Yes	11174	2.3	1.2	**1.1 (0.5 , 1.7)**
		Middle	No	72018	2.8	2.8	0.1 (-0.2 , 0.3)
			Yes	8776	2.3	1.2	**1.1 (0.5 , 1.8)**
		High	No	136284	2.3	2.3	0 (-0.2 , 0.1)
			Yes	4386	2.0	1.8	**0.2 (-0.6 , 0.9)**
	24 – 30	Low	No	130127	3.1	3.2	-0.1 (-0.3 , 0.1)
			Yes	39368	2.7	1.4	**1.3 (0.9 , 1.7)**
		Middle	No	45013	3.6	3.0	**0.5 (0.2 , 0.9)**
			Yes	10965	2.7	2.4	0.4 (-0.3 , 1.1)
		High	No	43508	2.4	2.6	-0.2 (-0.5 , 0.1)
			Yes	3621	1.9	2.3	-0.3 (-1.3 , 0.7)
Yes	12 – 17	Low	No	3402	30.5	22.7	**7.8 (4.7 , 10.8)**
			Yes	434	20.8	13.7	7.1 (-0.3 , 15.1)
		Middle	No	6854	31.2	23.4	**7.8 (5.6 , 10.1)**

For peer review only

		Yes	569	19.9	14.2	5.7 (-1.2 , 13.1)
	High	No	7906	34.2	28.1	**6.1 (3.9 , 8.3)**
		Yes	371	30.4	19.8	**10.6 (1.4 , 19.9)**
18 – 23	Low	No	10937	39.2	37.8	1.4 (-0.4 , 3.2)
		Yes	1127	28.5	19.7	**8.8 (3.4 , 14.4)**
	Middle	No	12915	37.8	36.3	1.5 (-0.2 , 3.1)
		Yes	844	27.4	19.7	**7.7 (1.9 , 13.7)**
	High	No	17276	38.3	39.8	-1.5 (-3 , 0)
		Yes	629	28.1	25.4	2.8 (-4 , 9.4)
24 – 30	Low	No	29333	50.1	49.9	0.2 (-1 , 1.4)
		Yes	4083	37.3	32.4	**4.9 (1.5 , 8.4)**
	Middle	No	8629	49.7	50.8	-1.1 (-3.4 , 1.1)
		Yes	1221	33.5	37.1	-3.6 (-10 , 2.6)
	High	No	6686	48.5	48.9	-0.4 (-2.9 , 2)
		Yes	495	43.7	37.5	6.3 (-3.2 , 15.8)

The highest use of anti-depressants were observed in non-immigrant women, aged 24-30, with previous mental health issues, using HC and with low income (50.1%). The lowest usage were found in teenagers without previous mental health issues and no HC use, especially in the strata of immigrant girls from low (0.50%) and middle-income (0.60%) households.

Heterogeneity concerning the association between hormonal contraceptive and antidepressant use

Overall, the propensity to use antidepressants was consistently higher in HC users compared to non-users in younger women between 12 and 17 years of age, both without previous mental health issues (0.7 – 2.4 percentage points), and with a mental health history (5.7 – 7.8 percentage points) with the magnitude being higher in the latter group. However, the 95% credible intervals were broad since the number of individuals was relatively small in these latter strata. Table 3 gives detailed information on these associations. In adolescents the tendency was that an immigrant background lowered the use of anti-depressants, while the opposite was true for adult women, where a positive association between HC use and later antidepressant use was mainly found in women with low income and immigrant background, again with higher magnitudes in women with previous mental health issues. The association between HC and antidepressant use was smaller in adult women native to Sweden no matter their income, and completely disappeared in adult women with high income regardless of immigrant background.

For peer review only - http://bmjopen.bmj.com/site/about/guidelines.xhtml

DISCUSSION

The main hypothesis of our study was that the previously observed association between HC and antidepressant use, mainly seen in adolescent girls(6-9, 17, 45), would be modified by the intersectional context of the women, being more pronounced in more oppressed intersectional contexts. We confirmed that subsequent use of anti-depressants after an HC prescription compared to non-users of HC within the same intersectional context was heterogenous across intersectional strata pairs. As hypothesized, the difference in propensity to use anti-depressants was more pronounced in more oppressed intersectional contexts like those composed by immigrant, low-income women with previous mental issues. That is, the use of antidepressants and to some extent the difference in use between HC users and non-users varied mainly depending on previous mental health issues, but the HC-antidepressant association was considerably modified across pair of strata with other characteristics equal but where HC use and non-use differed, in both cohorts. Aside from adolescent girls, low and middle income adult women with immigrant background had a more pronounced difference in propensity for using anti-depressants, while adult women without immigrant background had both the lowest anti-depressant use and a low grade of modification by HC use.

Independently of previous mental health issues, the propensity for using anti-depressants was consistently higher for HC users than for non-users in teenagers aged 12-17, a result aligned with previous studies that has found a heterogeneous response with regard to both age and other factors.(6, 7, 17, 18, 20, 45-47) As discussed in a previous paper, this higher risk for adolescents could be due to a *selective discontinuation bias,*(7) a development of the *healthy worker survivor effect,* describing how bias is introduced through a continuous selection where those staying in the workforce are healthier than those who leave.(48) Women who experience a negative influence of HC on psychological health might discontinue treatment in early ages, while those without symptoms continued on HC into adulthood, creating this age-dependent *selective discontinuation bias*. This could explain why the observed association between HC and adverse mental health outcomes are stronger in adolescents. Most Swedish women do however continue their HC treatment with the same method.(49) A previous study found that new users of HC has a higher risk of obtaining anti-depressants within the first six months of HC use than continuous users.(6) To address this possible bias we ran two different sensitivity analyses differentiating between women who filed a first prescription of an HC for the first time during the study period (26.2% of HC

For peer review only - http://bmjopen.bmj.com/site/about/guidelines.xhtml

users) and those that had a repeat prescription. In our cohort the association between HC use and subsequent anti-depressant use was very similar in new and continuous users, but slightly higher among new users, as expected (OR 1.52 and 1.45, respectively, with overlapping 95% confidence intervals). We then excluded all women with HC use any time during 5 years before baseline, thus including using only new users of HC during baseline and never-users as reference group (n = 532 543) and reran the analysis. The association between HC use and subsequent antidepressant use became somewhat stronger in women without mental health issues (OR 1.86) and the VPC also increased. The pattern of antidepressant use in the intersectional strata stayed the same, but the confidence intervals increased since the number of women included was smaller, see Supplementary material 7.

As expected, among adult women the overall propensity for using anti-depressants was higher, as it is known that anti-depressant use increases by age,(50,51) and the difference between HC users and non-users was smaller. Women native to Sweden had a higher propensity for using anti-depressants, but this was moderated by HC exposure to a lower extent than for immigrant women. In adult women native to Sweden, HC use gave no increase of antidepressant use among those with high income. The lower utilization of anti-depressants does not necessarily mean that immigrant women are healthier, since earlier studies have found immigrants utilize healthcare to a lesser extent, even though the need is pronounced, with reasons including discrimination.(52,53) A recent study found that adjustement for health care access eliminatied the association between HC initiation and subsequent anti-depressant use in a US population.(54) Although the health care system is different in Sweden and visits to midwifes for contraceptive purposes free, we conducted a sensitivity analysis including only women who had accessed health care within the last three years to adress this. Using only care-accessors as the reference group did not change our results in any substansive way, see Supplementary material 8 .

Intersectional considerations

The big difference in anti-depressant consumption depending on HC use for lower income immigrant women could be interpreted as the intersectional contexts embodied by these women are more susceptible to the potential detrimental effect of HC on mood. The interrelating negative consequences of low income as a proxy for class or social position, gender and xenophobia may accumulate over the life course and lead to a higher vulnerability to exposures that predispose for antidepressant use later in life,(55-57) whereas this diverse vulnerability to HC exposure might not be visible in teenagers. Social experiences can vary

For peer review only - http://bmjopen.bmj.com/site/about/guidelines.xhtml

depending on for example social position, which in turn impact psychological development, mood and cognition, thus influencing health.(58, 59) In understanding how HC can impact women's mental health differently, both possible individual biological predispositions and social settings need to be investigated, since the emotional response to HC is influenced by context.(32) In other words, the interlocking power axes that create oppression could predispose women already under structural burdens for adverse mental health reactions when using HC. The fact that adult women native to Sweden were almost unaffected by HC use, could strengthen this suggestion. Without the intersectional strata this disparity would not have been so easily identified and visualized.

Focusing on women whose lives are affected by several interlocking power dimensions such as low social position and xenophobia is fundamental to achieving reproductive justice.(30) Nonetheless, our intersectional strata should not be considered static categories of inherently "risky identities" but must be interpreted as context specific vulnerabilities of women within certain interlocking positions, constituted in relation to power dynamics created by unequal schemes such as the economic system.(25, 29) It is likely that in other contexts, other groups could be more vulnerable. It is also important to remember that the purpose of HC most commonly is protection against unwanted pregnancy, a situation that if it arises in itself can have negative mental health effects. In identifying the underlying power systems creating these intersectional categories and acknowledging their constant movement and changing dynamics on a societal level, it furthermore becomes possible to address these inequalities through social change.

In this study, we have combined a classical epidemiological approach of exposure to HC and an intersectional MAHIDA to create a novel understanding of how intersecting power dynamics could create particular vulnerabilities to this specific exposure. Because of our study design, where women are followed for one year after a dispensed prescription of HC, it is more theoretically coherent to view use of HC as an exposure rather than a component of the intersectional strata. However, it is possible to within our approach view HC use as a socio-contextual factor that captures certain living conditions (for example more likely to be sexually active or in a heterosexual relationship), which somewhat changes the interpretation of the results. This epistemological tension is not necessarily a limitation, but could enrich the dialogue in social epidemiology on whether it is possible to separate contextual factors from "pure" exposure.(60-62)

Limitations

For peer review only - http://bmjopen.bmj.com/site/about/guidelines.xhtml

The findings from this study must be interpreted in the context of its limitations. The SPDR has highly reliable data on dispensed prescriptions but cannot measure the actual use of dispensed medications. Whether the women was exposed to HC treatment during her entire follow-up is thus not possible to determine with our method, although previous Swedish data suggest continuation rates for any HC after 6 months are almost 90%.(47) Our methodology does furthermore not allow for differentiation between new users and continuous users of HC. Previous studies has shown an increased risk for depression in new users,(6) which could mean we underestimate the associations when also including continuous users. Nevertheless, a sensitivity analysis (see Supplementary material 7) showed that the pattern of antidepressant use and heterogeneity between groups that the MAIHDA shows remain the same when including only new users. Combining MAIHDA with a survival analysis would possibly address this issue better and could be considered in the future. Use of anti-depressants can be considered a proxy for depression, but anti-depressants are also prescribed for other reasons than depression, including generalized anxiety disorder, obsessive-compulsive disorder and panic disorder.(63) Therefore it is not a perfect proxy of depression but may be a more general indication of impaired mental health.(64) However, out of all women with potentially unfavorable mental health effects from HC, only a subset would have symptoms severe enough to get an anti-depressant prescription, leading instead to many missed cases. Since the outcome is rather common, the risk of underestimation is further enhanced and the true risk of adverse mental health effects could be higher.

As in any observational study, ours only allows for measurements of associations and cannot determine causation. Furthermore, apparently strong average associations do not necessarily convey a high discriminatory accuracy (see elsewhere for a short review and discussion).(65) Nevertheless, since our analysis yielded a moderate accuracy (i.e., AUC=0.6), the intersectional strata do matter for the propensity to use antidepressants. A consideration in every quantitative intersectional study is the basis for creating intersectional categories, since comprehensive information on background and lived experiences are lacking and the categories are created based on available but crude proxies such as income level. For example, in our study the group of women with immigrant background was very heterogenous, so we cannot exclude that the increased antidepressant use is located on more specific country of birth categories. There is an ongoing debate whether these crude categorizations are feasible, and extra caution should be taken when investigating emerging intersectional categories rather than established ones.(66)

For peer review only - http://bmjopen.bmj.com/site/about/guidelines.xhtml

Conclusion

It is important to recognise intersectional perspectives and interacting axes of oppression to tailor better public health interventions, as well as acknowledging the experiences of oppressed women to reach reproductive and social justice. (29, 66) Our intersectional MAIHDA methodology operationalizes this idea by providing information on the discriminatory accuracy of the contexts that define the intersectional strata. It highlights the need to consider disadvantages consisting of several interlocking structural dimensions such as income/class, age and immigration to better understand how HC might predispose certain women, mainly teenagers and low-income women with immigrant background, for depression. These vulnerabilities are based in inequalities that are not static, but structurally created and therefore possible to redeem.

Figure 1. Selection of the study population.

Acknowledgements

A previous version of this study was presented as a poster at the Gynecological Endocrinology, the 19th World Congress in December 2020. We thank all colleagues at the Unit for Social epidemiology, Lund university, for valuable discussions.

Ethical statement

The database was approved by the Regional Ethical Review Board in Lund, Sweden, the Data Safety Board at Statistics Sweden and the National Board of Health and Welfare (Dnr: 2014/856, 2015/341).

Funding statement

This work was supported by The Swedish Research Council (Vetenskapsrådet) grant number: [2017-01321] https://www.swecris.se/betasearch/details/project/201701321VR

Competing interests

None declared.

For peer review only - http://bmjopen.bmj.com/site/about/guidelines.xhtml

Data sharing statement

Public access to the data is restricted by the Swedish Authorities (Public Access to Information and Secrecy Act; http://www.government.se/information-material/2009/09/public-access-to-information-and-secrecy-act/) but data can be made available for researchers after a special review that includes approval of the research project by both an Ethics Committee and the authorities' data safety committees. The National Board of Health and Welfare is a government agency under the Ministry of Health and Social Affairs. It is not their policy to provide individual level data to researchers abroad. Instead, they normally advise researchers in other countries to cooperate with Swedish colleagues, to whom they can provide data according to standard legal provisions and procedures. Requests for access to the data can be made to the National Board of Health and Welfare and Statistics Sweden (http://www.socialstyrelsen.se/statistics; https://www.scb.se/en/services/guidance-for-researchers-and-universities/).

Contributorship statement

Sofia Zettermark: Conceptualization, design, analysis, interpretation of data, writing original draft, final approvement of version to be published.

Kani Kahlaf: Interpretation of data, revising draft critically for intellectual content, final approvement of version to be published.

Raquel Perez-Vicente: Design, analysis, interpretation of data, revising draft critically for intellectual content, final approvement of version to be published.

George Leckie: Analysis, interpretation of data, revising draft critically for intellectual content, final approvement of version to be published.

Diana Mulinari: Interpretation of data, revising draft critically for intellectual content, final approvement of version to be published.

Juan Merlo: Conceptualization, design, analysis, interpretation of data, revising draft critically for intellectual content, final approvement of version to be published.

REFERENCES

1 Bitzer J. Hormonal contraception and depression: another Pill scandal? *Eur J Contracept Repr* 2017;22(1):1-2.

For peer review only - http://bmjopen.bmj.com/site/about/guidelines.xhtml

2 Frokjaer VG. Pharmacological sex hormone manipulation as a risk model for depression. *J Neurosci Res* 2020;98(7):1283-92.
3 Rosenberg MJ, Waugh MS. Oral contraceptive discontinuation: a prospective evaluation of frequency and reasons. *Am J Obstet Gynecol* 1998;179(3;1):577-82.
4 Simmons RG, Sanders JN, Geist C, et al. Predictors of contraceptive switching and discontinuation within the first 6 months of use among Highly Effective Reversible Contraceptive Initiative Salt Lake study participants. *Am J Obstet Gynecol* 2019;220(4):376e1-e12.
5 Sanders SA, Graham CA, Bass JL, et al. A prospective study of the effects of oral contraceptives on sexuality and well-being and their relationship to discontinuation. *Contraception* 2001;64(1):51-8.
6 Skovlund CW, Morch LS, Kessing LV, et al. Association of Hormonal Contraception With Depression. *JAMA Psychiat* 2016;73(11):1154-62.
7 Zettermark S, Perez Vicente R, Merlo J. Hormonal contraception increases the risk of psychotropic drug use in adolescent girls but not in adults: A pharmacoepidemiological study on 800 000 Swedish women. *PLoS One* 2018;13(3):e0194773 doi:10.1371/journal.pone.0194773.
8 Zethraeus N, Dreber A, Ranehill E, et al. A first-choice combined oral contraceptive influences general well-being in healthy women: a double-blind, randomized, placebo-controlled trial. *Fertil Steril* 2017;107(5):1238-45.
9 Lundin C, Malmborg A, Slezak J, et al. Sexual function and combined oral contraceptives - a randomised, placebo-controlled trial. *Endocr Connect* 2018;7(11):1208-1216 doi:10.1530/EC-18-0384
10 Gingnell M, Engman J, Frick A, et al. Oral contraceptive use changes brain activity and mood in women with previous negative affect on the pill--a double-blinded, placebo-controlled randomized trial of a levonorgestrel-containing combined oral contraceptive. *Psychoneuroendocrino* 2013;38(7):1133-44.
11 Fisher PM, Larsen CB, Beliveau V et al. Pharmacologically Induced Sex Hormone Fluctuation Effects on Resting-State Functional Connectivity in a Risk Model for Depression: A Randomized Trial. *Neuropsychopharmacol* 2017;42(2):446-53.
12 Toffoletto S, Lanzenberger R, Gingnell M, et al. Emotional and cognitive functional imaging of estrogen and progesterone effects in the female human brain: A systematic review. *Psychoneuroendocrino* 2014;50:28-52.
13 Schaffir J, Worly BL, Gur TL. Combined hormonal contraception and its effects on mood: a critical review. *Eur J Contracept Repr* 2016;21(5):347-55.
14 Robakis T, Williams KE, Nutkiewicz L, et al. Hormonal Contraceptives and Mood: Review of the Literature and Implications for Future Research. *Curr Psychiat Rep* 2019;21(7):57. doi:10.1007/s11920-019-1034-z
15 Lewis CA, Kimmig AS, Zsido RG, et al. Effects of Hormonal Contraceptives on Mood: A Focus on Emotion Recognition and Reactivity, Reward Processing, and Stress Response. *Curr Psychiat Rep* 2019;21(11):115.
16 Worly BL, Gur TL, Schaffir J. The relationship between progestin hormonal contraception and depression: a systematic review. *Contraception* 2018;97(6):478-89.
17 de Wit AE, Booij SH, Giltay EJ, et al. Association of Use of Oral Contraceptives With Depressive Symptoms Among Adolescents and Young Women. *JAMA Psychiat* 2020;77(1):52-59 doi:10.1001/jamapsychiatry.2019.2838
18 Gregory ST, Hall K, Quast T, et al. Hormonal Contraception, depression, and Academic Performance among females attending college in the United States. *Psychiat Res* 2018;270:111-6.

For peer review only

For peer review only - http://bmjopen.bmj.com/site/about/guidelines.xhtml

19 Lundin C, Danielsson KG, Bixo M, et al. Combined oral contraceptive use is associated with both improvement and worsening of mood in the different phases of the treatment cycle - A double-blind, placebo-controlled randomized trial. *Psychoneuroendocrino* 2017;76:135-43.
20 Bengtsdotter H, Lundin C, Gemzell Danielsson K, Bixo M, Baumgart J, Marions L, et al. Ongoing or previous mental disorders predispose to adverse mood reporting during combined oral contraceptive use. *Eur J Contracept Repr* 2018;23(1):45-51.
21 Wemrell M, Mulinari S, Merlo J. An intersectional approach to multilevel analysis of individual heterogeneity (MAIH) and discriminatory accuracy. *Soc Sci Med* 2017;178:217-9.
22 Merlo J. Multilevel analysis of individual heterogeneity and discriminatory accuracy (MAIHDA) within an intersectional framework. *Soc Sci Med* 2018;203:74-80.
23 Evans CR, Williams DR, Onnela JP, et al. A multilevel approach to modeling health inequalities at the intersection of multiple social identities. *Soc Sci Med* 2018;203:64-73.
24 Bauer GR. Incorporating intersectionality theory into population health research methodology: Challenges and the potential to advance health equity. *Soc Sci Med* 2014;110:10–7.
25 McCall L. The complexity of intersectionlity. *Signs* 2005;30:1771-800.
26 Evans CR, Erickson N. Intersectionality and depression in adolescence and early adulthood: A MAIHDA analysis of the national longitudinal study of adolescent to adult health, 1995-2008. *Soc Sci Med* 2019;220:1-11.
27 Crenshaw K. Mapping the Margins: Intersectionality, Identity Politics, and Violence against Women of Color. *Stanford Law Rev* 1991;43(6):1241-99.
28 Cho S, Crenshaw K, McCall L. Toward a field of Interscetionality Studies: Theory, Applications and Praxis. *Signs* 2013;38(4):785-810.
29 Price K. It's Not Just About Abortion: Incorporating Intersectionality in Research About Women of Color and Reproduction. *Women Health Iss* 2011;21(3):55-7.
30 Ross LJ, Rickie S. Reproductive Justice: An Introduction. Oakland, CA: University of California Press 2017:1-360.
31 Dehlendorf C, Ruskin R, Grumbach K, et al. Recommendations for intrauterine contraception: a randomized trial of the effects of patients' race/ethnicity and socioeconomic status. *Am J Obstet Gynecol* 2010;203(4):319e1-8.
32 Montoya ER, Bos PA. How Oral Contraceptives Impact Social-Emotional Behavior and Brain Function. *Trends Cogn Sci* 2017;21(2):125-36.
33 Persmark A, Wemrell M, Zettermark S, et al. Precision public health: Mapping socioeconomic disparities in opioid dispensations at Swedish pharmacies by Multilevel Analysis of Individual Heterogeneity and Discriminatory Accuracy (MAIHDA). *PLoS One* 2019;14(8):e0220322 doi:10.1371/journal.pone.0220322
34 Hernandez-Yumar A, Wemrell M, Abasolo Alesson I, et al. Socioeconomic differences in body mass index in Spain: An intersectional multilevel analysis of individual heterogeneity and discriminatory accuracy. *PLoS One* 2018;13(12):e0208624.
35 Lorant V, Deliège D, Eaton W, et al. Socioeconomic inequalities in depression: a meta-analysis. *Am J Epidemiol* 2003;157(2):98-112.
36 Dehlendorf C, Rodriguez MI, Levy K, et al. Disparities in family planning. *Am J Obstet Gynecol* 2010;202(3):214-20.
37 Swedish National Board of Health and Welfare. DRG-koder och text 2019 [DRG codes and text 2019]. Available from: https://www.socialstyrelsen.se/utveckla-verksamhet/e-halsa/klassificering-och-koder/drg/drg-koder-och-definitioner/. [Accessed 2020 Dec 15]
38 Hankivsky O. Women's health, men's health, and gender and health: Implications of intersectionality. *Soc Sci Med* 2012;74(11):1712- 20.
39 Frederick A, Shrifrer D. Race and Disability: From Analogy to Intersectionality. *Sociology of Race and Ethnicity* 2019;5(2):200-14.

For peer review only - http://bmjopen.bmj.com/site/about/guidelines.xhtml

40 Knaak S, Mantler E, Szeto A. Mental illness-related stigma in healthcare: Barriers to access and care and evidence-based solutions. *Healthcare Management Forum* 2017;30(2):111-6.

41 Merlo J, Wagner P, Ghith N, et al. An Original Stepwise Multilevel Logistic Regression Analysis of Discriminatory Accuracy: The Case of Neighbourhoods and Health. *PLoS One* 2016;11(4):e0153778.

42 Axelsson Fisk S, Mulinari S, Wemrell M, et al. Chronic Obstructive Pulmonary Disease in Sweden: An intersectional multilevel analysis of individual heterogeneity and discriminatory accuracy. *SSM Popuulationl Health* 2018;4:334-46.

43 Leckie G, Charlton C. Runmlwin - a program to Run the MLwiN multilevel modelling software from within stata. *J Stat Softw* 2013;52(11):1-40.

44 Evans CR, Leckie G, Merlo J. Multilevel versus single-level regression for the analysis of multilevel information: The case of quantitative intersectional analysis. *Soc Sci Med* 2020;245:112499 doi:10.1016/j.socscimed.2019.112499.

45 Fruzzetti F, Fidecicchi T. Hormonal Contraception and Depression: Updated Evidence and Implications in Clinical Practice. *Clin Drug Investig*. 2020;40(12):1097-1106 doi: 10.1007/s40261-020-00966-8.

46 Segebladh B, Borgström A, Odlind V, et al. Prevalence of psychiatric disorders and premenstrual dysphoric symptoms in patients with experience of adverse mood during treatment with combined oral contraceptives. *Contraception* 2009;79(1):50-5.

47 Borgström A, Odlind V, Ekselius L, et al. Adverse mood effects of combined oral contraceptives in relation to personality traits. *Eur J Obstet Gyn R B* 2008;141(2):127-30.

48 Arrighi HM, Hertz-Picciotto I. The evolving concept of the healthy worker survivor effect *Epidemiology* 1995;5(2):189-96 doi: 10.1097/00001648-199403000-00009.

49 Josefsson A, Wiréhn A-B, Lindberg M, et al. Continuation rates of oral hormonal contraceptives in a cohort of first-time users: a population-based registry study, Sweden 2005–2010. *BMJ Open* 2013;3:e003401 doi:10.1136/bmjopen-2013-003401

50 Carlsten A, Waern M, Ekedahl A, et al. Antidepressant medication and suicide in Sweden. *Pharmacoepidem Dr S* 2001;10(6):525-30.

51 Kalmar S, Szanto K, Rihmer Z, et al. Antidepressant prescription and suicide rates: effect of age and gender. *Suicide Life Threat* 2008;38(4):363-74.

52 Mangrio E, Carlson E, Zdravkovic S. Understanding experiences of the Swedish health care system from the perspective of newly arrived refugees. *BMC Research Notes* 2018;11(1):616.

53 Kirmayer LJ, Narasiah L, Munoz M, et al. Common mental health problems in immigrants and refugees: general approach in primary care. *Can Med Assoc J* 2011;183(12):E959-67.

54 Ditch S, Roberts TA, Hansen S. The influence of health care utilization on the association between hormonal contraception initiation and subsequent depression diagnosis and antidepressant use. *Contraception* 2020;101(4):237-43.

55 Brenner H. Political economy and health. In: Amick III B, Levine S, Tarlov A, et al, eds. Society and health. New York, NY: Oxford university press 1995:211–46.

56 Lynch JW, Kaplan GA, Salonen JT. Why do poor people behave poorly? Variation in adult health behaviours and psychosocial characteristics by stages of the socioeconomic lifecourse. *Soc Sci Med* 1997;44(6):809-19.

57 Collins PY, von Unger H, Armbrister A. Church ladies, good girls, and locas: stigma and the intersection of gender, ethnicity, mental illness, and sexuality in relation to HIV risk. *Soc Sci Med* 2008;67(3):389-97.

58 Honjo K. Social epidemiology: Definition, history, and research examples. *Environ Health Prev* 2004;9(5):193-9.

For peer review only - http://bmjopen.bmj.com/site/about/guidelines.xhtml

59 Kubzansky L, Kawachi I. Affective States and Health. In: Berkman L, Kawachi I, eds. Social epidemiology. New York, NY: Oxford University Press 2000:13–41.
60 Krieger N. Epidemiology and the Peoples Health - Theory and Context. Oxford: Oxford University Press 2011:1-381.
61 Krieger N. Epidemiology and social sciences: towards a critical reengagement in the 21st century. *Epidemiol Rev* 2000;22(1):155-63.
62 Link B, Phelan J. Social conditions as fundamental causes of disease. *J Health Soc Behav* 1995(Extra Issue:Forty Years of Medical Sociology):80-94
59 Schatzberg AF. New indications for antidepressants. *J Clin Psychiat* 2000;61(Suppl)11:9-17.
63 Gardarsdottir H, Heerdink ER, van Dijk L, et al. Indications for antidepressant drug prescribing in general practice in the Netherlands. *J Affect Disorders* 2007;98(1-2):109-15.
64 Merlo J, Mulinari S, Wemrell M, et al. The tyranny of the averages and the indiscriminate use of risk factors in public health: The case of coronary heart disease. *SSM Population Health* 2017;3:684-98 doi:10.1016/j.ssmph.2017.08.005
65 Warner LR. A Best Practices Guide to Intersectional Approaches in Psychological Research. *Sex Roles* 2008;59:454–63.
66 Marmot M. Social justice, epidemiology and health inequalities. *Eur J Epidemiol* 2017;32(7):537-46.

For peer review only

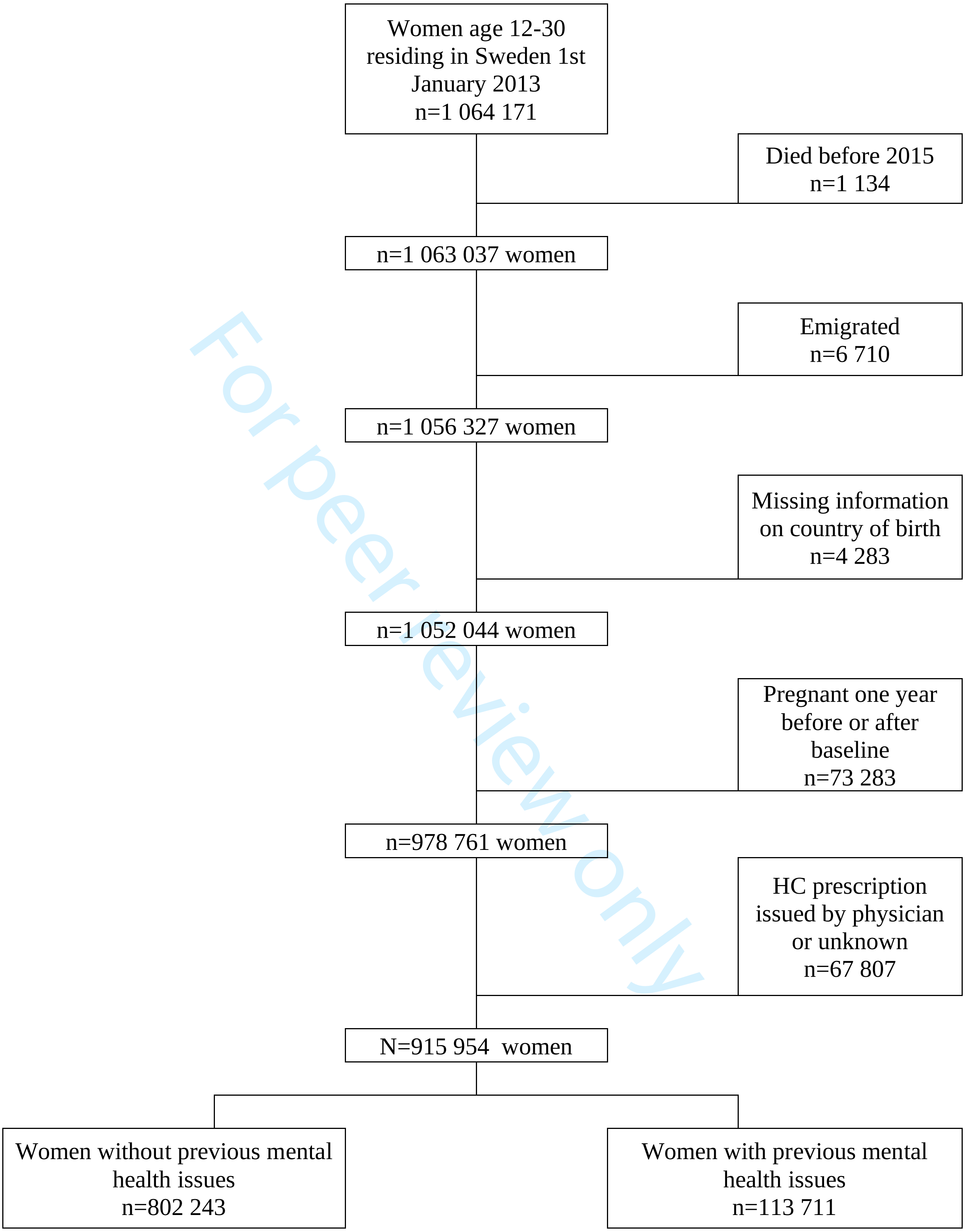

For peer review only

Figure 1. Selection of the study population.

For peer review only - http://bmjopen.bmj.com/site/about/guidelines.xhtml

```
* Hormonal Contraception and Antidepressant Use in Sweden: An
Intersectional Multilevel Analysis of Individual Heterogeneity and
Discriminatory Accuracy
* (MAIHDA)
***************************************************************************
clear *
global MLwiN_path "C:\Program Files\MLwiN v3.05\mlwin.exe"
set cformat %9.2f



***************************************************************************
* TABLE 1
***************************************************************************

* Load the data
use "final_mlMENTAL.dta", clear
keep age_cat1 age_cat2 age_cat3 inc1 inc2 inc3 imm pp proportion denom
order age_cat1 age_cat2 age_cat3 inc1 inc2 inc3 imm pp proportion denom

generate percentage = 100*proportion
drop proportion
format %9.2f percentage

generate age_cat = .
replace age_cat = 1 if age_cat1==1
replace age_cat = 2 if age_cat2==1
replace age_cat = 3 if age_cat3==1

generate inc_cat = .
replace inc_cat = 1 if inc1==1
replace inc_cat = 2 if inc2==1
replace inc_cat = 3 if inc3==1

* Results for the table
tabulate pp [fweight = denom]
table pp [fweight = denom], contents(mean percentage )
tabulate age_cat pp [fweight = denom], column nofreq
tabulate inc_cat pp [fweight = denom], column nofreq
tabulate imm pp [fweight = denom], column nofreq



***************************************************************************
* TABLE 2: MODEL 1
***************************************************************************

* Load the data
use "final_mlMENTAL.dta", clear

* IGLS estimation, for MCMC initial values
runmlwin prop cons, ///
  level2(inter: cons) ///
  level1(inter:) ///
  discrete(distribution(binomial) link(logit) denom(denom) mql1) ///
  nopause

* MCMC
runmlwin prop cons, ///
  level2(inter: cons, residuals(u, savechains("m1u.dta",replace))) ///
  level1(inter:) ///
```

```
  discrete(distribution(binomial) link(logit) denom(denom)) ///
  mcmc(burnin(10000) chain(50000) thin(10) savechains("m1b.dta", replace))
///
  initsprevious ///
  nopause

* Level-2 variance
scalar m1sigma2u = [RP2]var(cons)
scalar list m1sigma2u

* Level-1 variance
scalar m1sigma2e = _pi^2/3
scalar list m1sigma2e

* VPC
display "VPC_u = " %9.4f m1sigma2u/(m1sigma2u + m1sigma2e)

* Compress and save the data
compress
save "m1.dta", replace



*---------------------------------------------------------------------------*
* PREPARE FIXED-PART PAREMETER CHAINS
*---------------------------------------------------------------------------*

use "m1b.dta", clear
drop deviance RP2_var_cons_ OD_bcons_1
rename FP1_* b_*
format %9.2f b_*
compress
save "m1b_prepped.dta", replace
isid iteration
codebook iteration, compact



*---------------------------------------------------------------------------*
* PREPARE RANDOM EFFECTS CHAINS
*---------------------------------------------------------------------------*
use "m1u.dta", clear
drop residual idnum
rename value u
format %9.2f u
sort inter iteration
order inter iteration
compress
save "m1u_prepped.dta", replace
isid inter iteration
codebook iteration, compact



*---------------------------------------------------------------------------*
* MERGE DATA, FIXED-PART PARAMETER AND RANDOM EFFECT CHAINS TOGETHER
*---------------------------------------------------------------------------*
use "final_mlMENTAL", clear
count
cross using "m1b_prepped.dta"
count
```

```
merge m:1 inter iteration using "m1u_prepped.dta", nogenerate assert(match)
count
compress
save "m1data_prepped.dta", replace



*------------------------------------------------------------------------*
* ROC
*------------------------------------------------------------------------*
use "m1data_prepped.dta", clear
count
generate p = invlogit(b_cons + u)
gcollapse (mean) p, by(inter num denom)
count
expand denom
sort inter
bysort inter: generate y = (_n<=numerator)
generate prop = denom/_N
generate weight = int(1/prop)
roctab y p [fw=weight]



*------------------------------------------------------------------------*
* TABLE 3
*------------------------------------------------------------------------*
use "m1data_prepped.dta", clear
keep iteration inter age_cat1 age_cat2 age_cat3 inc1 inc2 inc3 imm pp denom
b_cons u
count
generate p = 100*invlogit(b_cons + u)
drop b_cons u
format %9.1f p
drop inter
reshape wide denom p, i(iteration age_cat1 age_cat2 age_cat3 inc1 inc2 inc3
imm) j(pp)
generate denom = denom0 + denom1
drop denom0 denom1
generate pdiff = p1 - p0
gcollapse (mean) p0 p1 pdiff (p2.5) pdifflo=pdiff (p97.5) pdiffhi=pdiff,
by(age_cat1 age_cat2 age_cat3 inc1 inc2 inc3 imm denom)
format %9.1f pdiff pdifflo pdiffhi
order p1 p0 pdiff pdifflo pdiffhi, last
gsort -age_cat1 -age_cat2 -age_cat3 -inc1 -inc2 -inc3 imm



**************************************************************************
* TABLE 2: MODEL 2:
**************************************************************************

* Load the data
use "final_mlMENTAL.dta", clear

* IGLS estimation, for MCMC initial values
runmlwin prop cons age_cat2 age_cat3 inc1 inc2 imm pp, ///
  level2(inter: cons) ///
  level1(inter:) ///
  discrete(distribution(binomial) link(logit) denom(denom) mql1) ///
  nopause
```

```
* MCMC
runmlwin prop cons age_cat2 age_cat3 inc1 inc2 imm  pp, ///
  level2(inter: cons, residuals(u,savechains("m2u.dta",replace))) ///
  level1(inter:) ///
  discrete(distribution(binomial) link(logit) denom(denom)) ///
  mcmc(burnin(10000) chain(50000) thin(10) savechains("m2b.dta", replace))
///
  initsprevious ///
  nopause

* Odds ratios
runmlwin, or

* Level-2 variance
scalar m2sigma2u = [RP2]var(cons)
scalar list m2sigma2u

* Level-1 variance
scalar m2sigma2e = _pi^2/3
scalar list m2sigma2e

* VPC
display "VPC_u = " %9.4f m2sigma2u/(m2sigma2u + m2sigma2e)

* Compress and save the data
compress
save "m2.dta", replace

* PCV
display "PCV = " %9.4f (m2sigma2u - m1sigma2u)/m1sigma2u



*----------------------------------------------------------------------------*
* PREPARE FIXED-PART PAREMETER CHAINS
*----------------------------------------------------------------------------*
use "m2b.dta", clear
drop deviance RP2_var_cons_ OD_bcons_1
rename FP1_* b_*
format %9.2f b_*
compress
save "m2b_prepped.dta", replace
isid iteration
codebook iteration, compact



*----------------------------------------------------------------------------*
* PREPARE inter RANDOM EFFECTS CHAINS
*----------------------------------------------------------------------------*
use "m2u.dta", clear
drop residual idnum
rename value u
format %9.2f u
sort inter iteration
order inter iteration
compress
save "m2u_prepped.dta", replace
isid inter iteration
codebook iteration, compact
```

```
*--------------------------------------------------------------------------*
* MERGE DATA, FIXED-PART PARAMETER AND RANDOM EFFECT CHAINS TOGETHER
*--------------------------------------------------------------------------*
use "final_mlMENTAL", clear
count
cross using "m2b_prepped.dta"
count
merge m:1 inter iteration using "m2u_prepped.dta"
count
save "m2data_prepped.dta", replace



*--------------------------------------------------------------------------*
* ROC
*--------------------------------------------------------------------------*
use "m2data_prepped.dta", clear
count
generate p = invlogit(b_cons + b_age_cat2*age_cat2 + b_age_cat3*age_cat3 +
b_inc1*inc1 + b_inc2*inc2 + b_imm*imm + b_pp*pp)
gcollapse (mean) p, by(inter num denom)
count
expand denom
sort inter
bysort inter: generate y = (_n<=numerator)
generate prop = denom/_N
generate weight = int(1/prop)
roctab y p [fw=weight]



*--------------------------------------------------------------------------*
* TABLE 3
*--------------------------------------------------------------------------*
use "m1data_prepped.dta", clear
keep iteration inter age_cat1 age_cat2 age_cat3 inc1 inc2 inc3 imm pp denom
b_cons u
count
generate p = 100*invlogit(b_cons + u)
drop b_cons u
format %9.1f p
drop inter
reshape wide denom p, i(iteration age_cat1 age_cat2 age_cat3 inc1 inc2 inc3
imm) j(pp)
generate denom = denom0 + denom1
drop denom0 denom1
generate pdiff = p1 - p0
gcollapse (mean) p0 p1 pdiff (p2.5) pdifflo=pdiff (p97.5) pdiffhi=pdiff,
by(age_cat1 age_cat2 age_cat3 inc1 inc2 inc3 imm denom)
format %9.1f pdiff pdifflo pdiffhi
order p1 p0 pdiff pdifflo pdiffhi, last
gsort -age_cat1 -age_cat2 -age_cat3 -inc1 -inc2 -inc3 imm



****************************************************************************
exit
```

```
******************************************************************************
* Hormonal Contraception and Antidepressant Use in Sweden: An
Intersectional Multilevel Analysis of Individual Heterogeneity and
Discriminatory Accuracy (MAIHDA)

******************************************************************************
clear *
global MLwiN_path "C:\Program Files\MLwiN v3.05\mlwin.exe"
set cformat %9.2f



******************************************************************************
* TABLE 1
******************************************************************************

* Load the data
use "final_mlNoMENTAL.dta", clear
keep age_cat1 age_cat2 age_cat3 inc1 inc2 inc3 imm pp proportion denom
order age_cat1 age_cat2 age_cat3 inc1 inc2 inc3 imm pp proportion denom

generate percentage = 100*proportion
drop proportion
format %9.2f percentage

generate age_cat = .
replace age_cat = 1 if age_cat1==1
replace age_cat = 2 if age_cat2==1
replace age_cat = 3 if age_cat3==1

generate inc_cat = .
replace inc_cat = 1 if inc1==1
replace inc_cat = 2 if inc2==1
replace inc_cat = 3 if inc3==1

* Results for the table
tabulate pp [fweight = denom]
table pp [fweight = denom], contents(mean percentage )
tabulate age_cat pp [fweight = denom], column nofreq
tabulate inc_cat pp [fweight = denom], column nofreq
tabulate imm pp [fweight = denom], column nofreq



******************************************************************************
* TABLE 2: MODEL 1
******************************************************************************

* Load the data
use "final_mlNoMENTAL.dta", clear

* IGLS estimation, for MCMC initial values
runmlwin prop cons, ///
  level2(inter: cons) ///
  level1(inter:) ///
  discrete(distribution(binomial) link(logit) denom(denom) mql1) ///
  nopause

* MCMC
runmlwin prop cons, ///
  level2(inter: cons, residuals(u, savechains("m1u.dta",replace))) ///
```

```
  level1(inter:) ///
  discrete(distribution(binomial) link(logit) denom(denom)) ///
  mcmc(burnin(10000) chain(50000) thin(10) savechains("m1b.dta", replace))
///
  initsprevious ///
  nopause

* Level-2 variance
scalar m1sigma2u = [RP2]var(cons)
scalar list m1sigma2u

* Level-1 variance
scalar m1sigma2e = _pi^2/3
scalar list m1sigma2e

* VPC
display "VPC_u = " %9.4f m1sigma2u/(m1sigma2u + m1sigma2e)

* Compress and save the data
compress
save "m1.dta", replace



*---------------------------------------------------------------------------*
* PREPARE FIXED-PART PAREMETER CHAINS
*---------------------------------------------------------------------------*

use "m1b.dta", clear
drop deviance RP2_var_cons_ OD_bcons_1
rename FP1_* b_*
format %9.2f b_*
compress
save "m1b_prepped.dta", replace
isid iteration
codebook iteration, compact



*---------------------------------------------------------------------------*
* PREPARE RANDOM EFFECTS CHAINS
*---------------------------------------------------------------------------*
use "m1u.dta", clear
drop residual idnum
rename value u
format %9.2f u
sort inter iteration
order inter iteration
compress
save "m1u_prepped.dta", replace
isid inter iteration
codebook iteration, compact



*---------------------------------------------------------------------------*
* MERGE DATA, FIXED-PART PARAMETER AND RANDOM EFFECT CHAINS TOGETHER
*---------------------------------------------------------------------------*
use "final_mlNoMENTAL", clear
count
cross using "m1b_prepped.dta"
```

```
count
merge m:1 inter iteration using "m1u_prepped.dta", nogenerate assert(match)
count
compress
save "m1data_prepped.dta", replace


*---------------------------------------------------------------------------*
* ROC
*---------------------------------------------------------------------------*
use "m1data_prepped.dta", clear
count
generate p = invlogit(b_cons + u)
gcollapse (mean) p, by(inter num denom)
count
expand denom
sort inter
bysort inter: generate y = (_n<=numerator)
generate prop = denom/_N
generate weight = int(1/prop)
roctab y p [fw=weight]


*---------------------------------------------------------------------------*
* TABLE 3
*---------------------------------------------------------------------------*
use "m1data_prepped.dta", clear
keep iteration inter age_cat1 age_cat2 age_cat3 inc1 inc2 inc3 imm pp denom
b_cons u
count
generate p = 100*invlogit(b_cons + u)
drop b_cons u
format %9.1f p
drop inter
reshape wide denom p, i(iteration age_cat1 age_cat2 age_cat3 inc1 inc2 inc3
imm) j(pp)
generate denom = denom0 + denom1
drop denom0 denom1
generate pdiff = p1 - p0
gcollapse (mean) p0 p1 pdiff (p2.5) pdifflo=pdiff (p97.5) pdiffhi=pdiff,
by(age_cat1 age_cat2 age_cat3 inc1 inc2 inc3 imm denom)
format %9.1f pdiff pdifflo pdiffhi
order p1 p0 pdiff pdifflo pdiffhi, last
gsort -age_cat1 -age_cat2 -age_cat3 -inc1 -inc2 -inc3 imm


****************************************************************************
* TABLE 2: MODEL 2:
****************************************************************************

* Load the data
use "final_mlNoMENTAL.dta", clear

* IGLS estimation, for MCMC initial values
runmlwin prop cons age_cat2 age_cat3 inc1 inc2 imm pp, ///
  level2(inter: cons) ///
  level1(inter:) ///
  discrete(distribution(binomial) link(logit) denom(denom) mql1) ///
```

```
  nopause

* MCMC
runmlwin prop cons age_cat2 age_cat3 inc1 inc2 imm  pp, ///
  level2(inter: cons, residuals(u,savechains("m2u.dta",replace))) ///
  level1(inter:) ///
  discrete(distribution(binomial) link(logit) denom(denom)) ///
  mcmc(burnin(10000) chain(50000) thin(10) savechains("m2b.dta", replace))
///
  initsprevious ///
  nopause

* Odds ratios
runmlwin, or

* Level-2 variance
scalar m2sigma2u = [RP2]var(cons)
scalar list m2sigma2u

* Level-1 variance
scalar m2sigma2e = _pi^2/3
scalar list m2sigma2e

* VPC
display "VPC_u = " %9.4f m2sigma2u/(m2sigma2u + m2sigma2e)

* Compress and save the data
compress
save "m2.dta", replace

* PCV
display "PCV = " %9.4f (m2sigma2u - m1sigma2u)/m1sigma2u



*----------------------------------------------------------------------------*
* PREPARE FIXED-PART PAREMETER CHAINS
*----------------------------------------------------------------------------*
use "m2b.dta", clear
drop deviance RP2_var_cons_ OD_bcons_1
rename FP1_* b_*
format %9.2f b_*
compress
save "m2b_prepped.dta", replace
isid iteration
codebook iteration, compact



*----------------------------------------------------------------------------*
* PREPARE inter RANDOM EFFECTS CHAINS
*----------------------------------------------------------------------------*
use "m2u.dta", clear
drop residual idnum
rename value u
format %9.2f u
sort inter iteration
order inter iteration
compress
save "m2u_prepped.dta", replace
isid inter iteration
```

```
codebook iteration, compact



*------------------------------------------------------------------------------*
* MERGE DATA, FIXED-PART PARAMETER AND RANDOM EFFECT CHAINS TOGETHER
*------------------------------------------------------------------------------*
use "final_mlNoMENTAL", clear
count
cross using "m2b_prepped.dta"
count
merge m:1 inter iteration using "m2u_prepped.dta"
count
save "m2data_prepped.dta", replace



*------------------------------------------------------------------------------*
* ROC
*------------------------------------------------------------------------------*
use "m2data_prepped.dta", clear
count
generate p = invlogit(b_cons + b_age_cat2*age_cat2 + b_age_cat3*age_cat3 +
b_inc1*inc1 + b_inc2*inc2 + b_imm*imm + b_pp*pp)
gcollapse (mean) p, by(inter num denom)
count
expand denom
sort inter
bysort inter: generate y = (_n<=numerator)
generate prop = denom/_N
generate weight = int(1/prop)
roctab y p [fw=weight]



*------------------------------------------------------------------------------*
* TABLE 3
*------------------------------------------------------------------------------*
use "m1data_prepped.dta", clear
keep iteration inter age_cat1 age_cat2 age_cat3 inc1 inc2 inc3 imm pp denom
b_cons u
count
generate p = 100*invlogit(b_cons + u)
drop b_cons u
format %9.1f p
drop inter
reshape wide denom p, i(iteration age_cat1 age_cat2 age_cat3 inc1 inc2 inc3
imm) j(pp)
generate denom = denom0 + denom1
drop denom0 denom1
generate pdiff = p1 - p0
gcollapse (mean) p0 p1 pdiff (p2.5) pdifflo=pdiff (p97.5) pdiffhi=pdiff,
by(age_cat1 age_cat2 age_cat3 inc1 inc2 inc3 imm denom)
format %9.1f pdiff pdifflo pdiffhi
order p1 p0 pdiff pdifflo pdiffhi, last
gsort -age_cat1 -age_cat2 -age_cat3 -inc1 -inc2 -inc3 imm



********************************************************************************
exit
```

For peer review only

```
pp,imm,inter,age_cat1,age_cat2,age_cat3,inc1,inc2,inc3,proportion,numerator,denom,cons
0,0,12-17 Low income 0 0,1,0,0,1,0,0,.013224002,279,21098,1
1,0,12-17 Low income 0 1,1,0,0,1,0,0,.037408244,265,7084,1
0,1,12-17 Low income 1 0,1,0,0,1,0,0,.0040497542,28,6914,1
1,1,12-17 Low income 1 1,1,0,0,1,0,0,.0096021947,7,729,1
0,0,12-17 Middle income 0 0,1,0,0,0,1,0,.010099272,587,58123,1
1,0,12-17 Middle income 0 1,1,0,0,0,1,0,.030316716,537,17713,1
0,1,12-17 Middle income 1 0,1,0,0,0,1,0,.0056107035,52,9268,1
1,1,12-17 Middle income 1 1,1,0,0,0,1,0,.017814728,15,842,1
0,0,12-17 High income 0 0,1,0,0,0,0,1,.008893352,859,96589,1
1,0,12-17 High income 0 1,1,0,0,0,0,1,.01951286,572,29314,1
0,1,12-17 High income 1 0,1,0,0,0,0,1,.0076045627,30,3945,1
1,1,12-17 High income 1 1,1,0,0,0,0,1,.025718609,17,661,1
0,0,18-23 Low income 0 0,0,1,0,1,0,0,.029676914,530,17859,1
1,0,18-23 Low income 0 1,0,1,0,1,0,0,.034916617,938,26864,1
0,1,18-23 Low income 1 0,0,1,0,1,0,0,.011607248,98,8443,1
1,1,18-23 Low income 1 1,0,1,0,1,0,0,.022702307,62,2731,1
0,0,18-23 Middle income 0 0,0,1,0,0,1,0,.027664155,771,27870,1
1,0,18-23 Middle income 0 1,0,1,0,0,1,0,.0282459,1247,44148,1
0,1,18-23 Middle income 1 0,0,1,0,0,1,0,.011609907,75,6460,1
1,1,18-23 Middle income 1 1,0,1,0,0,1,0,.023316063,54,2316,1
0,0,18-23 High income 0 0,0,1,0,0,0,1,.023347162,1058,45316,1
1,0,18-23 High income 0 1,0,1,0,0,0,1,.022887168,2082,90968,1
0,1,18-23 High income 1 0,0,1,0,0,0,1,.017995911,44,2445,1
1,1,18-23 High income 1 1,0,1,0,0,0,1,.019577537,38,1941,1
0,0,24-30 Low income 0 0,0,0,1,1,0,0,.032189574,2168,67351,1
1,0,24-30 Low income 0 1,0,0,1,1,0,0,.031126546,1954,62776,1
0,1,24-30 Low income 1 0,0,0,1,1,0,0,.013751426,446,32433,1
1,1,24-30 Low income 1 1,0,0,1,1,0,0,.026964672,187,6935,1
0,0,24-30 Middle income 0 0,0,0,1,0,1,0,.030455342,818,26859,1
1,0,24-30 Middle income 0 1,0,0,1,0,1,0,.03591495,652,18154,1
0,1,24-30 Middle income 1 0,0,0,1,0,1,0,.023714487,202,8518,1
1,1,24-30 Middle income 1 1,0,0,1,0,1,0,.027789129,68,2447,1
0,0,24-30 High income 0 0,0,0,1,0,0,1,.025993951,593,22813,1
1,0,24-30 High income 0 1,0,0,1,0,0,1,.024208747,501,20695,1
0,1,24-30 High income 1 0,0,0,1,0,0,1,.023088569,61,2642,1
1,1,24-30 High income 1 1,0,0,1,0,0,1,.019407559,19,979,1
```

For peer review only

```
pp,imm,inter,age_cat1,age_cat2,age_cat3,inc1,inc2,inc3,proportion,numerator,denom,cons
0,0,12-17 Low income 0 0,1,0,0,1,0,0,.22574355,463,2051,1
1,0,12-17 Low income 0 1,1,0,0,1,0,0,.3049593,412,1351,1
0,1,12-17 Low income 1 0,1,0,0,1,0,0,.12383901,40,323,1
1,1,12-17 Low income 1 1,1,0,0,1,0,0,.1891892,21,111,1
0,0,12-17 Middle income 0 0,1,0,0,0,1,0,.23362993,1024,4383,1
1,0,12-17 Middle income 0 1,1,0,0,0,1,0,.31201944,771,2471,1
0,1,12-17 Middle income 1 0,1,0,0,0,1,0,.13422818,60,447,1
1,1,12-17 Middle income 1 1,1,0,0,0,1,0,.18032786,22,122,1
0,0,12-17 High income 0 0,1,0,0,0,0,1,.28093326,1469,5229,1
1,0,12-17 High income 0 1,1,0,0,0,0,1,.34217408,916,2677,1
0,1,12-17 High income 1 0,1,0,0,0,0,1,.18867925,50,265,1
1,1,12-17 High income 1 1,1,0,0,0,0,1,.3018868,32,106,1
0,0,18-23 Low income 0 0,0,1,0,1,0,0,.37809917,2013,5324,1
1,0,18-23 Low income 0 1,0,1,0,1,0,0,.39212543,2201,5613,1
0,1,18-23 Low income 1 0,0,1,0,1,0,0,.19350649,149,770,1
1,1,18-23 Low income 1 1,0,1,0,1,0,0,.28291318,101,357,1
0,0,18-23 Middle income 0 0,0,1,0,0,1,0,.36302635,2164,5961,1
1,0,18-23 Middle income 0 1,0,1,0,0,1,0,.37776819,2627,6954,1
0,1,18-23 Middle income 1 0,0,1,0,0,1,0,.19285715,108,560,1
1,1,18-23 Middle income 1 1,0,1,0,0,1,0,.27112675,77,284,1
0,0,18-23 High income 0 0,0,1,0,0,0,1,.39782199,2959,7438,1
1,0,18-23 High income 0 1,0,1,0,0,0,1,.38269973,3765,9838,1
0,1,18-23 High income 1 0,0,1,0,0,0,1,.25,82,328,1
1,1,18-23 High income 1 1,0,1,0,0,0,1,.27906978,84,301,1
0,0,24-30 Low income 0 0,0,0,1,1,0,0,.49862742,9082,18214,1
1,0,24-30 Low income 0 1,0,0,1,1,0,0,.50085437,5569,11119,1
0,1,24-30 Low income 1 0,0,0,1,1,0,0,.32457545,1013,3121,1
1,1,24-30 Low income 1 1,0,0,1,1,0,0,.37422037,360,962,1
0,0,24-30 Middle income 0 0,0,0,1,0,1,0,.50859779,2869,5641,1
1,0,24-30 Middle income 0 1,0,0,1,0,1,0,.49799198,1488,2988,1
0,1,24-30 Middle income 1 0,0,0,1,0,1,0,.37214136,358,962,1
1,1,24-30 Middle income 1 1,0,0,1,0,1,0,.33590734,87,259,1
0,0,24-30 High income 0 0,0,0,1,0,0,1,.48993289,1971,4023,1
1,0,24-30 High income 0 1,0,0,1,0,0,1,.48666918,1296,2663,1
0,1,24-30 High income 1 0,0,0,1,0,0,1,.37669376,139,369,1
1,1,24-30 High income 1 1,0,0,1,0,0,1,.45238096,57,126,1
```

For peer review only

Supplementary material 5

Supplementary table, summary statistics. Numbers are percentages (numbers within brackets).

		Hormonal contraception	Mental health issues
Age	12-17	23.2 (63 181)	7.2 (19 536)
	18-23	59.9 (192 315)	13.6 (43 729)
	24-30	40.3 (130 103)	15.6 (50 447)
Income	Low	40.8 (126 632)	15.9 (49 316)
	Middle	38.9 (98 698)	12.2 (31 032)
	High	45.6 (160 269)	9.5 (33 363)
Immigrant background	No	45.1 (363 390)	12.9 (103 938)
	Yes	20.1 (22 209)	8.9 (9 773)

Supplementary table. Percentage of women within each intersectional dimension using hormonal contraceptives and with previous mental health issues.

For peer review only

Supplementary material 6

ATC	Freq.	Percent
G02BA03	12 535	3.25
G02BB	96	0.02
G0BB01	26 022	6.75
G02BB01	48	0.01
G03AA03	4 786	1.24
G03AA07	126 061	32.69
G03AA09	3 227	0.84
G03AA11	15 463	4.01
G03AA12	4 596	13.69
G03AA13	12 329	1.19
G03AA14	5 958	3.20
G03AB	5 958	1.55
G03AB03	8 014	2.08
G03AB04	5 341	1.39
G03AC01	4 249	1.10
G03AC02	2 483	0.64
G03AC06	2 710	0.70
G03AC08	21 284	5.52
G03AC09	77 595	20.12

Supplementary table, frequency table of hormonal contraceptives. Frequency of all included hormonal contraceptives in the final cohort of 915 954 women.

For peer review only

Supplementary material 7

Sensitivity analysis only including only new users and never-users of HC. Women with any dispensed prescription of HC during five years prior to baseline were excluded and only women with a HC prescription fill exclusively during follow-up are included as users. Non-users of HC are defined as not filing any prescription of HC during five years prior to baseline or during follow-up.

Table 1. Characteristics of the 532 543 women aged 12 - 30 years by previous mental health issues and use of hormonal contraceptives. Values are percentages (number of women in parenthesis).

	Previous mental health issues			
	Yes 11.12 (59 238)		No 88.88 (473 305)	
	Use of HC		Use of HC	
	Yes 38.87 (23 034)	No 61.13 (36 214)	Yes 1.83 (8 678)	No 98.17 (464 627)
Antidepressant during follow-up	60.11 (35 610)	39.89 (23 629)		
Age				
12-17 years	32.53 (4 065)	25.56 (11 946)	43.24 (38 426)	50.37 (193 658)
18-23 years	37.18 (4 646)	27.22 (12 722)	35.44 (31 489)	20.09 (77 244)
24-30 years	30.28 (3 784)	47.23 (22 075)	21.32 (18 948)	29.53 (113 540)
Income level				
Low inc.	35.93 (4 489)	43.77 (20 458)	24.00 (21 331)	30.34 (116 635)
Medium inc.	30.21 (3 775)	28.13 (13 147)	29.43 (26 151)	30.46 (117 109)
High inc.	33.86 (4 231)	28.11 (13 138)	46.57 (41 381)	39.20 (150 698)
Immigrant background				
None	92.84 (11 600)	88.12 (41 188)	91.78 (81 560)	81.40 (312 926)
Yes	7.16 (895)	11.88 (5 555)	8.22 (7 303)	18.60 (71 516)

For peer review only

Table 2. Results from the Multilevel Analysis of Individual Heterogeneity and Discriminatory Accuracy (MAIHDA) distinguishing between measures of association (Odds Ratios) and measures of variance and discriminatory accuracy. The analyses are stratified by the existence of previous mental issues. Values are point estimations (with 95% credible intervals) or percentages where indicated.

	Without metal health issues		With mental health issues	
	Model 1	Model 2	Model 1	Model 2
**Measures of association**				
Age				
12-17 years		Reference		Reference
18-23 years		1.76 (1.35-2.21)		1.64 (1.42-1.89)
24-30 years		2.34 (1.79-2.90)		2.69 (2.32-3.09)
Income				
High inc.		Reference		Reference
Medium inc.		1.06 (0.81-1.34)		0.84 (0.72-0.98)
Low inc.		1.08 (0.86-1.35)		0.87 (0.75-1.00)
Immigrant background				
None		Reference		Reference
Yes		0.63 (0.51-0.76)		0.52 (0.46-0.59)
Hormonal contraception				
No		Reference		Reference
Yes		1.86 (1.51-2.28)		1.18 (1.05-1.34)
**Measures of variance and discriminatory accuracy***				
Variance	0.36 (0.22-0.60)	0.08 (0.04-0.15)	0.31 (0.19-0.51)	0.02 (0.01-0.14)
VPC	9.88%	2.34%	8.67%	0.63%
PCV		76.32%		92.73%
AUC	0.63 (0.63-0.63)	0.62 (0.62-0.62)	0.65 (0.64-0.65)	0.64 (0.64-0.64)

*Between-strata variance, variance partition coefficient (VPC), proportional change of the variance (PCV), Area under the curve (AUC)

For peer review only

Table 3. Distribution of antidepressant use between different intersectional strata, and difference in usage between user and non-users of hormonal contraceptives but otherwise sharing the same intersectional stratum. The values are calculated from the multilevel analysis of individual heterogeneity and discriminatory accuracy (MAIHDA). Numbers are percentages.

Previous mental health issues	Age (years)	Income level	Immigrant background	Number of women	Use of hormonal contraceptives (%)		
					Yes	No	Yes-No difference
No	12 – 17	Low	No	25342	3.7	1.2	2.4 (1.9-3)
			Yes	7416	1.2	0.4	0.7 (0.1-1.6)
		Middle	No	69096	2.9	1	1.9 (1.6-2.2)
			Yes	9839	2	0.6	1.4 (0.5-2.5)
		High	No	115995	1.9	0.9	1 (0.8-1.2)
			Yes	4396	2.5	0.8	1.7 (0.6-3.2)
	18 – 23	Low	No	15523	3.9	2.7	1.2 (0.5-1.8)
			Yes	8238	2.2	1.1	1 (0.2-2)
		Middle	No	27757	2.9	2.2	0.7 (0.3-1.1)
			Yes	6642	2.2	1.1	1.1 (0.2-2.1)
		High	No	47988	2.3	2	0.3 (0-0.6)
			Yes	2585	2.5	1.8	0.7 (-0.5-2.1)
	24 – 30	Low	No	51819	4.2	3.3	0.9 (0.4-1.3)
			Yes	29628	2.7	1.3	1.4 (0.8-2.1)
		Middle	No	22251	4.7	2.8	1.9 (1.2-2.6)
			Yes	7675	2.8	2.1	0.6 (-0.4-1.8)
		High	No	18715	3.1	2.6	0.6 (0-1.2)
			Yes	2400	2.9	2.3	0.6 (-1-2.7)
Yes	12 – 17	Low	No	2671	30.5	21.8	8.6 (4.9-12.4)
			Yes	372	19.7	12.6	7.1 (-1.3-16.6)
		Middle	No	5554	31.6	22.9	8.7 (6-11.5)
			Yes	507	17.3	14.4	2.9 (-4.8-11.9)
		High	No	6585	35.2	27.9	7.3 (4.6-10)
			Yes	322	29.5	19.6	9.9 (-0.5-21.1)
	18 – 23	Low	No	4197	38.9	39	-0.1 (-3.5-3.4)
			Yes	666	29.2	20.1	9.1 (0.7-17.9)
		Middle	No	5049	38.2	36.4	1.8 (-1.2-4.8)
			Yes	549	31.1	18.6	12.5 (3.2-22.2)

For peer review only

	High	No	6601	39.5	40.6	-1.1 (-3.6-1.6)
		Yes	306	32.7	23.6	9.1 (-1.5-20.1)
24 – 30	Low	No	14408	48.5	50.5	-2 (-4.4-0.3)
		Yes	2633	32.7	32.1	0.6 (-4.9-6.3)
	Middle	No	4486	49.5	50.8	-1.3 (-5.3-2.7)
		Yes	777	34.4	36.5	-2.1 (-11.7-8)
	High	No	3237	46.3	49.3	-3 (-7.6-1.7)
		Yes	318	41.4	36.6	4.8 (-9-19.7)

For peer review only

Supplementary material 8

Sensitivity analysis only including women with a recent health care contact (defined as any dispensed prescription or appointment at a hospital in the last 3 years) = 60.46% of the original population

Table 1. Characteristics of the 553 789 women aged 12 - 30 years and residing in Sweden by 1st January 2013 by previous mental health issues and use of hormonal contraceptives. Values are percentages (number of women) if not otherwise indicated.

	Previous mental health issues			
	Yes 19.01 (n = 105 283) Use of Hormonal contraceptives		No 80.99 (n = 448 506) Use of Hormonal contraceptives	
	Yes 42.42 (n = 44657)	No 57.58 (n = 60 626)	Yes 44.41 (n= 199 170)	No 55.59 (n = 249 336)
Antidepressant drugs	41.17 (19 886)	39.77 (26 013)	2.73 (9 215)	1.87 (8 699)
Age				
12-17 years	15.11 (6 747)	20.75 (12 581)	15.63 (31 133)	37.24 (92 846)
18-23 years	48.78 (21 784)	31.29 (18 968)	48.30 (96 200)	22.75 (56 735)
24-30 years	36.11 (16 126)	47.96 (29 077)	36.07 (71 837)	40.01 (99 755)
Income level				
Low	39.70 (17 731)	45.01 (27 286)	33.06 (65 847)	35.08 (87 456)
Middle	27.55 (12 302)	27.85 (16 887)	26.00 (51 776)	29.91 (74 575)
High	32.75 (14 624)	27.14 (16 453)	40.94 (81 547)	35.01 (87 305)
Immigrant background				
No	94.50 (42 200)	88.94 (53 919)	93.76 (186 745)	83.61 (208 465)
Yes	5.50 (2 457)	11.06 (6 707)	6.24 (12 425)	16.39 (40 871)

For peer review only - http://bmjopen.bmj.com/site/about/guidelines.xhtml

Table 2. Results from the Multilevel Analysis of Individual Heterogeneity and Discriminatory Accuracy (MAIHDA) distinguishing between measures of association and measures of variance and discriminatory accuracy. The analyses are stratified by the existence of previous mental issues. Values are point estimations with (95% Confidence Intervals)

	Without metal health issues		With mental health issues	
	Model 1	Model 2	Model 1	Model 2
**Measures of association,** Odds Ratios				
Age				
12-17		Reference		Reference
18-23		1.73 (1.33-2.22)		1.52 (1.33-1.71)
24-30		1.90 (1.48-2.40)		2.58 (2.29-2.91)
Income				
High		Reference		Reference
Middle		1.16 (0.91-1.48)		0.89 (0.79-1.01)
Low		1.17 (0.92-1.55)		0.89 (0.78-1.01)
Immigrant background				
No		Reference		Reference
Yes		0.65 (0.53-0.81)		0.55 (0.49-0.61)
Hormonal contraceptives				
No		Reference		Reference
Yes		1.40 (1.12-1.71)		1.18 (1.06-1.34)
**Measures of variance**				
Variance*	0.224 (0.130-0.372)	0.077 (0.038-0.141)	0.287 (0.174-0.468)	0.017 (0.008-0.033)
VPC	6.38%	2.29%	8.02%	0.51%
PCV		65.67%		94.09%
AUC	0.61 (0.61-0.61)	0.61 (0.61-0.61)	0.64 (0.64-0.64)	0.64 (0.64-0.64)

*Between-strata variance, variance partition coefficient (VPC), proportional change of the variance (PCV), Area under the curve (AUC)

For peer review only - http://bmjopen.bmj.com/site/about/guidelines.xhtml

Table 3. Absolute risk (AR) of antidepressant use, and AR difference (ARD) between user and non-users of hormonal contraceptives but otherwise sharing the same intersectional stratum. The values are calculated from the multilevel analysis of individual heterogeneity and discriminatory accuracy (MAIHDA)

Previous mental health issues	Age (years)	Income level	Immigrant background	Number of women	Use of hormonal contraceptive		
					Yes AR	No AR	Yes-No difference ARD
No	12 – 17	Low	No	14060	4.4	1.8	2.7 (2 - 3.4)
			Yes	3123	1.5	0.8	0.8 (-0.1 - 2)
		Middle	No	37376	3.6	1.3	2.2 (1.8 - 2.6)
			Yes	4543	2.4	1.0	1.4 (0.3 - 2.8)
		High	No	62712	2.4	1.1	1.3 (1 - 1.5)
			Yes	2165	2.8	1.2	1.7 (0.4 - 3.3)
	18 – 23	Low	No	25939	4.2	3.4	0.8 (0.3 - 1.2)
			Yes	5720	2.5	2.0	0.5 (-0.3 - 1.4)
		Middle	No	40241	3.3	3.6	-0.3 (-0.6 - 0.1)
			Yes	4547	2.7	1.8	0.9 (0 - 1.9)
		High	No	74281	2.7	2.9	-0.2 (-0.4 - 0.1)
			Yes	2207	2.0	2.7	-0.6 (-1.9 - 0.5)
	24 – 30	Low	No	83448	3.6	3.7	0 (-0.3 - 0.2)
			Yes	21013	2.9	2.1	0.8 (0.3 - 1.4)
		Middle	No	31818	4.0	3.5	0.5 (0.1 - 0.9)
			Yes	7826	3.0	2.7	0.3 (-0.5 - 1.2)
		High	No	25335	2.9	3.0	-0.1 (-0.5 - 0.3)
			Yes	2152	2.5	2.7	-0.2 (-1.5 - 1.2)
Yes	12 – 17	Low	No	3371	30.1	22.3	7.8 (4.7 - 10.9)
			Yes	429	20.4	13.4	7 (-0.4 - 14.8)
		Middle	No	6787	31.0	23.1	7.9 (5.7 - 10.1)
			Yes	565	19.5	14.3	5.2 (-1.5 - 12.7)
		High	No	7807	33.8	27.7	6.1 (3.9 - 8.2)
			Yes	369	29.7	19.4	10.3 (1.2 - 19.6)
	18 – 23	Low	No	10205	38.8	36.9	1.9 (0.1 - 3.8)
			Yes	1068	28.1	19.0	9.1 (3.5 - 14.6)
		Middle	No	12082	36.6	35.0	1.6 (-0.1 - 3.3)
			Yes	805	27.2	19.1	8.1 (2 - 14.3)
		High	No	15994	37.2	38.5	-1.3 (-2.8 - 0.3)
			Yes	598	26.3	25.4	0.8 (-6.1 - 7.7)
	24 – 30	Low	No	26185	49.2	48.9	0.3 (-0.9 - 1.6)
			Yes	3759	36.0	32.6	3.4 (-0.3 - 7)
		Middle	No	7820	49.2	50.4	-1.3 (-3.6 - 1)
			Yes	1130	31.4	37.2	-5.8 (-12.4 - 0.9)

For peer review only - http://bmjopen.bmj.com/site/about/guidelines.xhtml

High	No	5868	47.8	47.8	0 (-2.6 - 2.6)
	Yes	441	41.3	35.2	6.1 (-3.6 - 16)

For peer review only

For peer review only - http://bmjopen.bmj.com/site/about/guidelines.xhtml

Reporting checklist for cohort study.

Based on the STROBE cohort guidelines.

Instructions to authors

Complete this checklist by entering the page numbers from your manuscript where readers will find each of the items listed below.

Your article may not currently address all the items on the checklist. Please modify your text to include the missing information. If you are certain that an item does not apply, please write "n/a" and provide a short explanation.

Upload your completed checklist as an extra file when you submit to a journal.

In your methods section, say that you used the STROBE cohortreporting guidelines, and cite them as:

von Elm E, Altman DG, Egger M, Pocock SJ, Gotzsche PC, Vandenbroucke JP. The Strengthening the Reporting of Observational Studies in Epidemiology (STROBE) Statement: guidelines for reporting observational studies.

		Reporting Item	Page Number
**Title and abstract**			
Title	#1a	Indicate the study's design with a commonly used term in the title or the abstract	1
Abstract	#1b	Provide in the abstract an informative and balanced summary of what was done and what was found	2
**Introduction**			
Background / rationale	#2	Explain the scientific background and rationale for the investigation being reported	4-5
Objectives	#3	State specific objectives, including any prespecified hypotheses	5
**Methods**			
Study design	#4	Present key elements of study design early in the paper	5

For peer review only

Setting	#5	Describe the setting, locations, and relevant dates, including periods of recruitment, exposure, follow-up, and data collection	5-6
Eligibility criteria	#6a	Give the eligibility criteria, and the sources and methods of selection of participants. Describe methods of follow-up.	5-6
Eligibility criteria	#6b	For matched studies, give matching criteria and number of exposed and unexposed	5-6
Variables	#7	Clearly define all outcomes, exposures, predictors, potential confounders, and effect modifiers. Give diagnostic criteria, if applicable	6-7
Data sources / measurement	#8	For each variable of interest give sources of data and details of methods of assessment (measurement). Describe comparability of assessment methods if there is more than one group. Give information separately for for exposed and unexposed groups if applicable.	6-7
Bias	#9	Describe any efforts to address potential sources of bias	6-7
Study size	#10	Explain how the study size was arrived at	5-6
Quantitative variables	#11	Explain how quantitative variables were handled in the analyses. If applicable, describe which groupings were chosen, and why	7-8
Statistical methods	#12a	Describe all statistical methods, including those used to control for confounding	

6-7

Statistical methods	#12b	Describe any methods used to examine subgroups and interactions	7-8
Statistical methods	#12c	Explain how missing data were addressed	7-8
Statistical methods	#12d	If applicable, explain how loss to follow-up was addressed	n/a Follow-up was complete

For peer review only

			for the final cohort
Statistical methods	#12e	Describe any sensitivity analyses	
14-15			
**Results**			
Participants	#13a	Report numbers of individuals at each stage of study—eg numbers potentially eligible, examined for eligibility, confirmed eligible, included in the study, completing follow-up, and analysed. Give information separately for for exposed and unexposed groups if applicable.	5-6
Participants	#13b	Give reasons for non-participation at each stage	5-6
Participants	#13c	Consider use of a flow diagram	
Figure 1			
Descriptive data	#14a	Give characteristics of study participants (eg demographic, clinical, social) and information on exposures and potential confounders. Give information separately for exposed and unexposed groups if applicable.	9, table 1
Descriptive data	#14b	Indicate number of participants with missing data for each variable of interest	
n/a All participants included in final analysis had complete data			
Descriptive data	#14c	Summarise follow-up time (eg, average and total amount)	
5-6			
Outcome data	#15	Report numbers of outcome events or summary measures over time. Give information separately for exposed and unexposed groups if applicable.	
9-10			

For peer review only

Main results	#16a	Give unadjusted estimates and, if applicable, confounder-adjusted estimates and their precision (eg, 95% confidence interval). Make clear which confounders were adjusted for and why they were included	10-11
Main results	#16b	Report category boundaries when continuous variables were categorized	11-12
Main results	#16c	If relevant, consider translating estimates of relative risk into absolute risk for a meaningful time period	
10-12			
Other analyses	#17	Report other analyses done—eg analyses of subgroups and interactions, and sensitivity analyses	11-13
**Discussion**			
Key results	#18	Summarise key results with reference to study objectives	13-14
Limitations	#19	Discuss limitations of the study, taking into account sources of potential bias or imprecision. Discuss both direction and magnitude of any potential bias.	15-16
Interpretation	#20	Give a cautious overall interpretation considering objectives, limitations, multiplicity of analyses, results from similar studies, and other relevant evidence.	16
Generalisability	#21	Discuss the generalisability (external validity) of the study results	16
**Other Information**			
Funding	#22	Give the source of funding and the role of the funders for the present study and, if applicable, for the original study on which the present article is based	17

Notes:

- 12d: n/a Follow-up was complete for the final cohort

For peer review only

- 14b: n/a All participants included in final analysis had complete data The STROBE checklist is distributed under the terms of the Creative Commons Attribution License CC-BY. This checklist was completed on 26. January 2021 using https://www.goodreports.org/, a tool made by the EQUATOR Network in collaboration with Penelope.ai

For peer review only
